# Supplementary material for: Effectiveness and Components of Health Behavior Interventions on Increasing Physical Activity Among Healthy Young and Middle-Aged Adults: A Systematic Review with Meta-Analyses
Source: Behav Sci (Basel). 2024 Dec 19;14(12):1224. doi: 10.3390/bs14121224 (PMC11673272; doi:10.3390/bs14121224)
Supplement: Supplementary file 1 [file behavsci-14-01224-s001.zip › S1_Sup_Query.pdf]

Supplementary Table S1. Each part of the search query for PubMed.

Part	Content	Search query
Q1	Physical activity relative outcomes	exercise[MeSH Major Topic] OR exercise[tiab] OR physical activity[tiab] OR "sedentary behavior"[MeSH Major Topic] OR "sedentary behavior"[tiab] OR “sitting”[tiab]
Q2	Intervention	"health education"[MeSH Major Topic] OR "health education"[tiab] OR "health promotion"[tiab] OR "healthy people programs"[tiab] OR "physical education and training"[MeSH Major Topic] OR "physical education"[tiab] OR "physical training"[tiab] OR "intervention*"[tiab] OR “approach*”[tiab] OR "wellness program*"[tiab] OR “strateg*”[tiab] OR planned[tiab] OR preplanned[tiab]
Q3	Participant characteristics	adult[MeSH Major Topic] OR “adult*”[tiab] OR "middle aged"[tiab] OR "working adult*"[tiab] OR "working age"[tiab] OR “worker*”[tiab] OR “employee*”[tiab] OR company[tiab] NOT “patient*”[tiab] NOT child[tiab] NOT adolescent[tiab] NOT elderly[tiab] NOT “older adult*”[tiab]
Q4	Study design	"randomized controlled trial"[pt] OR "controlled clinical trial"[pt] OR "randomized controlled trial"[tiab] OR "controlled clinical trial"[tiab] NOT “study protocol”[tiab] NOT ”systematic review”[tiab]

Note: the full search query for PubMed was combined from Q1, Q2, Q3 and Q4 by string "AND".

Supplementary Table S2. Each part of the search query for Ichu-shi Web.

Part	Content	Search query
Q1	Physical activity relative keywords	(身体運動/TH or 運動活性/TH or 身体活動量の少ない生活/TH or 座位/TH) and (PT=原著論文)
Q2	Intervention	(健康教育/TH or 健康増進/TH or 労働衛生/TH or 地域社会健康教育イベント/TH or 体育とトレーニング/TH or 健康増進センター/TH or 身体調整活動/TH) and (PT=原著論文)
Q3	Study design	(介入研究/TH) and (PT=原著論文)

Note: the full search query for Ichushi-web was combined from Q1, Q2 and Q3 by string "AND".

Supplementary Table S3. The meanings of Japanese keywords in English.

Keyword in Japanese	Keyword in English
身体運動	Exercise
身体活性	Physical activity
身体活動量の少ない	Sedentary behavior
座位	Sitting
健康教育	Health education
健康増進	Health promotion
労働衛生	Health promotion
地域社会健康教育イベント	Health promotion
体育とトレーニング	Physical education
健康増進センター	Wellness
身体調整活動	Physical training
介入研究	Randomized controlled trial
原著論文	Original Article

Note: since health promotion is classified under various categories in the Ichu-shi web, we used three keywords: "健康増進" (health promotion), "労働衛生" (occupational health), and "地域社会健康教育イベント" (community health education events).
