# Supplementary material for: Effectiveness and Components of Health Behavior Interventions on Increasing Physical Activity Among Healthy Young and Middle-Aged Adults: A Systematic Review with Meta-Analyses
Source: Behav Sci (Basel). 2024 Dec 19;14(12):1224. doi: 10.3390/bs14121224 (PMC11673272; doi:10.3390/bs14121224)
Supplement: Supplementary file 1 [file behavsci-14-01224-s001.zip › S2_Sup_ResInfo.pdf]

Supplementary Table S4. Information of included studies.

Authors and year	Country	Study design	Intervention duration, weeks	Group	Details	Baseline n (women % in n)	Baseline age (SD) or [95%CI], years	BCT	Measurement (method)
Aadahl M et al. 2014	Australia	RCT	24	Control (CON)	Maintained usual lifestyle.	73 (49)	51.8 (14.3)	None	Overall: Sedentary time (device and self-report) Walking time (device) VPA (self-report)<br><br>Leisure: Sedentary time (self-report)<br><br>Work: Sedentary time (self-report)
				Intervention	4 theory-based face-to-face sessions over 6 months focusing on reducing sitting time through goal setting, self-efficacy, and motivational interviewing.	93 (63)	52.2 (13.8)	1.3, 4.1, 5.1	
Aldana SG et al. 2005	United States	RCT	4 (measurement at 6-week)	Control (CON)	Screened at baseline and deferred intervention for 6 months.	180 (71)	50.8 (11.1)	10.3	Overall: Walking step (device)
				Intervention	4-week, 16-session program with live lectures, workbooks, professional talks, and practical activities focused on diet, exercise, and chronic disease prevention.	186 (73)	50.4 (11.1)	1.1, 3.1, 4.1, 5.1	
Allen A et al. 2018	United States	RCT	12	Control (CON)	Maintained their current activity level.	11 (82)	31.0 (2.2)	None	Overall: Total PA EE (self-report)

Authors and year	Country	Study design	Intervention duration, weeks	Group	Details	Baseline n (women % in n)	Baseline age (SD) or [95%CI], years	BCT	Measurement (method)
				CA	3 weekly 30-minute sessions following a "Couch to 5k" program, gradually increasing from walking to jogging.	9 (78)	29.2 (1.8)	6.1	Walking step (device)
				HIIT	Weekly 20-minute HIIT session on a stationary bike, progressively increasing intensity over weeks.	12 (33)	30.4 (1.5)	6.1	
Allen JK et al. 2013	United States	RCT	24	IC (CON)	Weekly counseling for 1 month, biweekly during month 2-6, focusing on diet and exercise goals.	18 (78)	42.5 (12.1)	1.1, 3.1	Overall: MVPA time (self-report)
				SP (CON)	1 session on nutrition and app training, using the app for self-management and real-time feedback.	17 (71)	45.3 (13.2)	1.1, 2.3	
				IC + SP	IC + SP	16 (81)	45.6 (9.3)	1.1, 2.3, 3.1	
				LIC+SP	2 counseling sessions in the first month, then monthly sessions during month 2-6 + SP	17 (77)	46.4 (9.6)	1.1, 2.3, 3.1	

Authors and year	Country	Study design	Intervention duration, weeks	Group	Details	Baseline n (women % in n)	Baseline age (SD) or [95%CI], years	BCT	Measurement (method)
Allman-Farinelli M et al. 2016	Australia	RCT	12	Control (CON)	4 text messages on key behaviors over 12 weeks, a 2-page handout, access to a basic website, and one introductory call.	125 (63)	27.2 (4.9)	5.1, 7.1	Overall: Total PA EE and frequency (self-report)
				TXT2BFiT	4 text messages on key behaviors over 12 weeks, a 2-page handout, access to a basic website, and one introductory call. After 12 weeks, received low dose maintenance phase with monthly texts and emails, website access, and 2 booster calls.	123 (59)	28.1 (4.9)	2.3, 3.1, 5.1, 7.1	
Almhdawi KA et al. 2020	Jordan	RCT	6	CG (CON)	Accessed to traditional medical care and placebo app.	20 (20	41.70 (6.35)	2.3, 3.1	Overall: Total PA EE (self-report)
				EG	Accessed to traditional medical care and experimental app with general advice, office/home exercises, and notifications for posture, exercise, and walking breaks.	21 (34)	40.48 (7.22)	2.3, 3.1, 7.1	
Alsaleh E 2023	Jordan	RCT	24	Control (CON)	Attended monthly educational sessions on PA health benefits, risks of inactivity, and strategies to increase PA, with a focus on healthy living aspects.	70 (51)	21.2 (2.3)	3.2	Overall: MVPA frequency, time and EE (self-report)
				Intervention	Received individualized consultations, weekly motivational SMS reminders, and Facebook page support to enhance PA awareness, self-efficacy, and integration into daily routines.	76 (54)	21.3 (2.4)	1.1, 2.3, 3.2	Walking frequency and time (self-report) Walking step (device)

Authors and year	Country	Study design	Intervention duration, weeks	Group	Details	Baseline n (women % in n)	Baseline age (SD) or [95%CI], years	BCT	Measurement (method)
An LC et al. 2013	United States	RCT	12	General lifestyle content (CON)	6 weekly sessions of general interest topics (e.g., music, finances, movies) with email reminders and $10 weekly incentives to visit the study website.	567 (74)	24.14 (no reported SD)	3.1, 5.1, 7.1, 10.2	Overall: Exercise frequency (self-report)
				Tailored health messages	6-week program with weekly focus on different health behaviors.	566 (73)	23.96 (no reported SD)	7.1, 10.2	
				Tailored message plus peer coach	Similar to Tailored health messages with additional online peer coaching. Weekly video messages and phone calls from peer coaches.	563 (71)	24.12 (no reported SD)	5.1, 7.1, 10.2	
Andersen LL et al. 2013	Denmark	RCT	10	Control (CON)	Weekly email reminders to maintain usual physical activities.	54 (76)	43 (11)	7.1	Leisure: LPA, MPA, VPA time (self-report)
				Email	Weekly email reminders to walk stairs for 10 minutes daily, with additional advice on scheduling, group formation, and varying intensity.	84 (79)	42 (10)	7.1	
Annesi JJ 2012	United States	RCT	26	Nutrition Education (CON)	6 one-hour group sessions over 3 months focusing on healthy eating principles and menu planning.	N=183 in Nutrition Education, N=247 in	42.5 (10.0) in all participants.	4.1	Leisure: Total PA EE (self-report)

Authors and year	Country	Study design	Intervention duration, weeks	Group	Details	Baseline n (women % in n)	Baseline age (SD) or [95%CI], years	BCT	Measurement (method)
				Cognitive-Behavior Nutrition Education	6 one-hour sessions with emphasis and all exercise support components.	Cognitive-Behavior Nutrition Education; women % = 83 in all participants		1.1, 5.5, 13.2	
Annesi JJ et al. 2015	United States	RCT	12	Self-efficacy treatment (CON)	6 sessions of exercise support and 6 sessions focusing on mastery of controlled, healthy eating, personal role models, persistence, and managing negative feelings.	N=138 in Self-efficacy treatment, N=136 in	42.5 (10.0) in all participants.	4.1, 6.1	Leisure: Total PA EE (self-report)
				Self-regulation treatment	6 sessions of exercise support and 6 sessions focusing on self-regulatory skills for overcoming barriers to healthy eating.	Self-regulation treatment; women % = 81 in all participants		4.1, 6.1	
Appel LJ et al. 2003	United States	RCT	24	Advice Only (CON)	30-minute session with a dietitian discussing nonpharmacological blood pressure factors.	273 (63)	49.5 (8.8)	3.1	Overall:Total PA EE (self-report)
				Established	Focused on reducing total and saturated fat intake with 18 face-to-face contacts over 6 months, including group meetings and individual sessions.	268 (65)	50.2 (8.6)	1.3, 2.3	
				Established + DASH	Same as Established with additional emphasis on the DASH diet.	269 (57)	50.2 (9.3)	1.3, 2.3, 3.1, 4.1	

Authors and year	Country	Study design	Intervention duration, weeks	Group	Details	Baseline n (women % in n)	Baseline age (SD) or [95%CI], years	BCT	Measurement (method)
Ashton LM et al. 2017	Australia	RCT	12	Control (CON)	Continued usual lifestyle for 3 months.	24 (0)	21.9 (2.1)	None	Overall:Walking step (device)MVPA time (self-report)
				Intervention	Received a comprehensive intervention including a responsive website with resources, app, and weekly face-to-face sessions.	26 (0)	22.4 (2.0)	2.2, 2.3, 3.1, 4.1	
Assunção MC et al. 2010	Brazil	RCT	24	CG (CON)	Received standard guidance from a nutritionist based on individual consultations.	121 (88)	39.6 (1.1)	None	Leisure: Total PA time (self-report)
				IG	Underwent a multi-component intervention including promoting healthy diet habits and PA.	120 (90)	41.1 (1.2)	3.1	
Ball K et al. 2005	Australia	RCT	12	Print (CON)	Received standard print intervention with newsletters and motivational prizes.	36 (74)	57.0 (8.0)	2.3, 4.1, 5.1	Overall: MVPA EE (self-report)
				Telephone	Received the same print intervention as the control group, plus 6 telephone calls from trained staff.	30 (73)	52.3 (5.0)	2.3, 4.1, 5.1, 7.1	
Beleigoli A et al. 2020	Australia	RCT	24	Waiting list (CON)	Received a basic e-booklet and videos on health and behavior, with no active intervention until the end of the trial.	470 (77)	33.4 [32.4, 34.4]	1.2, 1.3, 1.4, 4.1, 4.2, 5.1, 12.1	Overall: MPA and VPA time (self-report)

Authors and year	Country	Study design	Intervention duration, weeks	Group	Details	Baseline n (women % in n)	Baseline age (SD) or [95%CI], years	BCT	Measurement (method)
				Platform only	Used a web-based weight loss program with educational content, self-monitoring, and interactive tools. Personalized feedback was provided from week 4.	420 (75)	34.4 [33.4, 35.6]	1.1, 1.2, 1.3, 1.4, 1.5, 1.7, 2.2, 2.3, 2.4, 3.1, 3.2, 3.3, 4.1, 4.2, 5.1, 5.6, 11.2, 12.1, 12.2, 12.3, 13.2, 15.1, 16.3	
				Platform plus coaching	Similar to Platform-Only program, plus 12 weeks of personalized feedback and support from a dietitian via a private chat forum.	408 (78)	33.0 [31.9, 34.0]	1.1, 1.2, 1.3, 1.4, 1.5, 1.7, 2.2, 2.3, 2.4, 3.1, 3.2, 3.3, 4.1, 4.2, 5.1, 5.6, 11.2, 12.1, 12.2, 12.3, 13.2, 15.1, 16.3	
Benito PJ et al. 2020	Spain	RCT	22	C (CON)	Adhered to PA recommendations.	29 (66)	37.4 (8.1) in participants who	3.1	Overall: Sedentary time and PA EE (self-report)

Authors and year	Country	Study design	Intervention duration, weeks	Group	Details	Baseline n (women % in n)	Baseline age (SD) or [95%CI], years	BCT	Measurement (method)
				S	Performed strength exercises over 22 weeks.	30 (63)	completed the study	6.1	
				E	Engaged in running, cycling, or elliptical workouts over 22 weeks.	30 (63)		6.1	
				SE	S+E	30 (53)		6.1	
Bergman F et al. 2018	Sweden	RCT	50	Control (CON)	Continued usual office work at their desk.	40 (55)	50.3 (6.7)	None	Overall: Walking time and step (device) Sedentary time (device) LPA and MVPA time (device)
				Intervention	Received a treadmill workstation, instructed to walk at a self-chosen pace for at least 1 hour/day, and email reminders and health information.	40 (55)	52.4 (6.8)	7.1, 12.1	
Biddle SJ et al. 2015	United Kingdom	RCT	12	Control (CON)	Received an information leaflet on T2D risk, PA, and sedentary behavior.	93 (66.7)	33.3 (5.8)	5.1	Overall: Sedentary time (device and self-report)

Authors and year	Country	Study design	Intervention duration, weeks	Group	Details	Baseline n (women % in n)	Baseline age (SD) or [95%CI], years	BCT	Measurement (method)
				Intervention	Attended a 3-hour education workshop, received a self-monitoring device for PA, and had a follow-up call at 6 weeks.	94 (70.2)	32.4 (5.4)	2.3, 4.1, 7.1	Walking step (device) Total PA frequency (Device) and EE (self-report) LPA and MPA time (device)
Blake H et al. 2019	United Kingdom	Cluster-RCT	12	Control (CON)	In wait-list.	86 (43.0	Range 25–40 years	None	Overall: Total PA and sedentary time (self-report)
				Intervention	12-week digital Qigong exercise program with twice-daily, 10-minute sessions. Videos co-created with team leaders; supported by on-screen prompts. Team leaders guided sessions, with support from a Qigong Master.	196 (49.5)		1.2 , 1.4, 3.1, 4.1, 5.1, 6.1, 8.3	
Brakenridge CL et al. 2016	Australia	Cluster-RCT	12	ORG (CON)	Received bi-weekly emails with tips to stand, walk more, and reduce sitting	87 (40)	40.0 (8.0)	3.1, 7.1	Overall: Sedentary time (device) Walking time

Authors and year	Country	Study design	Intervention duration, weeks	Group	Details	Baseline n (women % in n)	Baseline age (SD) or [95%CI], years	BCT	Measurement (method)
				ORG + Tracker	ORG + an activity tracker	66 (53)	37.6 (7.8)	2.3, 3.1, 7.1	and step (device)<br><br>Work: Sedentary time (device)<br>Walking time and step (device)
Cho AR et al. 2019	Republic Of Korea	RCT	12	Control (CON)	Continued regular eating and exercise habits.	5 (40)	42.6 (10.6)	None	Overall:Total PA EE (self-report)
				E-ADF	Followed ADF protocol plus resistance and aerobic exercise 3 times/week.	9 (44)	34.5 (5.7)	1.1, 2.3, 4.1	
				ADF (Excluded from analysis for not PA-relative intervention)	Alternated between fasting (25% daily intake) and ad libitum feeding every other day.	8 (75)	33.5 (5.0)	1.1	
				Exercise	Performed resistance and aerobic exercise 3 times/week.	9 (44)	38.6 (8.2)	2.3, 4.1	

Authors and year	Country	Study design	Intervention duration, weeks	Group	Details	Baseline n (women % in n)	Baseline age (SD) or [95%CI], years	BCT	Measurement (method)
Cleo G et al. 2019	Australia	RCT	12	WL (CON)	Continued as usual.	25 (80)	52.0 (12.3)	3.1	Overall: Exercise time (self-report)
				TTT	Received program with 10 tips for weight loss.	25 (80)	48.2 (11.3)	2.3, 3.1, 5.1, 8.3	
				DSD	Received program to break unhealthy habits by doing novel daily tasks.	25 (76)	51.3 (10.0)	2.3, 3.1, 8.3	
Compton SE et al. 2022	United States	RCT	24	10,000 Steps (CON)	Achieve 10,000 steps daily for at least 6 days a week, plus Biweekly step data reviews.	31 (100)	18.5 (0.5)	1.1, 1.2, 2.2, 2.3, 12.5	Overall: Walking step (device)
				12,500 Steps	Achieve 12,500 steps daily for at least 6 days a week, plus Biweekly step data reviews.	27 (100)	18.7 (0.4)	1.1, 1.2, 2.2, 2.3, 12.5	
				15,000 Steps	Achieve 15,000 steps daily for at least 6 days a week, plus Biweekly step data reviews.	21 (100)	18.4 (0.6)	1.1, 1.2, 2.2, 2.3, 12.5	
Conroy MB et al. 2015	United States	RCT	12	SG (CON)	Received a 12-week self-guided program.	49 (100)	54.0 (5.6)	5.1, 10.1	Overall: Total PA EE (self-report)
				IL	12 weekly group sessions led by a physician or interventionist, focused on diet, PA, and stress relief.	49 (100)	53.8 (5.3)	1.1, 1.3, 3.1, 4.1, 5.1, 10.1	

Authors and year	Country	Study design	Intervention duration, weeks	Group	Details	Baseline n (women % in n)	Baseline age (SD) or [95%CI], years	BCT	Measurement (method)
Creasy SA et al. 2022	United States	RCT	15	AM Exercise (CON)	Aerobic exercise in morning (06:00-10:00).	18 (78)	40.8 (8.4)	1.1, 1.2, 1.4, 3.2, 4.1, 6.1, 7.1, 8.1, 8.3	Overall: Sedentary time (device)
				PM Exercise	Aerobic exercise in evening (15:00-19:00)..	15 (60)	36.4 (10.8)	1.1, 1.2, 1.4, 3.2, 4.1, 6.1, 7.1, 8.1, 8.3	
Daly RM et al. 2020	Australia	RCT	16	Ex + placebo (CON)	Completed a 4-month supervised resistance, balance, and mobility training program with 2 gym sessions per week and 1 home session starting in week 5, while consuming a placebo supplement.	121 (100)	56 (5)	6.1	Overall: MVPA time (self-report)
				Ex + MFMD	Ex + placebo + consuming a daily milk-based supplement.	123 (100)	55 (5)	6.1	
Davy BM et al. 2017	United States	RCT	12-week training + RCT with 48-week intervention	Standard (CON)	3-month initiation phase of supervised resistance training, and 6-month standard care resistance training intervention.	80 (71)	59.7 (5.1)	3.1	Overall: Total PA EE (self-report)
				SCT	3-month initiation phase of supervised resistance training, and 6-month SCT-based RT intervention.	79 (73)	59.6 (5.6)	3.1, 6.1	

Authors and year	Country	Study design	Intervention duration, weeks	Group	Details	Baseline n (women % in n)	Baseline age (SD) or [95%CI], years	BCT	Measurement (method)
Dorling JL et al. 2021	United States	RCT	12	Control (CON)	Maintained their typical work and lifestyle habits.	20 (70)	46.7 (9.8)	None	Overall:Sedentary, LPA, MVPA time (device)Walking step (device)
				Intervention	Provided Pedal Desk to increase PA.	20 (85)	46.2 (11.4)	1.1, 3.1	
Duggan C et al. 2014	United States	RCT	12	Delayed Intervention (CON)	Was given the intervention after 12 weeks.	166 (72)	50.8 (12.9)	None	Leisure: Total PA, MPA and VPA frequency (Self-report)
				Immediate Intervention	Five weekly 1-hour home-based diabetes education sessions.	154 (69)	50.3 (12.4)	4.1	
Dunn AL et al. 1998	United States	RCT	24	Lifestyle (CON)	Attended weekly small group sessions with a facilitator, focusing on cognitive-behavioral strategies to integrate moderate PA into daily life, progressing at their own pace.	121 (50)	45.9 (6.8)	1.1, 3.1, 4.1	Overall: Sedentary and walking time (self-report) Total PA and MPA EE (self-report)
				Structured	Received a free gym membership, an ACSM exercise prescription, and supervised exercise sessions, progressing to independent exercise.	114 (51)	46.2 (6.5)	1.1, 3.1	

Authors and year	Country	Study design	Intervention duration, weeks	Group	Details	Baseline n (women % in n)	Baseline age (SD) or [95%CI], years	BCT	Measurement (method)
Dunn AL et al. 1999	United States	RCT	96	Lifestyle (CON)	6 months of weekly group sessions teaching cognitive and behavioral strategies for moderate-intensity activity, transitioning to less frequent meetings with varied activities over 18 months, supported by monthly calendars and newsletters.	121 (50)	45.9 (6.8)	1.1, 3.1, 4.1	Overall: Total PA, MPA and VPA EE (self-report) Sedentary and walking time (self-report)
				Structured exercise	6 months of supervised aerobic exercise at a fitness center, with monthly follow-up meetings and quarterly newsletters during 18 months of maintenance.	114 (51)	46.2 (6.5)	1.1, 1.4, 3.1, 5.1, 8.3	
Eaglehouse YL et al. 2016	United States	Cluster-RCT	24	Delayed-start (CON)	In wait-list.	N=223 (62.3) in all participants.	58.4 (11.5) in all participants.	None	Leisure: Total PA EE (self-report)
				Immediate-start	The 12-month DPP-GLB program aimed for 150 minutes of moderate PA weekly and 7% weight loss. Participants could choose group or DVD sessions, with follow-up bi-weekly/monthly meetings. Coaching was provided by trained lifestyle coaches.			1.1, 1.3, 2.2, 2.3	
Edwardson CL et al. 2018	United Kingdom	Cluster-RCT	48	Control (CON)	Received health measurement results but no lifestyle intervention	67 (87)	40.8 (11.3)	2.6, 4.4	Overall: Sedentary, walking and

Authors and year	Country	Study design	Intervention duration, weeks	Group	Details	Baseline n (women % in n)	Baseline age (SD) or [95%CI], years	BCT	Measurement (method)
				Intervention	Used height-adjustable desks, attended educational seminars, received real-time feedback, and had quarterly coaching sessions to reduce sitting.	77 (73)	41.7 (11.0)	1.1, 1.5, 3.1, 5.1, 12.1	MVPA time (device)
Finkelstein EA et al. 2016	Singapore	RCT	24	Control (CON)	Received educational booklets.	201 (56)	35.6 (8.6)	5.1	Overall: MVPA frequency (device) Walking step (device)
				Fitbit	Received Fitbit Zip and access to the Fitbit website, plus weekly participation payments.	203 (49)	35.4 (8.3)	1.1, 2.3, 3.2, 5.1	
				Charity	Same as Fitbit group, plus weekly incentives (S$15 or S$30) donated to charity based on step count.	199 (54)	35.5 (8.6)	1.1, 2.3, 3.2, 5.1, 10.4	
				Cash	Same as Charity group, but weekly incentives were paid directly to participants instead of charity.	197 (57)	35.5 (8.4)	1.1, 2.3, 3.2, 5.1, 10.2	
Fischer X et al. 2019	Switzerland	RCT	24	Control (CON)	Received a single written recommendation and an online PA plan with no further contact with the study team.	96 (33)	42.20 (11.39)	1.1, 1.2, 1.4, 1.5, 2.2, 2.5, 3.1, 5.1, 8.1	Overall: MVPA time (device and self-report)
				Coaching	Received 12 bi-weekly phone calls focusing on BCTs, goal-setting, planning PA, and overcoming barriers with a trained coach.	99 (70)	41.93 (11.12)	1.1, 1.2, 1.4, 1.5, 2.2, 2.5, 3.1, 5.1, 8.1	

Authors and year	Country	Study design	Intervention duration, weeks	Group	Details	Baseline n (women % in n)	Baseline age (SD) or [95%CI], years	BCT	Measurement (method)
				Coaching and SMS	Coaching + 4 tailored SMS prompts every 2 weeks related to BCTs, feedback, or reminders.	93 (69)	42.54 (11.78)	1.1, 1.2, 1.4, 1.5, 2.2, 2.5, 2.7, 3.1, 5.1, 8.1	
Fukuoka Y et al. 2015	United States	RCT	20	Control (CON)	Received pre-diabetes brochure and no further intervention.	31 (77)	53.4 (8.7)	5.1	Overall: Walking step (device) LPA, MPA and VPA time (device)
				Intervention	5-month program with in-person sessions and a mobile app for weight loss, increased activity, and reduced calorie intake.	30 (77)	57.1 (9.1)	2.3, 2.4, 3.1	
Furukawa F et al. 2003	Japan	RCT	12	CG (CON)	Received given information on walking, and continued with normal daily activities.	25 (100)	42.1 (6.9)	4.1	Overall: Total PA and exercise EE (device) Walking step (device)
				IG	Implemented a 12-week systematic walking program with individual planning, and planned their exercise schedule, aiming for 5 kcal/kg body mass per day.	24 (100)	40.8 (5.1)	1.4, 1.5, 2.2, 2.3, 3.1, 4.1, 8.3	
Genin PM et al. 2017	France	RCT	20	CON (CON)	No treatment.	N=22 in CON, N=37 in NOVICE,	44 (9.9) in all participants.	None	Overall: Total PA EE (self-report)

Authors and year	Country	Study design	Intervention duration, weeks	Group	Details	Baseline n (women % in n)	Baseline age (SD) or [95%CI], years	BCT	Measurement (method)
				NOVICE	Engaged in worksite physical activity program at the study's onset, with two 45-minute sessions per week.	N=36 in EXP; women % = 35 in all participants.		1.4, 3.1, 8.1, 8.3	Sedentary time (self-report)
				EXP	Engaged in the worksite physical activity program for at least 2 years, with a minimum of two 45-minute sessions per week.			1.4, 3.1, 8.1, 8.3	
Gill DP et al. 2019	Canada	RCT	24	Comparator (CON)	Continued with their usual activities	59 (81)	58.6 (14.7)	None	Overall:Walking step (self-report)Total PA EE (self-report)Sedentary time (self-report)
				Intervention	Received personalized coaching to set and achieve exercise and dietary goals, used various eHealth tools, and had progressively reduced support over 18 months.	59 (76)	56.8 (12.3)	1.1, 1.2, 1.4, 2.3, 3.1, 4.1, 5.1, 9.1, 12.2	
Gomersall SR et al. 2015	Australia	RCT	6	Control (CON)	Wait-listed, continued usual routine.	N=37 in each gourp; women % = 64 in all participants	42 (10)	None	Overall: Sedentary, LPA, MVPA and total PA EE (self-report)
				Moderate	150 minutes/week PA, half in supervised group classes and half individually.		41 (12)	1.1, 1.7, 2.1, 3.1, 9.3, 12.6	
				Extensive	Same as moderate, but with 300 minutes/week PA.		45 (10)	1.1, 1.7, 2.1, 3.1, 9.3, 12.6	

Authors and year	Country	Study design	Intervention duration, weeks	Group	Details	Baseline n (women % in n)	Baseline age (SD) or [95%CI], years	BCT	Measurement (method)
Gorin AA et al/ 2013	United States	RCT	72	BWL (CON)	Weekly meetings for 6 months, then bi-weekly. Participants followed a 1200–1800 kcal/day diet and increased exercise to 200 minutes/week. Core behavioral skills were taught with daily diaries and pedometer goals.	Participants: 99 (79)<br><br>Partners: 99 (48)	Participants: 50.4（9.3）<br><br>Partners: 47.9 (13.3)	1.1, 2.3, 2.7, 3.1, 4.1, 5.1, 10.1	Overall: Total PA EE (self-report)
			72	BWL+H	BWL + home environment modifications like treadmill or bike, food cleanout exercises, and reduced TV time. Household partners also participated in the program.	Participants 102 (78)<br><br>Partners: 102 (46)	Participants: 47.5（11.3）<br><br>Partners: 47.8 (13.0)	1.1, 2.3, 2.7, 3.1, 4.1, 5.1, 8.3, 10.1, 12.1	
Grey EB et al. 2019	United Kingdom	RCT	12	CON (CON)	Were shown three NHS healthy living websites for advice on diet and exercise but had no further interaction until offered the Evolife intervention after 12 weeks.	29 (45)	49.5 (9.1)	3.1	Overall: Sedentary time, LPA MVPA time (Device)
				INT	Used a website to set personal health goals, guided by evolutionary mismatch theory. They received a pedometer and personalized feedback on diet and activity.	30 (43)	50.3 (8.9)	1.1, 1.2, 1.4, 2.2, 2.3, 3.2, 3.3, 4.1, 4.2, 6.1, 8.3, 12.2, 15.2	Total PA EE (Device) Walking step (Device)
Harrington DM et al. 2014	United States	RCT	12	DG (CON)	Weekly group sessions and biweekly phone calls focused on diet changes with food diary discussions.	52 (75)	50.6 (8.2)	2.3, 2.5, 3.1, 5.1	Overall: MVPA time (Device)
				DG+PA	DG + a focus on increasing daily steps to 30 minutes of moderate activity, using pedometers for tracking and goal-setting.	47 (89)	53.3 (7.5)	2.3, 2.5, 3.1, 5.1	

Authors and year	Country	Study design	Intervention duration, weeks	Group	Details	Baseline n (women % in n)	Baseline age (SD) or [95%CI], years	BCT	Measurement (method)
Healy GN et al. 2016	Australia	Cluster-RCT	12	Control (CON)	Received written feedback after intervention.	95 (73)	47.0 (9.7)	None	Overall: Sedentary, walking time (device)
				Intervention	Multicomponent intervention including organizational strategies, environmental strategies, and individual strategies.	136 (65)	44.6 (9.1)	1.1, 1.7, 3.1, 12.1	Work: Sedentary, walking time (device)
Hivert MF et al. 2007	Canada	RCT	96	Control (CON)	No intervention.	57 (82)	19.5(0.2)	None	Overall: Total PA EE (self-report)
				Intervention	Attended 23 small-group seminars over 2 years covering weight management, diet, exercise, and behavioral strategies.	58 (81)	19.9(0.2)	1.1, 1.2, 1.4, 2.3, 4.1, 5.1	
Hunter JR et al. 2008	Australia	RCT	8	CON (CON)	20-30 minutes of aerobic and resistance exercises, without individual supervision each session.	25 (80)	42.8 (4.9)	2.1, 8.3	Overall: Total PA EE (self-report)
				SUP	20-30 minutes of aerobic and resistance exercises, with individual supervision each session.	25 (80)	42.2 (4.3)	2.1, 4.1, 6.1, 8.3	
Hurling R et al. 2007	United Kingdom	RCT	9	Control (CON)	Received verbal advice on physical activity, with no access to the behavior change system and no feedback.	30 (70)	40.1 (7.7)	12.1	Overall: Total PA EE (self-report) Sedentary time

Authors and year	Country	Study design	Intervention duration, weeks	Group	Details	Baseline n (women % in n)	Baseline age (SD) or [95%CI], years	BCT	Measurement (method)
				Test	Used an Internet-based behavior change system for 9 weeks, with initial accelerometer data collection and demonstration.	47 (64)	40.5 (7.1)	1.2, 1.4, 1.5, 2.2, 2.3, 4.1, 6.1, 7.1	(self-report) Leisure: Total PA EE (self-report)
Jakicic JM et al. 2016	United States	RCT	96	SBWI (CON)	Regular group sessions, prescribed diet, self-monitored PA via website, and monthly phone support.	233 (71)	Median (25th, 75th percentile):30.9 (28.0, 33.9)	2.3, 2.6, 3.1, 5.1, 10.1	Overall:Sedentary, LPA, MVPA time (device)Walking step (device)MVPA EE (device)
				EBWI	Similar to SBWI, but with additional wearable technology for self-monitoring and feedback via a web interface.	237 (71)	Median (25th, 75th percentile): 31.0 (27.4, 33.3)	2.3, 2.5, 2.6, 3.1, 5.1, 10.1	
Jakicic JM et al. 2015	United States	RCT	72	ADOPT (CON)	Weekly group sessions, dietary and PA prescriptions, bi-weekly phone calls, supervised sessions, and 9-month PA campaigns.	49 (76)	44.65 (8.26)	1.1, 1.8, 2.2, 2.4, 2.7, 3.1, 10.1	Overall: Total PA EE and frequency (self-report)
				MAINTAIN	Weekly group sessions, dietary and PA prescriptions, bi-weekly phone calls, supervised sessions, and PA campaigns.	44 (75)	46.44 (6.50)	1.1, 1.8, 2.2, 2.4, 2.7, 3.1, 10.1	
				SBWP	Weekly group sessions, dietary and PA prescriptions.	47 (83)	42.86 (9.35)	1.1, 1.8, 2.2, 2.4, 2.7, 3.1, 10.1	
Jakicic JM et al. 2022	United States	RCT	48	DIET (CON)	Prescribed calorie intake based on weight, maintained PA at current levels, recorded food intake in a diary reviewed by intervention staff.	127 (80)	44.3 (8.0)	3.1, 4.1, 7.3	Overall: Total PA EE (self-report)

Authors and year	Country	Study design	Intervention duration, weeks	Group	Details	Baseline n (women % in n)	Baseline age (SD) or [95%CI], years	BCT	Measurement (method)
				DIET+MODPA	Prescribed calorie intake, started PA at 100 minutes/week, increased to 150 minutes/week over time, recorded PA in a diary, received feedback from intervention staff.	129 (79)	46.8 (7.6)	1.1, 1.4, 2.2, 2.3, 3.1, 4.1, 7.3, 8.1, 8.3	
				DIET+HIGHPA	Prescribed calorie intake, started PA at 100 minutes/week, increased to 250 minutes/week over time, recorded PA in a diary, received feedback from intervention staff.	127 (80)	45.6 (8.1)	1.1, 1.4, 2.2, 2.3, 3.1, 4.1, 7.3, 8.1, 8.3	
Jamal SN et al. 2016	Malaysia	RCT	24	Diet counselling (CON)	Underwent individual counseling with a dietician once every 12 weeks.	97 (74)	40.4 (9.5)	None	Overall: Total PA, VPA, MPA and walking EE (self-report)
				GSLiM	Group sessions with 10 meetings, targeting a 6% weight loss, including seminars and practical sessions on diet, exercise, and behavior change.	97 (71)	39.7 (9.2)	2.3, 3.1, 4.1	
Johnston V et al. 2019	Australia	RCT	4	Desk Only (CON)	Used a sit-stand workstation.	13 (54)	40 (11)	12.1	Overall: Sendentary time (device)
				Desk+Exercise	Used a sit-stand workstation, and performed a standardized progressive resistance exercise program monitored by a physiotherapist.	16 (81)	39 (11)	4.1, 6.1, 8.3, 12.1	

Authors and year	Country	Study design	Intervention duration, weeks	Group	Details	Baseline n (women % in n)	Baseline age (SD) or [95%CI], years	BCT	Measurement (method)
Juul L et al. 2016	Denmark	RCT	48	Control (CON)	In wait-list.	64 (70)	Age, median (q1, q3) 60 (51, 64)	None	Overall: Total PA EE (self-report)
				Intervention	Received four 2-hour sessions in five weeks and two follow-ups at one and six months, focusing on transformative learning and health literacy.	63 (67)	Age, median (q1, q3) 58 (50, 63)	1.1, 3.1, 5.1, 6.1	
Kanaya AM et al. 2012	United States	RCT	24	Control (CON)	Waited until completing the trial.	117 (74)	55 (17)	None	Overall: Total PA time and EE (self-report) Walking time (self-report)
				Intervention	The program included 12 telephone calls, 2 in-person sessions, and 5 optional group workshops over 12 months, focusing on diet and PA with goal-setting and motivational interviewing.	113 (73)	58 (16)	1.1, 1.4, 3.1	
Kim JY et al. 2015	Republic Of Korea	RCT	24	Control (CON)	4 education sessions, brief counseling, monthly weight checks for 6 months.	95 (0)	41.55 (6.98)	5.1	Overall: Total PA EE (self-report)
				Text message	Tailored text messages every other day, 4 education sessions, brief counseling, monthly weight checks for 6 months.	101 (0)	41.02 (6.82)	1.1, 1.2, 4.1, 5.1, 6.1	

Authors and year	Country	Study design	Intervention duration, weeks	Group	Details	Baseline n (women % in n)	Baseline age (SD) or [95%CI], years	BCT	Measurement (method)
Kleist B et al. 2017	Germany	RCT	12	DI (CON)	Hypoenergetic diet (500-800 kcal/day) for 12 weeks.	N=44 in DI, N=38 in DI + walking; women % = 56 in all participants.	38.9 (8.2) in men, 39.9 (7.5) in women.	1.1, 3.1, 4.1, 6.1, 7.1	Overall: Total PA EE (self-report)
				DI + walking	DI + 2.5 hours/week walking program for 12 weeks.			1.1, 3.1, 4.1, 6.1, 7.1, 8.1, 8.3	
Kolt GS et al. 2017	Australia	RCT	72	Logbook (CON)	Received a paper-based logbook with goal-setting instructions and activity tracking.	171 (63)	50.8 (13.1) in all participants	1.1, 3.1	Overall: MVPA time (device)
				Web 1.0	Used the existing 10,000 Steps program, featuring an online step log, pedometer, self-monitoring tools, and educational materials to increase PA.	165 (65)		2.3, 3.1, 5.1	
				Web 2.0	Participants accessed a new website with social networking features, including friend lists, private messaging, activity updates, and virtual walking groups, in addition to the 10,000 Steps features.	168 (68)		1.1, 3.1	
Lewis E et al. 2019	Australia	Crossover-RCT	16 + 16(Only data of baseline to 16-week was used)	C-TS (CON)	Standard OMS care, including a multidisciplinary team, 4-week education group, individualized case management, and periodic medical assessments.	32 (75)	Mean (range)50 (31–74)	1.1, 3.1, 4.1, 5.1	Overall:Total PA EE (device)

Authors and year	Country	Study design	Intervention duration, weeks	Group	Details	Baseline n (women % in n)	Baseline age (SD) or [95%CI], years	BCT	Measurement (method)
				TS-C	Monthly 10–30 min personalized calls focused on goal setting, problem solving, and motivation for PA, diet, and lifestyle changes.	29 (79)	Mean (range) 49 (25–72)	1.1, 1.2, 2.3, 3.1, 7.1, 8.3	
Lim SL et al. 2022	Singapore	RCT	24	Control (CON)	Received standard face-to-face dietary advice, a digital weighing scale for self-monitoring, and encouragement to engage in 150 minutes/week of moderate physical activity.	76 (40)	54.3 (9.9)	1.1, 2.3, 3.1, 4.1, 12.5	Overall: Total PA time (self-report)
				Intervention	Control + was introduced to the nBuddy Diabetes app for 6 months to self-monitor weight, diet, physical activity, and blood glucose, with automated feedback, educational videos, and remote dietitian coaching via the app.	72 (40)	51.9 (8.7)	1.1, 1.2, 2.2, 2.3, 2.6, 3.1, 3.2, 4.1, 7.1	
Liukkonen M et al. 2017	Finland	Cluster-RCT	48	Group A (CON)	Received separate test results.	42 (19)	42 (10)	2.6	Overall: Total PA EE (self-report)
				Group B	Received Body Age index in years and separate test results.	43 (19)	43 (10)	2.6	
				Group C	Received Body Age index, separate test results, and a training computer (Polar FT60) for voluntary training.	36 (25)	40 (10)	2.6	

Authors and year	Country	Study design	Intervention duration, weeks	Group	Details	Baseline n (women % in n)	Baseline age (SD) or [95%CI], years	BCT	Measurement (method)
Lombard C et al. 2010	Australia	Cluster-RCT	48	Control (CON)	A non-interactive information session on dietary and physical activity guidelines.	123 (100)	40.26 (4.80)	2.3, 4.1, 5.1, 6.1, 12.1	Overall: Walking, MPA, VPA EE (self-report) Walking step (device)
				Intervention	4 interactive group sessions with health messages and behavior change strategies, plus monthly support via text messages for 12 months.	127 (100)	40.56 (4.75)	1.1, 1.4, 1.5, 2.2, 4.1, 5.1, 7.1, 8.1	
Looyestyn J et al., 2018	Finland	RCT	8	Control (CON)	Self-directed running program with a tracking magnet, no Facebook access.	48 (71)	35.1 (10.9)	2.3, 4.1, 6.1, 10.8	Overall: MVPA time (self-report)
				Intervention	8-week beginner's running program via a closed Facebook group, with daily posts and a tracking magnet.	41 (90)	35.3 (11.2)	2.3, 3.1, 4.1, 6.1, 10.8	
Low V et al. 2015	United States	RCT	24	Control (CON)	Accessed to risk reduction classes, an on-site gym, and organized walks.	29 (100)	53 (6.0)	3.1, 4.1, 8.1	Overall: Exercise frequency (self-report)
				Intervention	Weekly communication with goal-setting and obstacle strategies for 6 months, alongside access to risk reduction classes, an on-site gym, and organized walks.	28 (100)	51 (6.5)	1.1, 3.1, 4.1, 7.1, 8.1, 8.3	
Lugones-Sanchez C et al. 2022	Spain	RCT	12	CG (CON)	Received 5 minutes of lifestyle counseling from a trained nurse on physical activity and diet, following international guidelines.	332 (70)	48.9 (9.2)	3.1, 4.1	Overall: LPA, MPA,VPA, MVPA, total

Authors and year	Country	Study design	Intervention duration, weeks	Group	Details	Baseline n (women % in n)	Baseline age (SD) or [95%CI], years	BCT	Measurement (method)
				IG	CG + 3-month low-intensity intervention with the EVIDENT 3 app and a smart band for daily self-monitoring of food intake and physical activity, focusing on a hypocaloric Mediterranean diet and achieving 10,000 steps/day.	318 (67)	47.7 (10.1)	1.1, 1.2, 2.3, 3.1, 4.1, 5.1, 10.4, 12.5	PA, sedentary time (self-report)
Maddison R et al. 2014	New Zealand	RCT	24	Control (CON)	Usual smoking cessation support from Quitline.	451 (54)	37.3 (12.2)	3.1, 10.1	Overall: Walking, MPA, VPA EE (self-report)<br><br>Leisure: Total PA EE (self-report)<br><br>Work: Total PA EE (self-report)<br><br>Transport: Total PA EE (self-report)
				Intervention	Control + 6-month home/community-based exercise program with 10 counseling sessions aimed at promoting 30 minutes of moderate-vigorous exercise most days.	455 (54)	37.6 (12.2)	1.2, 2.2, 3.1, 7.1, 10.1	
Madjd A et al. 2019	Iran	RCT	24	SBP (CON)	Diet plus two daily 25-minute brisk walking sessions with a PA partner, pedometer tracking, and weekly dietitian/exercise coach visits.	32 (100)	27.9 (4.4)	1.1, 1.2, 2.2, 2.3, 2.6, 3.1, 4.1, 5.1	Overall: Walking step (device)
				LBP	Same as SBP, but with 50-minute brisk walking session.	33 (100)	27.6 (4.7)	1.1, 1.2, 2.2, 2.3, 2.6, 3.1, 4.1, 5.1	

Authors and year	Country	Study design	Intervention duration, weeks	Group	Details	Baseline n (women % in n)	Baseline age (SD) or [95%CI], years	BCT	Measurement (method)
Maher C et al. 2015	Australia	Cluster-RCT	20	Control (CON)	Waited until completing the trial.	59 (76)	Age in years, n (%) 18 to <25 12 (24)25 to <35 17 (33)35 to <45 12 (24)45 to 65 10 (20)	None	Overall:MVPA, VPA, walking time (self-report)
				Intervention	50-day Facebook app with team-based step tracking, 10,000 daily step goal, peer support, gamification, and weekly emails, plus a pedometer.	51 (73)	Age in years, n (%) 18 to <25 14 (24) 25 to <35 15 (25) 35 to <45 17 (29) 45 to 65 9 (15)	1.1, 2.3, 3.1, 4.1, 5.1, 7.1, 10.4	

Authors and year	Country	Study design	Intervention duration, weeks	Group	Details	Baseline n (women % in n)	Baseline age (SD) or [95%CI], years	BCT	Measurement (method)
Mansi S et al. 2015	New Zealand	RCT	12	Control (CON)	Standard educational materials.	29 (52)	43 (14.9)	5.1, 6.1	Overall: Walking step and time (device) Total PA EE (self-report)
				Intervention	12-week pedometer-driven walking with weekly step goals, feedback, emails, step diary, and a goal to reach 10,000 steps/day.	29 (65)	40 (12.2)	1.1, 2.2, 2.3, 5.1, 6.1, 7.1	
Marston KJ et al. 2019	Australia	RCT	12	Control (CON)	Maintained usual lifestyle.	15 (80)	59.1 (7.4)	None	Overall: Total PA EE (self-report)
				Moderate-load group	Moderate-load resistance training, 3 sets of 10 reps at 70% 1RM, 60s rest between sets.	15 (80)	58.0 (5.5)	6.1	
				High-load group	High-load resistance training, 5 sets of 5 reps at 85% 1RM, 180s rest between sets.	14 (86)	55.2 (6.8)	6.1	
Martin CK et al. 2019	United States	RCT	24	Control (CON)	No exercise.	61 (74)	49.5 (10.8)	None	Overall: Total PA, MPA and VPA time (self-report)
				8 KKW	Exercise at 8 KKW (~700 kcal/week), self-selected intensity 65%-85% VO2peak,	59 (73)	48.3 (11.2)	1.1, 6.1	

Authors and year	Country	Study design	Intervention duration, weeks	Group	Details	Baseline n (women % in n)	Baseline age (SD) or [95%CI], years	BCT	Measurement (method)
				20 KKW	Exercise at 20 KKW (~1760 kcal/week), ramped up from 8 KKW to 20 KKW by week 3, self-selected intensity 65%-85% VO2peak.	51 (71)	48.7 (12.4)	1.1, 6.1	
Maruyama C et al. 2010	Japan	Crossover-RCT	16 (Only duration before wash-out was reported)	Control (CON)	No treatment.	47 (0)	35.5 (8.1)	None	Overall: Walking step (device)
				LiSM	Received LiSM10!® program including monthly counseling, dietary and physical activity goal-setting, self-monitoring via website, online support.	52 (0)	43.1 (7.7)	1.1, 2.3, 5.1, 6.1	
Maylor BD et al. 2018	United States	Cluster-RCT	8	Control (CON)	Waited until completing the trial.	41 (64)	43.7 [39.7, 47.7]	None	Overall: Sedentary time (device) Walking time and step (device)
				Intervention	Multicomponent intervention including educational presentation and brainstorming session , step challenge, health check report and individual meetings, prompts, telephone support and improving work environment.	48 (54)	43.0 [39.4, 46.7]	1.1, 1.2, 1.5, 2.3, 2.6, 3.2, 5.1, 5.2, 6.1, 7.1, 8.2, 8.4, 10.2, 10.4, 12.1	Work: Sedentary time (device) Walking time and step (device)
McRobbie H et al. 2016	United Kingdom	RCT	48	Nurse (CON)	Four one-on-one sessions over 8 weeks with standard diet and activity advice and motivational support.	109 (69)	45.1 (14.2)	3.1, 4.1, 5.1	Overall: Total PA EE (self-report)

Authors and year	Country	Study design	Intervention duration, weeks	Group	Details	Baseline n (women % in n)	Baseline age (SD) or [95%CI], years	BCT	Measurement (method)
				WAP	Eight weekly group sessions on diet, physical activity, and self-monitoring, followed by 10 monthly maintenance sessions.	221 (73)	46.6 (15.0)	1.1, 1.2, 1.4, 1.5, 2.2, 2.3, 3.1, 4.1, 7.1, 8.1, 8.5, 8.6, 8.7, 10.2, 10.8, 11.1, 11.2, 12.1, 13.1, 15.4, 16.2	Sedentary time (self-report)
Merom D et al. 2007	Australia	RCT	12	Control (CON)	No treatment.	123 (85)	49.0 (9.8)	None	Overall:Walking time and frequency (self-report)MVPA and total PA time (self-report)Leisure: Walking time and frequency (self-report)Exercise and total PA time (self-report)
				WP	Self-help booklet "Step by Step," weekly diaries, incremental walking stages, self-assessment.	123 (85)	49.2 (9.3)	4.1, 5.1, 6.1	
				WWP	WP + pedometer.	123 (85)	49.3 (8.6)	2.3, 4.1, 5.1, 6.1	
Meyer JD et al. 2018	United States	RCT	8	Control (CON)	No treatment.	17 (89)	49.6 (11.2)	None	Overall: MVPA time (device)

Authors and year	Country	Study design	Intervention duration, weeks	Group	Details	Baseline n (women % in n)	Baseline age (SD) or [95%CI], years	BCT	Measurement (method)
				AET	8 weeks of aerobic exercise sessions and daily at-home practice, with a half-day retreat.	14 (79)	55.9 (8.3)	1.1, 2.3, 4.1, 8.1	
				MBSR	8 weeks of weekly mindfulness sessions and daily practice, with a 6-h retreat.	18 (78)	50.9 (12.5)	1.1, 2.3, 3.2, 3.3, 4.1	
Miller CK et al. 2015	United States	RCT	16	Control (CON)	Received an information booklet regarding lifestyle changes for diabetes prevention.	33 (79)	50.8 (8.14)	None	Overall:Walking step (device)
				Exper.	Received the 16-week Group Lifestyle Balance intervention adapted from the individually administered DPP	35 (80)	51.6 (9.51)	1.1, 1.2, 2.2, 2.3, 3.2, 3.3, 4.1, 5.1, 6.1	
Monroe CM et al. 2023	United States	RCT	12	TECH (CON)	Received a Fitbit Alta HR, set progressive step goals, accessed a website with behavior change content, and received weekly personalized feedback.	59 (78)	39.7 (8.6)	1.1, 1.2, 1.4, 1.5, 2.2, 2.3, 3.1, 4.1	Overall: Walking step (device) MVPA time (device)
				TECH + Gamification	TECH + engaged in team-based gamification, including a step competition and the Shoe Mascot Game, where team progress was displayed on a leaderboard.	57 (79)	40.6 (11.9)	1.1, 1.2, 1.4, 1.5, 2.2, 2.3, 3.1, 4.1, 6.2, 8.7, 10.1, 10.4, 14.2	

Authors and year	Country	Study design	Intervention duration, weeks	Group	Details	Baseline n (women % in n)	Baseline age (SD) or [95%CI], years	BCT	Measurement (method)
Morgan PJ et al. 2011	Australia	RCT	14	Control (CON)	In wait-list.	45 (0)	43.7 (9.1)	None	Overall: Total PA EE (self-report)
				WP program	3-month Workplace POWER program based on Social Cognitive Theory with a 75-minute information session, study website for weight and diet tracking with feedback, weight loss resources, and group-based financial incentives.	65 (0)	44.8 (8.3)	1.1, 2.2, 2.3, 3.2, 5.1, 6.1, 8.3, 10.1, 10.2	
Mueller J et al. 2022	United Kingdom	RCT	16	Standard advice (CON)	Received standard advice via an EASO leaflet on diet, physical activity, and mood during the COVID-19 pandemic	196 (78)	49.9 (13.2)	1.4, 5.1, 9.1	Overall: Total PA EE (self-report)
				SWiM-C	Accessed a 12-week web-based ACT-guided self-help platform with psychoeducation, exercises, and coach support through two semi-structured calls and tailored emails, plus weekly self-monitoring of weight.	192 (79)	50.7 (14.3)	1.1, 1.2, 2.3, 3.1, 3.2, 3.3, 7.1, 8.1, 8.3	
Nakata Y et al. 2022	Japan	RCT	12	Control (CON)	Continued current lifestyle	69 (26)	44.0 (9.1)	None	Overall: Walking step (device) MVPA time (device)
				Intervention	Used CALO mama Plus app to log daily weight, diet, exercise, mood, and sleep quality; received personalized advice based on logged data, with reminders via email or phone if input frequency was low.	72 (26)	42.3 (9.4)	1.1, 1.2, 1.5, 2.2, 2.3, 3.1, 4.1, 7.1	

Authors and year	Country	Study design	Intervention duration, weeks	Group	Details	Baseline n (women % in n)	Baseline age (SD) or [95%CI], years	BCT	Measurement (method)
Newton RL Jr et al. 2004	United States	RCT	24	PA (CON)	Minimal intervention with initial physician advice and monthly meetings on unrelated health topics.	N=11 in PA, N=27 in SB, N=22 in CS; Women % = 81 in all participants.	47.3 (7.4)	3.1, 5.1, 6.1	Overall: Exercise frequency (self-report)
				SB	10 sessions over 6 months focusing on goal setting, self-monitoring, and problem-solving, led by Caucasian counselors in a university setting.		44.0 (7.0)	1.1, 1.2, 2.3, 4.1, 5.1, 6.1	
				CS	Similar to SB, but tailored for African-Americans with African-American counselors, culturally relevant materials, and sessions in the African-American community.		45.0 (7.8)	1.1, 1.2, 2.3, 3.1, 4.1, 5.1, 6.1	
Nishimura M et al. 2019	Japan	RCT	8	Control (CON)	Wore an activPAL3VT without any feedback or report.	13 (62)	51 (9)	None	Overall: Walking time (device)
				Vibration	Wore an activPAL3VT monitor on the thigh for 9 hours daily, and received vibration feedback when sedentary for 30 minutes, with weekly reports of sedentary patterns.	13 (85)	51 (10)	2.2	
Obling KH et al. 2019	Denmark	RCT	24	Routine Care (CON)	Received routine care, including a one-hour motivational interview and an optional follow-up interview at a health center.	117 (38)	42.0 [36.0, 47.0]	4.1, 5.1	Overall:Sedentary, LPA, MVPA time (device)

Authors and year	Country	Study design	Intervention duration, weeks	Group	Details	Baseline n (women % in n)	Baseline age (SD) or [95%CI], years	BCT	Measurement (method)
				Intervention	Participants joined a sports club with a 6-month free membership, received a GPS watch for self-monitoring on Endomondo.com, and had four motivational interviews to promote physical activity.	115 (42)	41.0 [37.0, 46.0]	1.1, 1.2, 2.2, 2.3, 3.1, 3.2, 4.1, 5.1, 6.2, 12.5, 12.6	
Oftedal S et al. 2019	Australia	RCT	4	Wait-List (CON)	Maintained usual lifestyle habits.	20 (45)	36.6 (10.1)	10.1	Overall: MVPA time (self-report)
				Intervention	Used the Balanced app for goal setting and self-monitoring of physical activity, diet, and sleep, received weekly summary reports, a handbook for behavior change, and weekly SMS tips.	20 (60)	34.9 (9.2)	1.1, 2.2, 2.3, 5.1, 7.1, 10.1	
Østbye T et al. 2015	United States	RCT	56	WM (CON)	Recieved 7 coaching sessions, including optional diet and physical activity consultations, with participation calculated based on completed face-to-face or phone sessions.	275 (83)	Age group, yrs, n (%) <35, 53 (19); 35–50, 134 (49); >50, 88 (32).	2.6, 3.1, 4.1, 5.1, 6.1	Overall: MVPA and sedentary time (device)

Authors and year	Country	Study design	Intervention duration, weeks	Group	Details	Baseline n (women % in n)	Baseline age (SD) or [95%CI], years	BCT	Measurement (method)
				WM+	Similar to WM, but with 12 coaching contacts.	275 (84)	Age group, yrs, n (%) <35, 42 (15); 35–50, 133 (48); >50, 100 (36).	1.1, 1.2, 2.6, 3.1, 3.2, 3.3, 4.1, 5.1, 6.1	
Park KS et al. 2024	Republic Of Korea	RCT	12	CG (CON)	A health education brochure was provided	39 (0)	40.6 (3.9)	5.1	Overall: Total PA EE (self-report)
				IG	Remote PA program via Zoom for 12 weeks, including health education, exercise training, small group meetings, individual counseling, and daily tailored text messages for lifestyle reflection and exercise encouragement.	39 (0)	39.3 (3.8)	1.1, 1.2, 2.2, 2.3, 3.1, 4.1, 5.1, 6.1, 7.1, 8.3	
Pereira MA et al. 2020	United States	Cluster-RCT	48	Move+ (CON)	Multilevel intervention targeting reduction in sedentary time and increases in LPA.	253 (84)	45.6 (11.4)	1.1, 3.1, 4.1, 12.1, 12.2	Overall: Sedentary, LPA and MVPA time (device)
				Stand+	Move+ + sit-stand workstations for alternating between sitting and standing while working.	276 (63)	43.3 (10.8)	1.1, 3.1, 4.1, 12.1, 12.2	
Pesola AJ et al. 2017	Finland	Cluster-RCT	24	Control (CON)	No treatment during the intervention duration.	62 (52)	39.6 (5.3)	None	Overall: Sendentary, LPA, MVPA time (device)

Authors and year	Country	Study design	Intervention duration, weeks	Group	Details	Baseline n (women % in n)	Baseline age (SD) or [95%CI], years	BCT	Measurement (method)
				Intervention	Participants attended a 30-min lecture on reducing sitting time and increasing light activity, followed by goal-setting discussions, and received phone counseling at 2 and 5 months to review and adjust goals.	71 (60)	36.6 (5.1)	1.1, 1.2, 3.2, 3.3, 5.1, 5.2	Work: Sendentary, LPA, MVPA time (device)
Peterson TR et al. 1999	United States	RCT	6	Control (CON)	Did not receive any materials.	N=185 in Control, N=169 in Generic, N=174 in Staged; women % = 60.4 in all participants.	Younger than 45 years of age (79.3%)	None	Overall: Total PA EE (self-report)
				Generic	Received a generic intervention with information on the benefits of exercise and recommended activity levels, based on the Surgeon General's report.			4.1, 5.1	
				Staged	Received stage-specific messages with information and exercises tailored to their readiness for change, including goal-setting and relapse prevention activities, aligned with the transtheoretical model.			1.1, 1.2, 3.3, 4.1	
Phaswana M et al. 2023	South Africa	RCT	12	Control (CON)	Continued using traditional desks.	18 (22)	36.3 (11.2)	None	Overall: Sedentary, LPA, MVPA time (device)
				Intervention	Received height-adjustable sit-to-stand workstations, trained on usage, encouraged to reduce sitting time with standing bouts, received regular researcher visits and communication.	44 (78)	41.9 (9.4)	1.1, 3.1, 4.1, 5.1, 8.7, 12.1	

Authors and year	Country	Study design	Intervention duration, weeks	Group	Details	Baseline n (women % in n)	Baseline age (SD) or [95%CI], years	BCT	Measurement (method)
Plotnikoff RC et al. 2023	Australia	Cluster-RCT	36	Control (CON)	Continued with their regular activities.	123 (74)	54.1 (13.6)	None	Overall: MVPA time (device) MVPA, total PA EE (self-report)
				Intervention	90-minute introductory session, accessed to ecofit app for outdoor gym workouts, was encouraged to complete 2 workouts/week, and joined ecofit Facebook group for social support.	122 (71)	52.8 (14.3)	1.1, 1.2, 2.3, 3.1, 8.1, 8.3, 12.2	
Poirier J et al. 2016	United States	RCT	6	Control (CON)	Maintained their daily activity routine	132 (70)	39.6 (12.0)	None	Overall: Walking step (device)
				Intervention	Registered for Walkadoo, installed a USB dongle and synchronization software, and recieved visual feedback on their activity tracker for continuous data syncing at work and home.	133 (62)	40.3 (11.4)	1.1, 2.3, 3.1, 4.1, 10.4, 10.5	
Puig-Ribera A et al. 2008	Spain	RCT	9	Control (CON)	Maintained usual walking behavior.	N=26 in Control, N=19 in Walking routes, N=25 in Walking in tasks; women % = 72 in all participants.	men, 39 (8); women, 40 (18)	None	Overall: Walking step (device)
				Walking routes	Received campus walk maps, encouraged 15 minutes of brisk walking daily			1.1, 1.2, 2.3, 3.1, 4.1	
				Walking in tasks	Was guided to increase step counts during office tasks, standing meetings, supported by weekly emails with goal-setting and strategies to overcome barriers.			1.1, 1.2, 2.3, 3.1, 4.1	

Authors and year	Country	Study design	Intervention duration, weeks	Group	Details	Baseline n (women % in n)	Baseline age (SD) or [95%CI], years	BCT	Measurement (method)
Puig-Ribera A et al. 2015	Spain	Cluster-RCT	19	Control (CON)	Maintained habitual behavior.	N=135 in Control, N= 129 in Intervention ; women % = 65 in all participants.	42 (10) in all participants.	None	Overall: Walking step (device) sedentary time (self-report)
				Intervention	Accessed to W@WS, a web-based program to reduce sitting and increase walking at work over 19 weeks through step goals, automated emails, and support strategies.			1.1, 1.2, 1.5, 2.3, 3.3, 4.1, 5.1, 7.1	
Ribeiro MA et al. 2014	Australia	RCT	12	MTC (CON)	Attended three 15-minute monthly sessions, receiving general advice about PA and a booklet on how to increase PA in daily life.	47 (100)	45 (3)	4.1, 6.1	Overall: Walking step (device)
				Ped IC	Received the same intervention as MTC plus a pedometer to monitor steps, with a goal to increase daily steps by 2000, and a diary to record their steps.	54 (100)	45 (3)	1.1, 2.3, 4.1, 6.1	
				Ped GC	Attended eight group counseling sessions, learning about PA benefits, overcoming barriers, and using pedometers, with a goal to increase daily steps by 2000 and group walking sessions.	48 (100)	45 (3)	1.1, 2.3, 3.3, 4.1, 5.1	
				AT	Performed 24 treadmill aerobic exercise sessions over 3 months, increasing duration from 30 to 40 minutes, with moderate to intense exertion.	47 (100)	45 (3)	3.2	

Authors and year	Country	Study design	Intervention duration, weeks	Group	Details	Baseline n (women % in n)	Baseline age (SD) or [95%CI], years	BCT	Measurement (method)
Richardson CR et al. 2010	United States	RCT	16	No Online Community Arm (CON)	Participants used a web-based walking program with pedometers.	70 (66)	53.3 (11.8)	2.4, 2.7, 3.2, 3.3, 10.2	Overall: Walking step (device)
				With Online Community Arm	Same as No Online Community Arm, with additional access to an online community for posting and reading messages.	254 (64)	51.7 (11.3)	2.4, 2.7, 3.2, 3.3, 10.2, 10.5	
Richardson CR et al. 2016	United States	RCT	24	Time-Based (CON)	Set time-based walking goals, reviewed by dietitians during MNT sessions, with gradual increases in daily walking time.	85 (0)	55.0 (9.8)	1.3, 2.4, 3.1, 5.1, 10.1	Overall: MVPA time (device)
				Simple Pedometer	Same as Time-Based, but used a pedometer to manually log and review daily step counts, with step goals increased by 10%-25%.	86 (0)	58.0 (9.9)	1.3, 2.4, 3.1, 5.1, 10.1	
				Enhanced Pedometer	Same as Simple Pedometer, but used an enhanced pedometer with automated step logging, online feedback, and access to an online community for motivation.	84 (0)	55.9 (10.3)	1.3, 2.4, 2.7, 3.1, 5.1, 10.1, 10.5	
Rosas LG et al. 2020	United States	RCT	48	Usual care (CON)	Continued to receive usual care from their primary care clinicians.	99 (61)	50.1 (12.0)	3.1	Overall: Total PA EE (self-report)<br>Leisure: MVPA time and total PA EE (self-report)
				Intervention	Usual care + 1-year culturally adapted Group Lifestyle Balance program led by a bilingual health coach, involving group sessions, use of activity trackers and diet apps, and family-oriented support, followed by a second-year email-based support.	92 (62)	50.3 (12.5)	1.1, 1.2, 2.2, 2.3, 3.1, 4.1, 7.1	

Authors and year	Country	Study design	Intervention duration, weeks	Group	Details	Baseline n (women % in n)	Baseline age (SD) or [95%CI], years	BCT	Measurement (method)
Rovniak LS et al. 2005	United States	RCT	12	LowFidelity (CON)	Attended an orientation session, received a walking manual and log, and were instructed to walk three times per week for 30 minutes while self-monitoring and reporting their activity via email.	30 (100)	Range 20-54 years	1.1, 2.2, 2.3, 4.1, 6.1	Overall: Walking time (self-report)
				HighFidelity	Similar to LowFidelity, but with additional specific walking speed goals, tailored feedback, modeling demonstrations, and tools like a wrist stopwatch and pre-measured walking routes.	31 (100)		1.1, 2.2, 2.3, 4.1, 6.1	
Ruusunen A et al. 2012	Finland	RCT	144	Control (CON)	Received general diet and exercise advice aimed at weight reduction and increased PA, delivered either individually or in a group session, with annual visits.	71 (56)	57.4 (6.5)	3.1, 4.1, 6.1	Overall:Total PA time (self-report)
				Intervention	Recieved an intensive intervention involving detailed dietary and exercise guidance, seven face-to-face counseling sessions in the first year, and quarterly follow-ups.	69 (59)	57.7 (6.4)	3.1, 4.1, 8.1	
Safran Naimark J et al. 2015	Israel	RCT	14	Control (CON)	Continued their healthy lifestyle as they understood it.	29 (72)	46.7 (14.2)	None	Overall: Total PA time (self-report)
				Web-based app	Used the eBalance app, which allowed them to monitor dietary intake and PA.	56 (59)	48.5 (11.3)	1.1, 2.3, 5.1, 6.1	

Authors and year	Country	Study design	Intervention duration, weeks	Group	Details	Baseline n (women % in n)	Baseline age (SD) or [95%CI], years	BCT	Measurement (method)
Sato J et al. 2015	Japan	RCT	8	Control (CON)	No details.	25 (80)	19.1 (1.5) in all participants.	None	Overall: Walking step (device) Total PA and exercise EE (device)
				Intervention	Received three health education sessions, including lectures on diabetes, complication photos, and a patient's experience with diabetic retinopathy and stroke.	25 (80)		2.3, 5.1, 5.2	
Schuna JM Jr et al. 2014	United States	RCT	12	Control (CON)	Continued usual working conditions.	20 (95)	40.3 (10.9)	None	Overall: LPA, MVPA, sedentary time (device)
				Intervention	Used treadmill desks to replace traditional seated office work with light-intensity non-exercise PA throughout the workday, utilizing 8 treadmill desks and computer access.	21 (100)	40.0 (9.5)	3.2, 7.1, 12.1	Work: LPA, MVPA, sedentary time (device)
Semrau J et al. 2021	Germany	RCT	48	BMR+SET (CON)	Group sessions focused on improving physical fitness through education on CLBP, resistance training, water therapy, walking, and cycling.	162 (82)	51 (7.4)	1.1, 1.2, 1.4, 2.2, 3.1, 4.1, 6.1, 8.1, 8.3	Overall: Total PA and exercise time (self-report)
				BMR + BET	Similar to BMR+SET, but focused on but with added focus on self-management strategies, health education, and behavior techniques in a biopsychosocial framework.	164 (76)	51.24 (7.4)	1.1, 1.2, 1.4, 3.1, 4.1, 6.1, 8.3	Leisure: Total PA time (self-report)

Authors and year	Country	Study design	Intervention duration, weeks	Group	Details	Baseline n (women % in n)	Baseline age (SD) or [95%CI], years	BCT	Measurement (method)
Sjöros T et al. 2023	Finland	RCT	4	CONT (CON)	Maintained habitual physical activity and sedentary behavior, with support calls and a research center visit.	21 (52)	56.3 (7.1)	3.2	Overall: Walking step (device)
				INT	Reduced daily sedentary behavior by 1 hour compared to baseline using accelerometers and a mobile app for self-monitoring, with an individual counseling session and support calls.	23 (61)	59.9 (6.0)	1.1, 2.3, 3.2	
Staten LK et al. 2004	United States	RCT	48	PC (CON)	Provider counseling with prescriptions to increase fruit/vegetable consumption and PA.	77 (100)	56.7 (4.9)	3.1, 4.1, 5.1	Overall: MVPA time (self-report)
				PC+HE	PC + referral to two education classes and a monthly health newsletter	73 (100)	58 (4.7)	3.1, 4.1, 5.1	
				PC+HE+CHW	PC + HE + regular support from community health workers.	67 (100)	57 (4.8)	3.1, 4.1, 5.1	
Tosta Maciel RRB et al. 2021	Brazil	cluster-RCT	24	Control (CON)	Received udiovisual content.	148 (69)	41 (8.8)	5.1	Work:VPA, MPA, walking EE (self-

Authors and year	Country	Study design	Intervention duration, weeks	Group	Details	Baseline n (women % in n)	Baseline age (SD) or [95%CI], years	BCT	Measurement (method)
				Intervention	Received udiovisual content and tutor support.	178 (66)	40 (9.1)	3.2, 5.1	report)Leisure: VPA, MPA, walking EE (self-report)
Tully MA et al. 2007	Northern Ireland	RCT	12	Control (CON)	Maintained their current lifestyle.	44 (52)	49.1 (6.3)	None	Overall: Walking distance (device)
				3-day	Walk briskly for 30 minutes, 3 days per week, using a pedometer and diary to record steps, duration, breathlessness, and difficulties.	42 (62)	47.8 (6.0)	2.2, 2.3, 4.1	
				5-day	Same as 3-day group, but walk 5 days per week.	20 (75)	46.4 (4.8)	2.2, 2.3, 4.1	
Turner-McGrievy G et al. 2011	United States	RCT	24	Podcast (CON)	Received 2 weekly podcasts for 3 months, then 2 minipodcasts for 3 months.	49 (73)	43.2 (11.7)	5.1	Overall: Total PA EE (self-report)
				Podcast+Mobile	Podcast + a diet and activity app and a Twitter account. Engaged in Twitter groups for interaction and support, with daily coordinator messages.	47 (77)	42.6 (10.7)	2.3, 3.1, 4.1, 5.1	

Authors and year	Country	Study design	Intervention duration, weeks	Group	Details	Baseline n (women % in n)	Baseline age (SD) or [95%CI], years	BCT	Measurement (method)
Unick JL et al. 2017	United States	RCT	16	Control (CON)	Attended one group session with general info; chose SC or LC strategies, and received newsletters and online resources.	595 (78) in all participants	27.7 (4.4) in all participants	5.1	Overall: Walking step (device)
				Small Change	Daily small dietary and activity changes; reduced calorie intake by 100 cal/day, increased steps by 2000/day. Monitored and received feedback.			1.1, 2.2, 2.3	
				Big Change	Major diet and activity changes; reduced calorie intake by 500-1000 cal/day, increased PA to ≥250 minutes/week. Monitored and received feedback.			1.1, 2.2, 2.3	
Wang JB et al. 2015	United States	RCT	6	Comparison (CON)	Used Fitbit One for self-monitoring without SMS prompts.	34 (18)	47.1 (11.9)	1.3, 2.3, 4.1	Overall: Walking step (device) MVPA and total PA time (device)
				Intervention	Received daily SMS prompts to engage in PA based on three preferred times, with 42 messages.	33 (36)	49.3 (11.5)	1.3, 2.3, 4.1, 7.1	
Webber KH et al. 2016	United States	RCT	20	EBT (CON)	10 weekly 90-minute group sessions focused on stress management skills, sleep, exercise, and whole foods diet, with follow-up emails during weeks 11-20.	24 (83)	45.5 (8.0)	1.1, 4.1, 4.2, 8.1, 8.3	Overall: Total PA time (device)
				BWL	10 weekly 90-minute group sessions based on DPP, focusing on self-monitoring of diet and physical activity, with follow-up emails during weeks 11-20.	25 (84)	44.0 (8.8)	1.1, 1.2, 1.3, 2.3, 3.1, 4.1, 6.1, 8.1, 8.3	

Authors and year	Country	Study design	Intervention duration, weeks	Group	Details	Baseline n (women % in n)	Baseline age (SD) or [95%CI], years	BCT	Measurement (method)
Weinhold KR et al. 2015	United States	RCT	16	Control (CON)	Received usual care from their health care providers and got a weight loss booklet.	34 (79)	51.0 (8.1)	1.3, 3.1, 5.1	Overall: MVPA time (device)
				Intervention	16-week DPP-based program with weekly 60-minute group sessions, focusing on a low-fat diet, 7% weight loss, and 150 minutes of moderate to vigorous PA weekly.	35 (80)	51.6 (9.5)	1.1, 1.3, 1.4, 1.5, 2.2, 2.3, 3.1, 4.1, 5.1, 6.1, 12.1	
Willms A et al. 2023	Canada	RCT	8	CON (CON)	Received weekly educational emails on PA and heart health.	18 (94)	55.1 (6.43)	5.1	Overall: MVPA time (device) Walking step (device)
				PPM	Participated in a structured 8-week Healthy Hearts program based on the M-PAC framework, with CAD $0.02 per minute of MVPA tracked via Fitbit, earning up to CAD $20.	19 (84)	55.4 (5.70)	1.1, 1.2, 1.4, 2.3, 4.1, 8.7, 10.1, 10.2	
				SFII	Similar to PPM, but signed a mock contract to "invest" CAD $400, earning up to 5% (CAD $20) based on PA adherence.	18 (94)	55.8 (6.17)	1.1, 1.2, 1.4, 2.3, 4.1, 10.1, 10.2	
Yamauchi T et al. 2013	Japan	RCT	10	Control (CON)	One health seminar after 4 weeks.	19 (53)	20.7 (3.3)	5.1	Overall: Walking step (device) LPA, MPA

Authors and year	Country	Study design	Intervention duration, weeks	Group	Details	Baseline n (women % in n)	Baseline age (SD) or [95%CI], years	BCT	Measurement (method)
				Intervention	10-week daily non-exercise PA program with weekly individual counseling, three-axis PA monitor use, self-set step targets, three bottles of catechin tea weekly, and two 90-min health seminars at weeks 4 and 8.	23 (52)	19.4 (2.5)	1.3, 5.1, 10.2	and VPA time (device)

Note: Months of intervention duration were transformed into weeks by multiplying four. RCT, randomised controlled trial; n, sample size; SD, standard deviation; CI, confidence interval; BCT, behaviour change technique; EE, energy expenditure; PA, physical activity; LPA, light physical activity; MPA, moderate physical activity; VPA, vigorous physical activity; MVPA, moderate and vigorous physical activity; CON, group treated as control group in the meta-analysis and meta-regression.
