# Supplementary material for: Effectiveness and Components of Health Behavior Interventions on Increasing Physical Activity Among Healthy Young and Middle-Aged Adults: A Systematic Review with Meta-Analyses
Source: Behav Sci (Basel). 2024 Dec 19;14(12):1224. doi: 10.3390/bs14121224 (PMC11673272; doi:10.3390/bs14121224)
Supplement: Supplementary file 1 [file behavsci-14-01224-s001.zip › S3_Sup_biasAY.pdf]

Supplementary Table S5. Risk of bias in each included study.

Authors and year	Random sequence generation	Allocation concealment	Blinding of participants and personnel	Blinding of outcome assessment	Incomplete outcome data	Selective reporting	Other bias	Overall bias
Aadahl M et al. 2014	0	0	-1	-2	-1	-1	0	-1
Aldana SG et al. 2005	0	0	-1	-1	0	-1	0	0
Allen A et al. 2018	-1	-2	-2	-2	-1	-1	0	-2
Allen JK et al. 2013	-1	-1	-1	-1	0	-1	0	-1
Allman-Farinelli M et al. 2016	0	0	-1	0	0	0	0	0
Almhdawi KA et al. 2020	0	0	-1	-1	0	0	0	0
Alsaleh E 2023	0	0	-1	0	0	-1	0	0
An LC et al. 2013	0	0	-1	-1	0	-1	0	0
Andersen LL et al. 2013	0	0	-1	0	0	0	0	0
Annesi JJ 2012	-2	-2	-2	-2	0	-2	0	-2
Annesi JJ et al. 2015	0	-1	-1	-1	-1	-1	0	-1
Appel LJ et al. 2003	0	0	0	0	0	0	0	0
Ashton LM et al. 2017	-1	-1	0	0	0	0	0	0
Assunção MC et al. 2010	0	0	-1	-2	-1	-1	0	-1
Ball K et al. 2005	0	-1	-1	-1	-1	-2	0	-1
Beleigoli A et al. 2020	0	0	-1	0	-1	-1	0	0
Benito PJ et al. 2020	0	0	-1	-1	-1	0	0	0
Bergman F et al. 2018	0	0	-2	-1	0	0	0	0
Biddle SJ et al. 2015	0	-1	-2	-2	0	0	0	-1
Blake H et al. 2019	-1	-1	-2	-1	-2	0	0	-2

Authors and year	Random sequence generation	Allocation concealment	Blinding of participants and personnel	Blinding of outcome assessment	Incomplete outcome data	Selective reporting	Other bias	Overall bias
Brakenridge CL et al. 2016	0	-1	0	0	0	0	-2	0
Cho AR et al. 2019	0	0	-1	0	0	-1	0	0
Cleo G et al. 2019	0	0	-1	0	0	0	0	0
Compton SE et al. 2022	0	-1	-1	-1	-2	-1	0	-1
Conroy MB et al. 2015	0	-1	-1	-1	-2	-2	0	-2
Creasy SA et al. 2022	0	-1	-1	-1	0	0	0	0
Daly RM et al. 2020	0	0	0	0	-1	-1	0	0
Davy BM et al. 2017	0	0	0	-1	0	-1	0	0
Dorling JL et al. 2021	0	0	0	0	-1	-1	0	0
Duggan C et al. 2014	0	-1	-1	0	-2	-2	0	-1
Dunn AL et al. 1998	0	0	-1	-1	-2	-1	0	-1
Dunn AL et al. 1999	-1	-1	-1	0	-1	0	0	0
Eaglehouse YL et al. 2016	0	-1	-1	0	0	0	0	0
Edwardson CL et al. 2018	0	-1	-1	-1	-1	-1	0	-1
Finkelstein EA et al. 2016	0	-1	-1	-1	-2	-2	0	-2
Fischer X et al. 2019	0	-1	-1	-1	-1	-1	0	-1
Fukuoka Y et al. 2015	0	-1	-1	-1	-1	-1	0	-1
Furukawa F et al. 2003	-1	-1	-1	-1	-2	-1	0	-2
Genin PM et al. 2017	-1	0	-1	-1	0	-1	0	0
Gill DP et al. 2019	0	0	-1	-1	0	0	0	0
Gomersall SR et al. 2015	0	-1	-1	-1	-1	-1	0	-1

Authors and year	Random sequence generation	Allocation concealment	Blinding of participants and personnel	Blinding of outcome assessment	Incomplete outcome data	Selective reporting	Other bias	Overall bias
Gorin AA et al. 2013	0	-1	-1	-1	0	-1	0	0
Grey EB et al. 2019	0	-1	-1	-1	0	0	0	0
Harrington DM et al. 2014	0	-1	-1	-1	-1	-1	0	-1
Healy GN et al. 2016	0	0	-2	-2	-1	0	0	-1
Hivert MF et al. 2007	0	-1	-1	-1	-1	-1	0	-1
Hunter JR et al. 2008	-1	-1	-1	-1	-2	0	0	-1
Hurling R et al. 2007	-1	-1	-1	-1	0	-1	0	-1
Jakicic JM et al. 2015	0	-1	-1	-1	-2	-1	0	-1
Jakicic JM et al. 2016	0	-1	-1	-1	-1	0	0	0
Jakicic JM et al. 2022	0	-1	-1	-1	-1	0	0	0
Jamal SN et al. 2016	0	0	-2	-2	0	0	0	0
Johnston V et al. 2019	0	0	-1	-1	0	-1	0	0
Juul L et al. 2016	0	0	-1	-1	-1	0	0	0
Kanaya AM et al. 2012	0	-1	-2	-2	0	-1	0	-1
Kim JY et al. 2015	0	0	-1	-1	0	-1	0	0
Kleist B et al. 2017	-1	-1	-1	-1	-1	-1	0	-1
Kolt GS et al. 2017	0	-1	-1	-1	0	0	0	0
Lewis E et al. 2019	0	-1	-1	-1	0	-1	0	0
Lim SL et al. 2022	0	0	-2	-2	0	0	0	0
Liukkonen M et al. 2017	0	-1	-1	-1	-2	-1	0	-1
Lombard C et al. 2010	0	-1	-1	0	-1	0	0	0

Authors and year	Random sequence generation	Allocation concealment	Blinding of participants and personnel	Blinding of outcome assessment	Incomplete outcome data	Selective reporting	Other bias	Overall bias
Looyestyn J et al. 2018	0	0	-1	-1	0	0	0	0
Low V et al. 2015	0	-2	-2	-2	-1	0	0	-2
Lugones-Sanchez C et al. 2022	0	0	-1	-1	-2	0	0	0
Maddison R et al. 2014	0	0	-1	-1	0	-1	0	0
Madjd A et al. 2019	0	-1	-1	-1	0	-1	0	0
Maher C et al. 2015	0	0	0	0	0	0	0	0
Mansi S et al. 2015	0	0	-2	0	0	0	0	0
Marston KJ et al. 2019	0	-1	-2	0	0	0	0	0
Martin CK et al. 2019	0	0	-2	0	-1	0	0	0
Maruyama C et al. 2010	0	-1	-2	-1	-1	-1	0	-1
Maylor BD et al. 2018	0	-1	-2	-1	-1	-1	0	-1
McRobbie H et al. 2016	0	-1	-1	0	-1	0	0	0
Merom D et al. 2007	0	-1	-2	0	-1	-1	0	-1
Meyer JD et al. 2018	0	-1	-2	-1	0	0	0	0
Miller CK et al. 2015	0	0	-2	-1	0	-1	0	0
Monroe CM et al. 2023	0	-1	-1	-1	0	0	0	0
Morgan PJ et al. 2011	0	0	-2	0	0	-1	0	0
Mueller J et al. 2022	0	0	-1	-1	-1	0	0	0
Nakata Y et al. 2022	0	0	-1	0	0	0	0	0
Newton RL Jr et al. 2004	0	-1	-2	-1	-2	-1	-2	-2
Nishimura M et al. 2019	0	0	-2	-1	0	0	0	0

Authors and year	Random sequence generation	Allocation concealment	Blinding of participants and personnel	Blinding of outcome assessment	Incomplete outcome data	Selective reporting	Other bias	Overall bias
Obling KH et al. 2019	0	0	-2	0	-1	0	0	0
Oftedal S et al. 2019	0	0	-2	-1	-1	0	0	0
Østbye T et al. 2015	0	-1	-2	-1	-1	0	0	-1
Park KS et al. 2024	0	0	-2	-1	0	0	0	0
Pereira MA et al. 2020	0	0	-1	-1	0	0	0	0
Pesola AJ et al. 2017	0	-1	-2	-1	0	0	0	0
Peterson TR et al. 1999	-1	-1	-2	-1	-1	-1	0	-2
Phaswana M et al. 2023	-1	-1	-1	-1	-1	0	0	-1
Plotnikoff RC et al. 2023	0	-1	-1	-1	-1	0	-1	-1
Poirier J et al. 2016	0	-1	-2	-1	0	-1	-1	-1
Puig-Ribera A et al. 2008	0	-1	-2	-1	-1	-1	0	-1
Puig-Ribera A et al. 2015	0	-1	-2	-1	-1	-1	0	-1
Ribeiro MA et al. 2014	0	0	-1	-1	-2	-1	0	-1
Richardson CR et al. 2010	0	-1	-1	-1	-1	0	0	0
Richardson CR et al. 2016	0	-1	-1	-1	-1	0	0	0
Rosas LG et al. 2020	0	-1	0	-1	0	0	0	0
Rovniak LS et al. 2005	0	-1	-1	-1	0	-1	0	0
Ruusunen A et al. 2012	0	-1	0	-1	0	0	0	0
Safran Naimark J et al. 2015	0	-1	0	-1	0	0	0	0
Sato J et al. 2015	-1	-2	-2	-1	0	-1	-1	-2
Schuna JM Jr et al. 2014	0	-1	-1	-1	0	0	-1	0

Authors and year	Random sequence generation	Allocation concealment	Blinding of participants and personnel	Blinding of outcome assessment	Incomplete outcome data	Selective reporting	Other bias	Overall bias
Semrau J et al. 2021	-1	0	-1	0	-2	0	0	0
Sjöros T et al. 2023	0	-1	-1	-1	-2	0	0	-1
Staten LK et al. 2004	0	-1	-1	-1	0	-1	0	0
Tosta Maciel RRB et al. 2021	0	-1	-1	-1	0	-1	0	0
Tully MA et al. 2007	0	-1	-1	-1	0	-1	0	0
Turner-McGrievy G et al. 2011	0	0	0	0	0	0	0	0
Unick JL et al. 2017	0	-1	-1	-1	-1	-1	0	-1
Wang JB et al. 2015	0	0	-1	-1	0	-1	0	0
Webber KH et al. 2016	0	-1	-1	0	0	0	0	0
Weinhold KR et al. 2015	0	-1	0	0	0	-1	0	0
Willms A et al. 2023	0	-1	-1	-1	0	-1	0	0
Yamauchi T et al. 2013	0	-1	-1	-1	0	-1	0	0

Note: 0, no risk with showing by green cells; -1, low risk with showing by yellow cells; -2, high risk with showing by red cells.
