# Supplementary material for: Effectiveness and Components of Health Behavior Interventions on Increasing Physical Activity Among Healthy Young and Middle-Aged Adults: A Systematic Review with Meta-Analyses
Source: Behav Sci (Basel). 2024 Dec 19;14(12):1224. doi: 10.3390/bs14121224 (PMC11673272; doi:10.3390/bs14121224)
Supplement: Supplementary file 1 [file behavsci-14-01224-s001.zip › S4_Sup_forest.pdf]

Study	Intervention Total	Intervention Mean	Intervention SD	Control Total	Control Mean	Control SD	Outcome	Unit	Domain	SMD [95%-CI]	Weight	P-value
Madjd A et al. 2019	33	2196.0	789.0	32	2965.0	795.0	Walking	Step	Overall	-1.0 [-1.5; -0.4]	1.0%	< 0.001
Oftedal S et al. 2019	14	-232.0	429.5	20	75.0	467.9	MVPA	Time	Overall	-0.7 [-1.4; 0.0]	0.9%	0.065
Marston KJ et al. 2019	29	-490.1	323.4	15	-342.9	271.9	Total PA	EE	Overall	-0.5 [-1.1; 0.2]	0.9%	0.144
Wang JB et al. 2015	30	-1.1	13.2	29	4.3	10.8	MVPA	Time	Overall	-0.4 [-1.0; 0.1]	0.9%	0.093
Almhdawi KA et al. 2020	21	822.1	3005.3	20	2458.0	4186.5	Total PA	EE	Overall	-0.4 [-1.1; 0.2]	0.9%	0.163
Bergman F et al. 2018	37	-8.0	13.5	38	-2.0	13.7	MVPA	Time	Overall	-0.4 [-0.9; 0.0]	1.0%	0.062
Allen JK et al. 2015	41	-3.0	5.5	35	-0.6	6.2	MVPA	Time	Overall	-0.4 [-0.9; 0.1]	1.0%	0.084
Biddle SJ et al. 2015	37	-15.8	73.4	54	6.5	52.8	MVPA	Time	Overall	-0.4 [-0.8; 0.1]	1.0%	0.098
Ostbye T et al. 2015	140	0.0	0.4	144	0.2	0.4	MVPA	Time	Overall	-0.3 [-0.5; -0.1]	1.0%	0.008
Monroe CM et al. 2023	49	2.9	10.4	50	6.0	17.6	MVPA	Time	Overall	-0.2 [-0.6; 0.2]	1.0%	0.286
Plotnikoff RC et al. 2023	79	114.2	782.4	84	267.9	694.9	MVPA	EE	Overall	-0.2 [-0.5; 0.1]	1.0%	0.188
Richardson CR et al. 2016	159	3.4	19.3	81	6.9	19.7	MVPA	Time	Overall	-0.2 [-0.4; 0.1]	1.0%	0.189
Dunn AL et al. 1998	114	1.3	1.1	121	1.5	1.1	Total PA	EE	Overall	-0.2 [-0.4; 0.1]	1.0%	0.174
Nishimura M et al. 2019	13	3.9	12.5	13	5.9	12.6	Walking	Time	Overall	-0.2 [-0.9; 0.6]	0.8%	0.694
Phaswana M et al. 2023	44	3.4	20.9	18	6.4	25.3	MVPA	Time	Overall	-0.1 [-0.7; 0.4]	0.9%	0.633
Brakenridge CL et al. 2016	66	-259.1	1543.9	87	-42.3	1774.1	Walking	Step	Overall	-0.1 [-0.4; 0.2]	1.0%	0.432
Lugones-Sanchez C et al. 2022	216	39.9	130.6	227	58.0	158.4	MVPA	Time	Overall	-0.1 [-0.3; 0.1]	1.1%	0.191
Lombard C et al. 2010	79	-623.0	5232.4	80	-188.0	6205.6	Walking	Step	Overall	-0.1 [-0.4; 0.2]	1.0%	0.635
Dunn AL et al. 1999	90	0.7	2.1	98	0.8	2.1	Total PA	EE	Overall	-0.1 [-0.4; 0.2]	1.0%	0.622
Jamal SN et al. 2016	97	-18.9	2329.2	97	90.3	967.2	Total PA	EE	Overall	-0.1 [-0.3; 0.2]	1.0%	0.671
Jakicic JM et al. 2016	237	14.5	1825.4	233	124.0	1814.6	MVPA	EE	Overall	-0.1 [-0.2; 0.1]	1.1%	0.515
Blake H et al. 2019	196	5.8	28.0	86	7.4	33.8	Total PA	Time	Overall	-0.1 [-0.3; 0.2]	1.0%	0.678
Turner-McGrievy G et al. 2011	47	86.8	182.1	49	96.7	185.5	Total PA	EE	Overall	-0.1 [-0.5; 0.3]	1.0%	0.794
Unick JL et al. 2017	359	199.3	2696.9	191	342.0	2707.7	Walking	Step	Overall	-0.1 [-0.2; 0.1]	1.1%	0.556
Ruusunen A et al. 2012	69	56.3	381.2	71	66.7	461.6	Total PA	Time	Overall	-0.0 [-0.4; 0.3]	1.0%	0.885
Ball K et al. 2005	29	555.0	983.0	34	569.0	849.0	MVPA	EE	Overall	-0.0 [-0.5; 0.5]	1.0%	0.952
Edwardson CL et al. 2018	56	0.5	27.9	39	0.9	26.1	MVPA	Time	Overall	-0.0 [-0.4; 0.4]	1.0%	0.943
Pesola AJ et al. 2017	53	-2.8	17.6	53	-2.6	17.6	MVPA	Time	Overall	-0.0 [-0.4; 0.4]	1.0%	0.954
Kolt GS et al. 2017	144	4.4	21.7	78	4.5	20.4	MVPA	Time	Overall	-0.0 [-0.3; 0.3]	1.0%	0.979
Schuna JM Jr et al. 2014	15	0.1	0.6	16	0.1	0.7	MVPA	Time	Overall	0.0 [-0.7; 0.7]	0.9%	1.000
Merom D et al. 2007	207	48.9	206.4	107	47.0	76.5	MVPA	Time	Overall	0.0 [-0.2; 0.2]	1.0%	0.925
Gill DP et al. 2019	59	2.1	25.8	59	1.4	25.7	Total PA	EE	Overall	0.0 [-0.3; 0.4]	1.0%	0.873
McRobbie H et al. 2016	194	359.0	3793.0	97	215.0	5834.9	Total PA	EE	Overall	0.0 [-0.2; 0.3]	1.0%	0.801
Finkelstein EA et al. 2016	554	6.1	709.6	189	-16.0	45.6	MVPA	Frequency	Overall	0.0 [-0.1; 0.2]	1.1%	0.670
Richardson CR et al. 2010	254	2494.0	2525.0	70	2402.0	2232.0	Walking	Step	Overall	0.0 [-0.2; 0.3]	1.0%	0.783
Liukkonen M et al. 2017	79	48.6	3146.8	42	-89.0	3265.2	Total PA	EE	Overall	0.0 [-0.3; 0.4]	1.0%	0.822
Healy GN et al. 2016	121	1.5	14.7	86	0.6	27.3	Walking	Time	Overall	0.0 [-0.2; 0.3]	1.0%	0.761
Juul L et al. 2016	63	233.0	5872.6	64	-114.0	7550.3	Total PA	EE	Overall	0.1 [-0.3; 0.4]	1.0%	0.774
Semrau J et al. 2021	142	0.3	12.3	131	-0.3	12.4	Total PA	Time	Overall	0.1 [-0.2; 0.3]	1.0%	0.666
Maddison R et al. 2014	405	364.5	3083.1	432	207.9	2629.2	Walking	EE	Overall	0.1 [-0.1; 0.2]	1.1%	0.429
Appel LJ et al. 2003	462	0.5	2.7	239	0.3	2.9	Total PA	EE	Overall	0.1 [-0.1; 0.2]	1.1%	0.361
Davy BM et al. 2017	79	0.6	19.6	80	-1.1	18.9	Total PA	EE	Overall	0.1 [-0.2; 0.4]	1.0%	0.594
Staten LK et al. 2004	138	22.7	78.2	76	15.1	64.1	MVPA	Time	Overall	0.1 [-0.2; 0.4]	1.0%	0.471
Puig-Ribera A et al. 2008	44	44.0	2485.9	26	-191.0	1620.0	Walking	Step	Overall	0.1 [-0.4; 0.6]	1.0%	0.671
Webber KH et al. 2016	22	0.0	65.6	23	-6.0	42.1	Total PA	Time	Overall	0.1 [-0.5; 0.7]	0.9%	0.719
Martin CK et al. 2019	110	-3.3	33.2	61	-7.2	34.6	Total PA	Time	Overall	0.1 [-0.2; 0.4]	1.0%	0.472
Kanaya AM et al. 2012	113	3.0	23.4	117	0.4	21.3	Total PA	EE	Overall	0.1 [-0.1; 0.4]	1.0%	0.373
Maher C et al. 2015	44	97.0	331.7	52	56.0	338.9	MVPA	Time	Overall	0.1 [-0.3; 0.5]	1.0%	0.554
Obling KH et al. 2019	70	1.5	30.6	61	-1.7	18.0	MVPA	Time	Overall	0.1 [-0.2; 0.5]	1.0%	0.477
Rosas LG et al. 2020	92	0.3	5.0	99	-0.4	3.9	Total PA	EE	Overall	0.2 [-0.1; 0.4]	1.0%	0.281
Looyestyn J et al., 2018	32	140.0	282.8	37	91.0	285.9	MVPA	Time	Overall	0.2 [-0.3; 0.6]	1.0%	0.481
Allman-Farinelli M et al. 2016	123	625.0	1932.0	125	302.0	1411.0	Total PA	EE	Overall	0.2 [-0.1; 0.4]	1.0%	0.134
Gorin AA et al/ 2013	204	484.2	1981.9	198	82.7	2183.7	Total PA	EE	Overall	0.2 [-0.0; 0.4]	1.1%	0.054
Nakata Y et al. 2022	68	-2.0	33.3	64	-7.5	20.2	MVPA	Time	Overall	0.2 [-0.1; 0.5]	1.0%	0.259
Mueller J et al. 2022	145	3.0	29.8	179	-4.1	36.5	Total PA	EE	Overall	0.2 [-0.0; 0.4]	1.0%	0.061
Jakicic JM et al. 2015	131	1015.6	1791.6	64	624.3	1697.6	Total PA	EE	Overall	0.2 [-0.1; 0.5]	1.0%	0.148
Genin PM et al. 2017	73	419.0	872.1	22	237.8	330.5	Total PA	EE	Overall	0.2 [-0.2; 0.7]	1.0%	0.347
Pereira MA et al. 2020	263	0.5	2.9	224	-0.2	2.9	MVPA	Time	Overall	0.2 [0.1; 0.4]	1.1%	0.008
Lim SL et al. 2022	72	44.2	144.8	76	11.2	123.4	Total PA	Time	Overall	0.2 [-0.1; 0.6]	1.0%	0.138
Hivert MF et al. 2007	48	-89.0	879.9	48	-292.0	762.1	Total PA	EE	Overall	0.2 [-0.2; 0.6]	1.0%	0.233
Maruyama C et al. 2010	44	445.0	2710.0	22	-204.0	2389.0	Walking	Step	Overall	0.2 [-0.3; 0.8]	1.0%	0.348
Rovniak LS et al. 2005	25	57.1	53.4	25	44.8	43.2	Walking	Time	Overall	0.2 [-0.3; 0.8]	0.9%	0.381
Grey EB et al. 2019	29	12.0	44.0	28	0.0	50.0	MVPA	Time	Overall	0.3 [-0.3; 0.8]	0.9%	0.344
Aadahl M et al. 2014	79	0.1	0.5	66	-0.0	0.6	Walking	Time	Overall	0.3 [-0.1; 0.6]	1.0%	0.129
Daly RM et al. 2020	123	5.3	17.5	121	1.0	15.1	MVPA	Time	Overall	0.3 [0.0; 0.5]	1.0%	0.042
Kim JY et al. 2015	101	692.0	1297.9	95	111.6	2779.9	Total PA	EE	Overall	0.3 [-0.0; 0.6]	1.0%	0.061
Harrington DM et al. 2014	47	8.0	18.7	52	3.0	18.0	MVPA	Time	Overall	0.3 [-0.1; 0.7]	1.0%	0.181
Lewis E et al. 2019	29	15.0	2195.2	32	-494.0	1457.5	Total PA	EE	Overall	0.3 [-0.2; 0.8]	1.0%	0.290
Weinhold KR et al. 2015	35	23.9	71.0	34	2.2	72.3	MVPA	Time	Overall	0.3 [-0.2; 0.8]	1.0%	0.216
Dorling JL et al. 2021	20	2.0	10.7	20	-1.0	8.6	MVPA	Time	Overall	0.3 [-0.3; 0.9]	0.9%	0.340
Fischer X et al. 2019	153	27.4	92.2	60	-5.1	91.5	MVPA	Time	Overall	0.4 [0.1; 0.7]	1.0%	0.022
Miller CK et al. 2015	35	965.0	2190.6	33	145.2	2127.1	Walking	Step	Overall	0.4 [-0.1; 0.9]	1.0%	0.125
Benito PJ et al. 2020	66	1515.5	2029.7	18	724.0	1979.0	Total PA	EE	Overall	0.4 [-0.1; 0.9]	0.9%	0.147
Conroy MB et al. 2015	44	7.5	15.9	31	1.9	10.5	Total PA	EE	Overall	0.4 [-0.1; 0.9]	1.0%	0.094
Morgan PJ et al. 2011	65	0.4	0.6	45	0.1	0.7	Total PA	EE	Overall	0.5 [0.1; 0.9]	1.0%	0.017
Sato J et al. 2015	25	121.4	200.5	25	35.9	154.3	Total PA	EE	Overall	0.5 [-0.1; 1.0]	0.9%	0.101
Poirier J et al. 2016	107	309.0	1874.0	110	-661.0	1824.0	Walking	Step	Overall	0.5 [0.3; 0.8]	1.0%	< 0.001
Willms A et al. 2023	38	84.9	215.4	18	-20.7	134.2	MVPA	Time	Overall	0.5 [-0.0; 1.1]	0.9%	0.064
Tully MA et al. 2007	75	74.8	105.1	18	18.0	74.6	Walking	Distance	Overall	0.6 [0.0; 1.1]	0.9%	0.034
Safran Naimark J et al. 2015	56	63.0	155.7	29	-30.0	148.1	Total PA	Time	Overall	0.6 [0.1; 1.1]	1.0%	0.010
Aldana SG et al. 2005	167	12080.0	16909.0	170	2057.0	15936.0	Walking	Step	Overall	0.6 [0.4; 0.8]	1.0%	< 0.001
Ashton LM et al. 2017	26	154.1	205.6	24	26.1	199.2	MVPA	Time	Overall	0.6 [0.1; 1.2]	0.9%	0.032
Sjoros T et al. 2023	22	3200.0	2336.6	19	1700.0	2292.6	Walking	Step	Overall	0.6 [0.0; 1.3]	0.9%	0.048
Meyer JD et al. 2018	32	-6.4	10.8	17	-17.9	25.7	MVPA	Time	Overall	0.6 [0.0; 1.3]	0.9%	0.035
Hunter JR et al. 2008	22	720.0	595.0	24	407.0	246.0	Total PA	EE	Overall	0.7 [0.1; 1.3]	0.9%	0.024
Gomersall SR et al. 2015	74	35.5	72.0	37	-13.0	57.0	MVPA	EE	Overall	0.7 [0.3; 1.1]	1.0%	< 0.001
Furukawa F et al. 2003	24	2.3	2.2	25	0.9	1.3	Total PA	EE	Overall	0.8 [0.2; 1.3]	0.9%	0.010
Maylor BD et al. 2018	38	1212.0	1481.6	30	52.0	1456.9	Walking	Step	Overall	0.8 [0.3; 1.3]	1.0%	0.002
Fukuoka Y et al. 2015	30	2551.0	4712.0	31	-734.0	3308.0	Walking	Step	Overall	0.8 [0.3; 1.3]	0.9%	0.003
Ribeiro MA et al. 2014	106	743.6	1453.9	45	-597.0	1987.1	Walking	Step	Overall	0.8 [0.5; 1.2]	1.0%	< 0.001
Allen A et al. 2018	21	2977.3	6025.4	11	-2767.0	6875.4	Total PA	EE	Overall	0.9 [0.1; 1.7]	0.8%	0.023
Kleist B et al. 2017	38	4.6	4.7	44	0.5	4.1	Total PA	EE	Overall	0.9 [0.5; 1.4]	1.0%	< 0.001
Compton SE et al. 2022	48	5020.7	1777.5	31	2912.0	1842.0	Walking	Step	Overall	1.2 [0.7; 1.6]	1.0%	< 0.001
Yamauchi T et al. 2013	23	2674.0	1578.0	19	269.0	1782.0	Walking	Step	Overall	1.4 [0.7; 2.1]	0.9%	< 0.001
Alsaleh E 2023	68	503.0	325.2	63	6.0	271.2	MVPA	EE	Overall	1.6 [1.2; 2.0]	1.0%	< 0.001
Mansi S et al. 2015	29	903.0	579.7	29	8.0	404.9	Total PA	EE	Overall	1.8 [1.2; 2.4]	0.9%	< 0.001
Jakicic JM et al. 2022	208	807.9	400.7	109	89.3	398.6	Total PA	EE	Overall	1.8 [1.5; 2.1]	1.0%	< 0.001
Hurling R et al. 2007	47	12.0	3.1	30	4.0	4.1	Total PA	EE	Overall	2.2 [1.7; 2.8]	0.9%	< 0.001
Park KS et al. 2024	38	630.0	326.0	37	34.6	130.0	Total PA	EE	Overall	2.4 [1.8; 3.0]	0.9%	< 0.001
Peterson TR et al. 1999	343	2.8	2.5	185	-3.1	1.3	Total PA	EE	Overall	2.7 [2.5; 2.9]	1.0%	< 0.001
Cho AR et al. 2019	18	1230.3	413.8	5	-543.6	608.6	Total PA	EE	Overall	3.7 [2.2; 5.3]	0.5%	< 0.001
Puig-Ribera A et al. 2015	91	776.0	226.0	107	-656.0	284.0	Walking	Step	Overall	5.5 [4.9; 6.1]	0.9%	< 0.001
**Random effects model**	**9713**			**7315**						**0.3 [0.2; 0.5]**	**100.0%**	**< 0.001**
**Prediction interval**										**[-1.1; 1.8]**		

Heterogeneity: $I^2 = 92.3\%$, $\tau^2 = 0.5497$, $p < 0.0001$

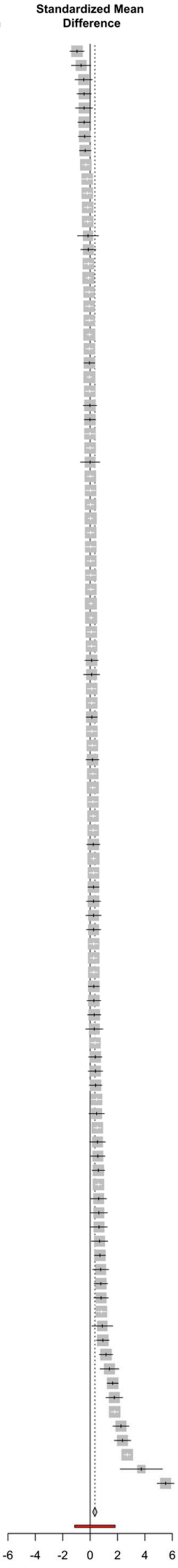

Supplementary Figure S1. Forest plot of primary meta-analysis for physical activity (PA) metrics outcomes. The left side of the plot shows the study authors and publication year, the sample sizes of the intervention and control groups, the mean change, and the standard deviation of the mean change. The right side displays the standardized mean difference (SMD), the 95% confidence interval (95%CI) of the SMD, and the weight of each study. The lower side shows the overall analysis results for the primary meta-analysis from the random-effects model. The I² and τ² are indicators of heterogeneity, and p represents the p-value of the heterogeneity test. MVPA, moderate and vigorous physical activity. PA metrics outcomes were selected in the following order: MVPA > total PA > walking for each study. EE (energy expenditure), e.g., kcal/day, METs hour/day; Time, e.g., hours/day, minutes/day; Step, e.g., steps/day, steps/week; Distance, e.g., m/day; Frequency, e.g., times/week, times/day.

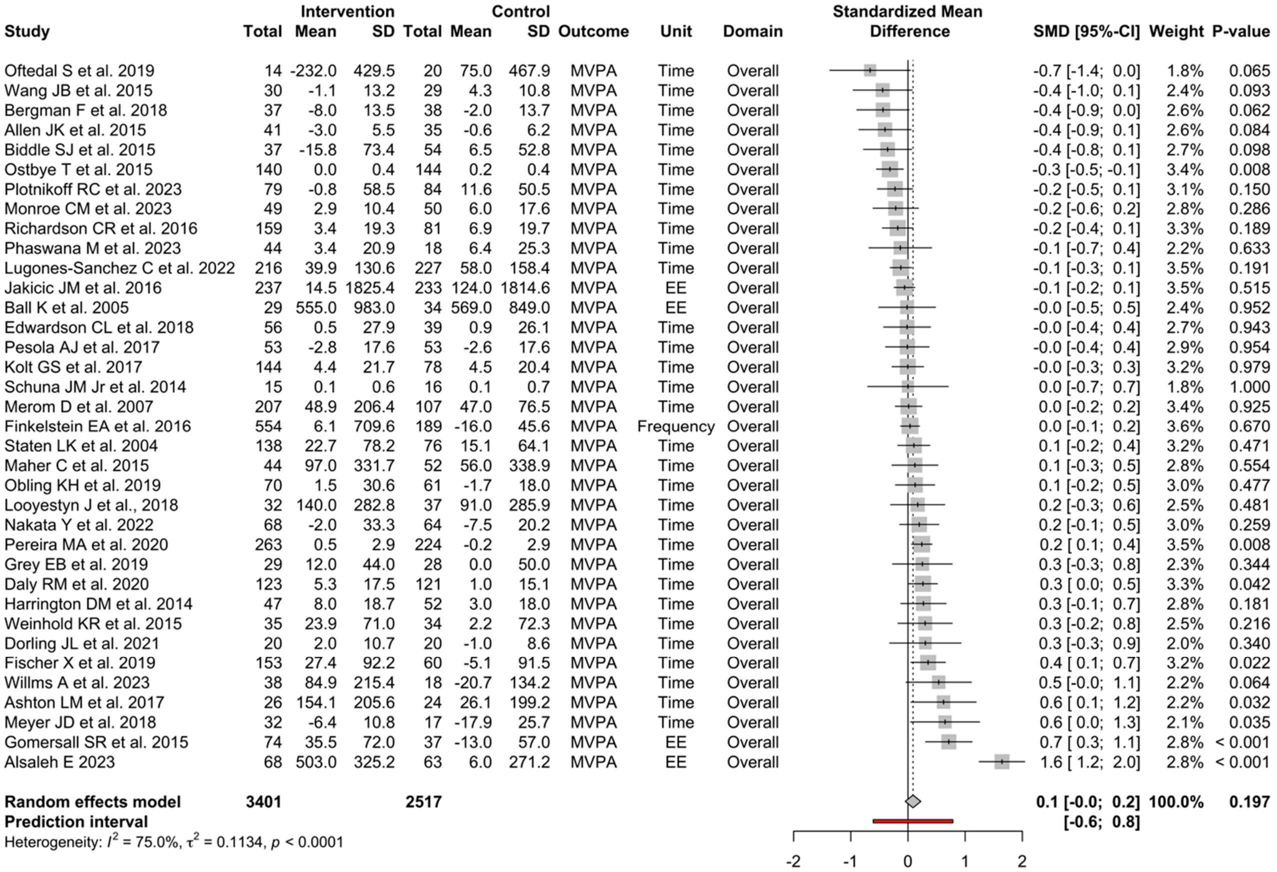

Supplementary Figure S2. Forest plot of subgroup meta-analysis for overall moderate and vigorous physical activity (MVPA). The left side of the plot shows the study authors and publication year, the sample sizes of the intervention and control groups, the mean change, and the standard deviation of the mean change, with units and domain of the extracted data. The right side displays the standardized mean difference (SMD), the 95% confidence interval (95%CI) of the SMD, and the weight of each study. The lower side shows the overall analysis results for main meta-analysis from the random-effects model. The I² and τ² are indicators of heterogeneity, and p represents the P-value of the heterogeneity test. EE (energy expenditure), e.g., kcal/day, METs hour/day; Time, e.g., hours/day, min/day; Frequency, e.g., times/week, times/day.

Study	Intervention Total	Intervention Mean	Intervention SD	Control Total	Control Mean	Control SD	Outcome	Unit	Domain	SMD [95%-CI]	Weight	P-value
Marston KJ et al. 2019	29	-490.1	323.4	15	-342.9	271.9	Total PA	EE	Overall	-0.5 [-1.1; 0.2]	1.9%	0.144
Almhdawi KA et al. 2020	21	822.1	3005.3	20	2458.0	4186.5	Total PA	EE	Overall	-0.4 [-1.1; 0.2]	1.9%	0.163
Wang JB et al. 2015	30	-1.6	24.6	29	7.8	22.6	Total PA	Time	Overall	-0.4 [-0.9; 0.1]	2.0%	0.136
Plotnikoff RC et al. 2023	79	117.0	934.9	84	347.8	832.2	Total PA	EE	Overall	-0.3 [-0.6; 0.0]	2.2%	0.099
Dunn AL et al. 1998	114	1.3	1.1	121	1.5	1.1	Total PA	EE	Overall	-0.2 [-0.4; 0.1]	2.2%	0.174
Merom D et al. 2007	207	82.6	118.2	107	94.0	117.4	Total PA	Time	Overall	-0.1 [-0.3; 0.1]	2.2%	0.419
Jamal SN et al. 2016	97	-18.9	2329.2	97	90.3	967.2	Total PA	EE	Overall	-0.1 [-0.3; 0.2]	2.2%	0.671
Blake H et al. 2019	196	5.8	28.0	86	7.4	33.8	Total PA	Time	Overall	-0.1 [-0.3; 0.2]	2.2%	0.678
Turner-McGrievy G et al. 2011	47	86.8	182.1	49	96.7	185.5	Total PA	EE	Overall	-0.1 [-0.5; 0.3]	2.1%	0.794
Ruusunen A et al. 2012	69	56.3	381.2	71	66.7	461.6	Total PA	Time	Overall	-0.0 [-0.4; 0.3]	2.2%	0.885
Gill DP et al. 2019	59	2.1	25.8	59	1.4	25.7	Total PA	EE	Overall	0.0 [-0.3; 0.4]	2.1%	0.873
McRobbie H et al. 2016	194	359.0	3793.0	97	215.0	5834.9	Total PA	EE	Overall	0.0 [-0.2; 0.3]	2.2%	0.801
Liukkonen M et al. 2017	79	48.6	3146.8	42	-89.0	3265.2	Total PA	EE	Overall	0.0 [-0.3; 0.4]	2.1%	0.822
Juul L et al. 2016	63	233.0	5872.6	64	-114.0	7550.3	Total PA	EE	Overall	0.1 [-0.3; 0.4]	2.2%	0.774
Semrau J et al. 2021	142	0.3	12.3	131	-0.3	12.4	Total PA	Time	Overall	0.1 [-0.2; 0.3]	2.2%	0.666
Appel LJ et al. 2003	462	0.5	2.7	239	0.3	2.9	Total PA	EE	Overall	0.1 [-0.1; 0.2]	2.3%	0.361
Davy BM et al. 2017	79	0.6	19.6	80	-1.1	18.9	Total PA	EE	Overall	0.1 [-0.2; 0.4]	2.2%	0.594
Webber KH et al. 2016	22	0.0	65.6	23	-6.0	42.1	Total PA	Time	Overall	0.1 [-0.5; 0.7]	1.9%	0.719
Martin CK et al. 2019	110	-3.3	33.2	61	-7.2	34.6	Total PA	Time	Overall	0.1 [-0.2; 0.4]	2.2%	0.472
Kanaya AM et al. 2012	113	3.0	23.4	117	0.4	21.3	Total PA	EE	Overall	0.1 [-0.1; 0.4]	2.2%	0.373
Biddle SJ et al. 2015	46	-247.0	2410.9	49	-582.9	2350.3	Total PA	EE	Overall	0.1 [-0.3; 0.5]	2.1%	0.496
Rosas LG et al. 2020	92	0.3	5.0	99	-0.4	3.9	Total PA	EE	Overall	0.2 [-0.1; 0.4]	2.2%	0.281
Allman-Farinelli M et al. 2016	123	625.0	1932.0	125	302.0	1411.0	Total PA	EE	Overall	0.2 [-0.1; 0.4]	2.2%	0.134
Gorin AA et al/ 2013	204	484.2	1981.9	198	82.7	2183.7	Total PA	EE	Overall	0.2 [-0.0; 0.4]	2.3%	0.054
Mueller J et al. 2022	145	3.0	29.8	179	-4.1	36.5	Total PA	EE	Overall	0.2 [-0.0; 0.4]	2.2%	0.061
Jakicic JM et al. 2015	131	1015.6	1791.6	64	624.3	1697.6	Total PA	EE	Overall	0.2 [-0.1; 0.5]	2.2%	0.148
Genin PM et al. 2017	73	419.0	872.1	22	237.8	330.5	Total PA	EE	Overall	0.2 [-0.2; 0.7]	2.0%	0.347
Lim SL et al. 2022	72	44.2	144.8	76	11.2	123.4	Total PA	Time	Overall	0.2 [-0.1; 0.6]	2.2%	0.138
Hivert MF et al. 2007	48	-89.0	879.9	48	-292.0	762.1	Total PA	EE	Overall	0.2 [-0.2; 0.6]	2.1%	0.233
Kim JY et al. 2015	101	692.0	1297.9	95	111.6	2779.9	Total PA	EE	Overall	0.3 [-0.0; 0.6]	2.2%	0.061
Lewis E et al. 2019	29	15.0	2195.2	32	-494.0	1457.5	Total PA	EE	Overall	0.3 [-0.2; 0.8]	2.0%	0.290
Lugones-Sanchez C et al. 2022	216	142.8	179.4	227	79.7	212.8	Total PA	Time	Overall	0.3 [0.1; 0.5]	2.3%	< 0.001
Grey EB et al. 2019	29	67.0	246.0	28	-23.0	264.0	Total PA	EE	Overall	0.3 [-0.2; 0.9]	2.0%	0.192
Benito PJ et al. 2020	66	1515.5	2029.7	18	724.0	1979.0	Total PA	EE	Overall	0.4 [-0.1; 0.9]	2.0%	0.147
Conroy MB et al. 2015	44	7.5	15.9	31	1.9	10.5	Total PA	EE	Overall	0.4 [-0.1; 0.9]	2.1%	0.094
Morgan PJ et al. 2011	65	0.4	0.6	45	0.1	0.7	Total PA	EE	Overall	0.5 [0.1; 0.9]	2.1%	0.017
Sato J et al. 2015	25	121.4	200.5	25	35.9	154.3	Total PA	EE	Overall	0.5 [-0.1; 1.0]	1.9%	0.101
Safran Naimark J et al. 2015	56	63.0	155.7	29	-30.0	148.1	Total PA	Time	Overall	0.6 [0.1; 1.1]	2.1%	0.010
Hunter JR et al. 2008	22	720.0	595.0	24	407.0	246.0	Total PA	EE	Overall	0.7 [0.1; 1.3]	1.9%	0.024
Furukawa F et al. 2003	24	2.3	2.2	25	0.9	1.3	Total PA	EE	Overall	0.8 [0.2; 1.3]	1.9%	0.010
Allen A et al. 2018	21	2977.3	6025.4	11	-2767.0	6875.4	Total PA	EE	Overall	0.9 [0.1; 1.7]	1.7%	0.023
Kleist B et al. 2017	38	4.6	4.7	44	0.5	4.1	Total PA	EE	Overall	0.9 [0.5; 1.4]	2.1%	< 0.001
Gomersall SR et al. 2015	74	0.1	0.2	37	-0.0	0.1	Total PA	EE	Overall	1.0 [0.6; 1.4]	2.1%	< 0.001
Jakicic JM et al. 2022	208	807.9	400.7	109	89.3	398.6	Total PA	EE	Overall	1.8 [1.5; 2.1]	2.2%	< 0.001
Hurling R et al. 2007	47	12.0	3.1	30	4.0	4.1	Total PA	EE	Overall	2.2 [1.7; 2.8]	1.9%	< 0.001
Park KS et al. 2024	38	630.0	326.0	37	34.6	130.0	Total PA	EE	Overall	2.4 [1.8; 3.0]	1.9%	< 0.001
Peterson TR et al. 1999	343	2.8	2.5	185	-3.1	1.3	Total PA	EE	Overall	2.7 [2.5; 2.9]	2.2%	< 0.001
Cho AR et al. 2019	18	1230.3	413.8	5	-543.6	608.6	Total PA	EE	Overall	3.7 [2.2; 5.3]	1.0%	< 0.001
**Random effects model**	**4610**			**3489**						**0.4 [0.2; 0.6]**	**100.0%**	**< 0.001**
**Prediction interval**										**[-1.0; 1.8]**		

Heterogeneity: $I^2 = 93.6\%$, $\tau^2 = 0.4486$, $p < 0.0001$

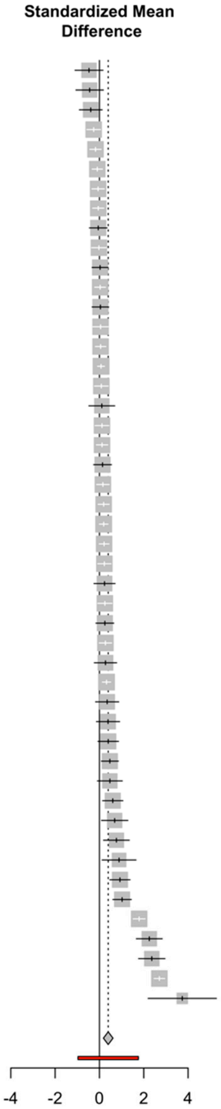

Supplementary Figure S3. Forest plot of subgroup meta-analysis for overall total physical activity (PA). The left side of the plot shows the study authors and publication year, the sample sizes of the intervention and control groups, the mean change, and the standard deviation of the mean change, with units and domain of the extracted data. The right side displays the standardized mean difference (SMD), the 95% confidence interval (95%CI) of the SMD, and the weight of each study. The lower side shows the overall analysis results for main meta-analysis from the random-effects model. The I² and τ² are indicators of heterogeneity, and p represents the p-value of the heterogeneity test. EE (energy expenditure), e.g., kcal/day, METs hour/day; Time, e.g., hours/day, min/day.

Study	Intervention Total	Intervention Mean	Intervention SD	Control Total	Control Mean	Control SD	Outcome	Unit	Domain	SMD [95%-CI]	Weight	P-value
Madjd A et al. 2019	33	2196.0	789.0	32	2965.0	795.0	Walking	Step	Overall	-1.0 [-1.5; -0.4]	2.1%	< 0.001
Monroe CM et al. 2023	49	532.0	1373.0	50	939.0	2068.0	Walking	Step	Overall	-0.2 [-0.6; 0.2]	2.2%	0.255
Biddle SJ et al. 2015	44	-295.8	1714.5	54	-16.0	1489.8	Walking	Step	Overall	-0.2 [-0.6; 0.2]	2.2%	0.392
Nishimura M et al. 2019	13	3.9	12.5	13	5.9	12.6	Walking	Time	Overall	-0.2 [-0.9; 0.6]	1.9%	0.694
Brakenridge CL et al. 2016	66	-259.1	1543.9	87	-42.3	1774.1	Walking	Step	Overall	-0.1 [-0.4; 0.2]	2.2%	0.432
Lombard C et al. 2010	79	-623.0	5232.4	80	-188.0	6205.6	Walking	Step	Overall	-0.1 [-0.4; 0.2]	2.2%	0.635
Willms A et al. 2023	38	-15.0	2434.7	18	183.0	2906.0	Walking	Step	Overall	-0.1 [-0.6; 0.5]	2.1%	0.792
Dunn AL et al. 1998	114	16.5	62.6	121	19.8	57.6	Walking	Time	Overall	-0.1 [-0.3; 0.2]	2.2%	0.677
Unick JL et al. 2017	359	199.3	2696.9	191	342.0	2707.7	Walking	Step	Overall	-0.1 [-0.2; 0.1]	2.3%	0.556
Edwardson CL et al. 2018	62	2.6	23.6	45	3.8	21.9	Walking	Time	Overall	-0.0 [-0.4; 0.3]	2.2%	0.804
Kanaya AM et al. 2012	113	0.4	4.4	117	0.3	0.4	Walking	Time	Overall	0.0 [-0.2; 0.3]	2.2%	0.883
Richardson CR et al. 2010	254	2494.0	2525.0	70	2402.0	2232.0	Walking	Step	Overall	0.0 [-0.2; 0.3]	2.2%	0.783
Healy GN et al. 2016	121	1.5	14.7	86	0.6	27.3	Walking	Time	Overall	0.0 [-0.2; 0.3]	2.2%	0.761
Maddison R et al. 2014	405	364.5	3083.1	432	207.9	2629.2	Walking	EE	Overall	0.1 [-0.1; 0.2]	2.3%	0.429
Maher C et al. 2015	44	38.0	192.4	52	23.0	144.2	Walking	Time	Overall	0.1 [-0.3; 0.5]	2.2%	0.666
Puig-Ribera A et al. 2008	44	44.0	2485.9	26	-191.0	1620.0	Walking	Step	Overall	0.1 [-0.4; 0.6]	2.1%	0.671
Jamal SN et al. 2016	97	63.3	784.0	97	-19.3	431.4	Walking	EE	Overall	0.1 [-0.2; 0.4]	2.2%	0.366
Merom D et al. 2007	207	53.5	56.3	107	43.0	67.3	Walking	Time	Overall	0.2 [-0.1; 0.4]	2.2%	0.146
Dorling JL et al. 2021	20	-83.0	1844.0	20	-422.0	1738.2	Walking	Step	Overall	0.2 [-0.4; 0.8]	2.0%	0.559
Dunn AL et al. 1999	90	26.8	68.7	98	13.1	69.1	Walking	Time	Overall	0.2 [-0.1; 0.5]	2.2%	0.177
Grey EB et al. 2019	29	793.0	3756.0	28	63.0	2307.0	Walking	Step	Overall	0.2 [-0.3; 0.8]	2.1%	0.387
Maruyama C et al. 2010	44	445.0	2710.0	22	-204.0	2389.0	Walking	Step	Overall	0.2 [-0.3; 0.8]	2.1%	0.348
Rovniak LS et al. 2005	25	57.1	53.4	25	44.8	43.2	Walking	Time	Overall	0.2 [-0.3; 0.8]	2.1%	0.381
Aadahl M et al. 2014	79	0.1	0.5	66	-0.0	0.6	Walking	Time	Overall	0.3 [-0.1; 0.6]	2.2%	0.129
Nakata Y et al. 2022	68	-401.0	3613.0	64	-1271.0	2391.0	Walking	Step	Overall	0.3 [-0.1; 0.6]	2.2%	0.109
Wang JB et al. 2015	30	24.0	1511.7	29	-433.0	1195.5	Walking	Step	Overall	0.3 [-0.2; 0.8]	2.1%	0.208
Finkelstein EA et al. 2016	554	55.5	1307.5	189	-480.0	2279.6	Walking	Step	Overall	0.3 [0.2; 0.5]	2.3%	< 0.001
Miller CK et al. 2015	35	965.0	2190.6	33	145.2	2127.1	Walking	Step	Overall	0.4 [-0.1; 0.9]	2.1%	0.125
Ashton LM et al. 2017	26	1588.2	2608.1	24	575.4	2591.4	Walking	Step	Overall	0.4 [-0.2; 0.9]	2.1%	0.180
Sato J et al. 2015	25	436.9	2470.1	25	-460.5	307.7	Walking	Step	Overall	0.5 [-0.1; 1.1]	2.1%	0.081
Poirier J et al. 2016	107	309.0	1874.0	110	-661.0	1824.0	Walking	Step	Overall	0.5 [0.3; 0.8]	2.2%	< 0.001
Tully MA et al. 2007	75	74.8	105.1	18	18.0	74.6	Walking	Distance	Overall	0.6 [0.0; 1.1]	2.1%	0.034
Aldana SG et al. 2005	167	12080.0	16909.0	170	2057.0	15936.0	Walking	Step	Overall	0.6 [0.4; 0.8]	2.2%	< 0.001
Sjoros T et al. 2023	22	3200.0	2336.6	19	1700.0	2292.6	Walking	Step	Overall	0.6 [0.0; 1.3]	2.0%	0.048
Allen A et al. 2018	21	3110.1	12331.9	11	-4222.0	5946.7	Walking	Step	Overall	0.7 [-0.1; 1.4]	1.9%	0.079
Lewis E et al. 2019	29	874.0	2075.6	32	-575.0	2142.6	Walking	Step	Overall	0.7 [0.2; 1.2]	2.1%	0.010
Furukawa F et al. 2003	24	1795.0	1630.0	25	629.0	1372.0	Walking	Step	Overall	0.8 [0.2; 1.3]	2.0%	0.010
Maylor BD et al. 2018	38	1212.0	1481.6	30	52.0	1456.9	Walking	Step	Overall	0.8 [0.3; 1.3]	2.1%	0.002
Fukuoka Y et al. 2015	30	2551.0	4712.0	31	-734.0	3308.0	Walking	Step	Overall	0.8 [0.3; 1.3]	2.1%	0.003
Ribeiro MA et al. 2014	106	743.6	1453.9	45	-597.0	1987.1	Walking	Step	Overall	0.8 [0.5; 1.2]	2.2%	< 0.001
Gill DP et al. 2019	59	1646.0	3302.0	59	-1485.0	3171.5	Walking	Step	Overall	1.0 [0.6; 1.3]	2.2%	< 0.001
Bergman F et al. 2018	37	2174.0	2345.4	38	-298.0	2290.9	Walking	Step	Overall	1.1 [0.6; 1.5]	2.1%	< 0.001
Compton SE et al. 2022	48	5020.7	1777.5	31	2912.0	1842.0	Walking	Step	Overall	1.2 [0.7; 1.6]	2.1%	< 0.001
Yamauchi T et al. 2013	23	2674.0	1578.0	19	269.0	1782.0	Walking	Step	Overall	1.4 [0.7; 2.1]	2.0%	< 0.001
Alsaleh E 2023	68	3000.0	1217.0	63	876.0	1120.2	Walking	Step	Overall	1.8 [1.4; 2.2]	2.2%	< 0.001
Mansi S et al. 2015	29	3799.0	1506.4	29	763.0	1644.4	Walking	Step	Overall	1.9 [1.3; 2.5]	2.0%	< 0.001
Puig-Ribera A et al. 2015	91	776.0	226.0	107	-656.0	284.0	Walking	Step	Overall	5.5 [4.9; 6.1]	2.0%	< 0.001
**Random effects model**	**4141**			**3138**						**0.5 [0.2; 0.7]**	**100.0%**	**< 0.001**
**Prediction interval**										**[-1.3; 2.2]**		

Heterogeneity: $I^2 = 91.4\%$, $\tau^2 = 0.7183$, $p < 0.0001$

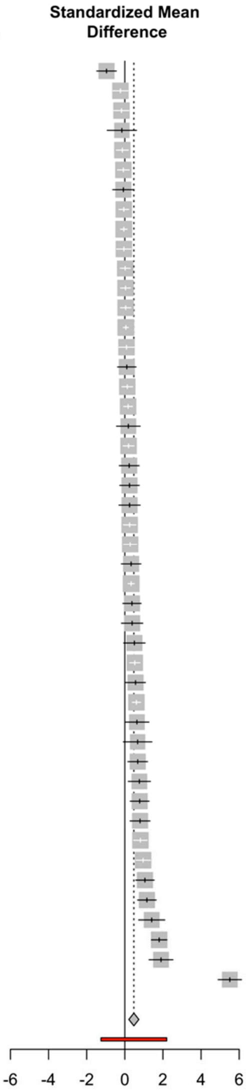

Supplementary Figure S4. Forest plot of subgroup meta-analysis for overall walking. The left side of the plot shows the study authors and publication year, the sample sizes of the intervention and control groups, the mean change, and the standard deviation of the mean change, with units and domain of the extracted data. The right side displays the standardized mean difference (SMD), the 95% confidence interval (95%CI) of the SMD, and the weight of each study. The lower side shows the overall analysis results for main meta-analysis from the random-effects model. The I² and τ² are indicators of heterogeneity, and p represents the p-value of the heterogeneity test. EE (energy expenditure), e.g., kcal/day, METs hour/day; Time, e.g., hours/day, min/day; Step, e.g., steps/day, steps/week; Distance, e.g., m/day.

Study	Intervention Total	Intervention Mean	Intervention SD	Control Total	Control Mean	Control SD	Outcome	Unit	Domain	SMD [95%-CI]	Weight	P-value
Creasy SA et al. 2022	13	-0.9	5.4	18	0.8	3.3	Sedentary	Time	Overall	-0.4 [-1.1; 0.3]	2.9%	0.310
Johnston V et al. 2019	13	82.7	54.3	13	99.3	55.3	Sedentary	Time	Overall	-0.3 [-1.1; 0.5]	2.8%	0.457
Ostbye T et al. 2015	140	0.4	3.1	144	1.3	3.4	Sedentary	Time	Overall	-0.3 [-0.5; -0.1]	3.5%	0.016
McRobbie H et al. 2016	194	0.0	319.8	97	60.0	446.6	Sedentary	Time	Overall	-0.2 [-0.4; 0.1]	3.5%	0.190
Pesola AJ et al. 2017	53	-7.1	70.8	53	4.2	71.5	Sedentary	Time	Overall	-0.2 [-0.5; 0.2]	3.4%	0.417
Dorling JL et al. 2021	20	-48.0	78.0	20	-36.0	72.7	Sedentary	Time	Overall	-0.2 [-0.8; 0.5]	3.1%	0.622
Obling KH et al. 2019	70	-5.5	74.0	61	6.7	88.6	Sedentary	Time	Overall	-0.1 [-0.5; 0.2]	3.4%	0.394
Biddle SJ et al. 2015	34	-0.8	1.3	35	-0.6	1.5	Sedentary	Time	Overall	-0.1 [-0.6; 0.4]	3.3%	0.662
Jakicic JM et al. 2016	237	0.0	3.1	233	0.3	3.1	Sedentary	Time	Overall	-0.1 [-0.3; 0.1]	3.6%	0.300
Brakenridge CL et al. 2016	66	7.4	92.8	87	14.3	72.8	Sedentary	Time	Overall	-0.1 [-0.4; 0.2]	3.5%	0.608
Lombard C et al. 2010	79	-162.3	862.7	80	-85.0	1220.5	Walking	EE	Overall	-0.1 [-0.4; 0.2]	3.5%	0.647
Maylor BD et al. 2018	38	14.7	68.9	30	12.5	68.0	Sedentary	Time	Overall	0.0 [-0.4; 0.5]	3.3%	0.897
Benito PJ et al. 2020	66	1.6	2.7	18	1.4	3.2	Sedentary	Time	Overall	0.1 [-0.5; 0.6]	3.2%	0.814
Dunn AL et al. 1998	114	6.9	17.2	121	5.3	17.3	Sedentary	Time	Overall	0.1 [-0.2; 0.3]	3.5%	0.477
Aadahl M et al. 2014	79	0.3	1.7	66	-0.1	1.7	Sedentary	Time	Overall	0.2 [-0.1; 0.5]	3.5%	0.248
Grey EB et al. 2019	29	24.0	58.0	28	10.0	59.0	Sedentary	Time	Overall	0.2 [-0.3; 0.8]	3.2%	0.375
Gomersall SR et al. 2015	74	61.5	109.5	37	32.0	92.0	Sedentary	EE	Overall	0.3 [-0.1; 0.7]	3.4%	0.164
Dunn AL et al. 1999	90	6.8	18.8	98	1.2	18.9	Sedentary	Time	Overall	0.3 [0.0; 0.6]	3.5%	0.042
Gill DP et al. 2019	59	0.1	0.2	59	0.0	0.2	Sedentary	Time	Overall	0.4 [0.0; 0.7]	3.4%	0.042
Lugones-Sanchez C et al. 2022	216	175.0	448.4	227	42.7	164.3	Sedentary	Time	Overall	0.4 [0.2; 0.6]	3.6%	< 0.001
Blake H et al. 2019	196	-5.7	11.5	86	-10.3	9.6	Sedentary	Time	Overall	0.4 [0.2; 0.7]	3.5%	0.001
Genin PM et al. 2017	73	5.4	12.7	22	0.1	8.9	Sedentary	Time	Overall	0.4 [-0.0; 0.9]	3.3%	0.071
Phaswana M et al. 2023	44	9.3	37.1	18	-7.7	36.4	Sedentary	Time	Overall	0.5 [-0.1; 1.0]	3.2%	0.109
Bergman F et al. 2018	37	36.0	70.5	38	-0.4	68.5	Sedentary	Time	Overall	0.5 [0.1; 1.0]	3.3%	0.027
Schuna JM Jr et al. 2014	15	1.1	3.1	16	-1.0	3.0	Sedentary	Time	Overall	0.7 [-0.1; 1.4]	2.9%	0.069
Edwardson CL et al. 2018	62	63.0	90.5	45	-8.0	83.7	Sedentary	Time	Overall	0.8 [0.4; 1.2]	3.4%	< 0.001
Pereira MA et al. 2020	263	38.8	43.9	224	-8.9	43.9	Sedentary	Time	Overall	1.1 [0.9; 1.3]	3.6%	< 0.001
Healy GN et al. 2016	119	77.9	40.6	83	0.2	78.3	Sedentary	Time	Overall	1.3 [1.0; 1.6]	3.5%	< 0.001
Puig-Ribera A et al. 2015	91	23.5	9.0	107	-1.8	9.0	Sedentary	Time	Overall	2.8 [2.4; 3.2]	3.4%	< 0.001
Hurling R et al. 2007	47	5.9	2.0	30	-1.4	2.7	Sedentary	Time	Overall	3.1 [2.5; 3.8]	3.0%	< 0.001
**Random effects model**	**2631**			**2194**						**0.4 [0.1; 0.7]**	**100.0%**	**0.013**
**Prediction interval**										**[-1.2; 1.9]**		

Heterogeneity: $I^2 = 93.3\%$, $\tau^2 = 0.5606$, $p < 0.0001$

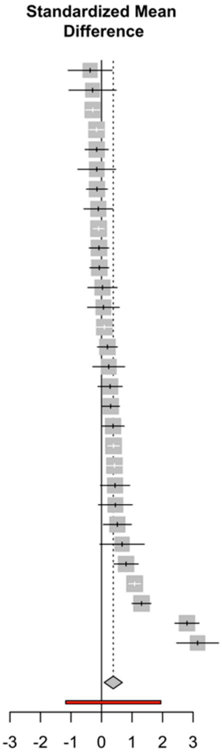

Supplementary Figure S5. Forest plot of subgroup meta-analysis for overall reducing sedentary behavior. The left side of the plot shows the study authors and publication year, the sample sizes of the intervention and control groups, the mean change, and the standard deviation of the mean change, with units and domain of the extracted data. The right side displays the standardized mean difference (SMD), the 95% confidence interval (95%CI) of the SMD, and the weight of each study. The lower side shows the overall analysis results for main meta-analysis from the random-effects model. The I² and τ² are indicators of heterogeneity, and p represents the p-value of the heterogeneity test. EE (energy expenditure), e.g., kcal/day, METs hour/day; Time, e.g., hours/day, min/day.

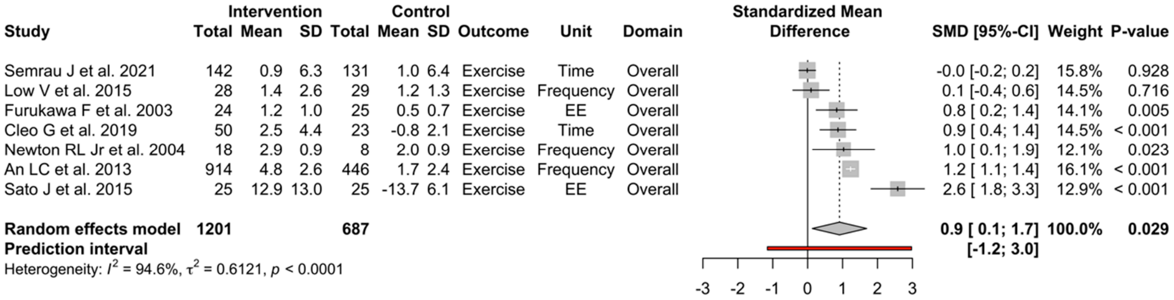

Supplementary Figure S6. Forest plot of subgroup meta-analysis for exercise behavior. The left side of the plot shows the study authors and publication year, the sample sizes of the intervention and control groups, the mean change, and the standard deviation of the mean change, with units and domain of the extracted data. The right side displays the standardized mean difference (SMD), the 95% confidence interval (95%CI) of the SMD, and the weight of each study. The lower side shows the overall analysis results for main meta-analysis from the random-effects model. The I^2 and τ^2 are indicators of heterogeneity, and p represents the p-value of the heterogeneity test. EE (energy expenditure), e.g., kcal/day, METs hour/day; Time, e.g., hours/day, min/day; Frequency, e.g., times/week, times/day.

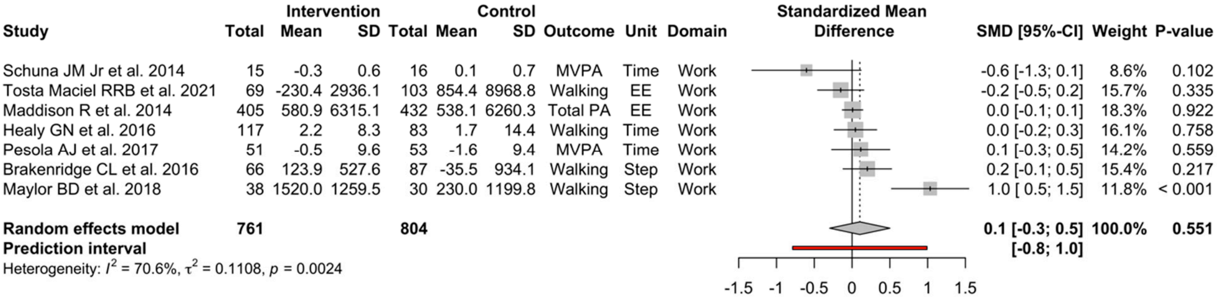

Supplementary Figure S7. Forest plot of subgroup meta-analysis for work-related physical activity (PA) metrics outcomes. The left side of the plot shows the study authors and publication year, the sample sizes of the intervention and control groups, the mean change, and the standard deviation of the mean change, with units and domain of the extracted data. The right side displays the standardized mean difference (SMD), the 95% confidence interval (95%CI) of the SMD, and the weight of each study. The lower side shows the overall analysis results for main meta-analysis from the random-effects model. The I^2 and τ^2 are indicators of heterogeneity, and p represents the p-value of the heterogeneity test. MVPA, moderate and vigorous physical activity. PA metrics outcomes were selected in the following order: MVPA > total PA > walking for each study. EE (energy expenditure), e.g., kcal/day, METs hour/day; Time, e.g., hours/day, min/day; Step, e.g., steps/day, steps/week.

Study	Intervention Total	Intervention Mean	Intervention SD	Control Total	Control Mean	Control SD	Outcome	Unit	Domain	Standardized Mean Difference	SMD [95%-CI]	Weight	P-value
Duggan C et al. 2014	159	-1.4	5.0	143	-1.0	6.0	Total PA	Frequency	Leisure		-0.1 [-0.3; 0.2]	8.4%	0.519
Tosta Maciel RRB et al. 2021	69	-193.7	1690.6	103	-128.1	1765.2	Walking	EE	Leisure		-0.0 [-0.3; 0.3]	8.4%	0.809
Semrau J et al. 2021	142	-0.4	5.5	131	-0.6	5.5	Total PA	Time	Leisure		0.0 [-0.2; 0.3]	8.4%	0.777
Pesola AJ et al. 2017	55	-0.9	14.8	53	-1.9	14.7	MVPA	Time	Leisure		0.1 [-0.3; 0.4]	8.3%	0.727
Annesi JJ 2012	247	15.8	18.4	183	13.3	17.1	Total PA	EE	Leisure		0.1 [-0.1; 0.3]	8.4%	0.152
Maddison R et al. 2014	405	526.0	1392.6	432	307.0	1373.9	Total PA	EE	Leisure		0.2 [0.0; 0.3]	8.5%	0.022
Annesi JJ et al. 2015	136	16.5	18.8	138	12.6	17.1	Total PA	EE	Leisure		0.2 [-0.0; 0.5]	8.4%	0.075
Rosas LG et al. 2020	92	35.8	226.0	99	-36.9	277.5	MVPA	Time	Leisure		0.3 [-0.0; 0.6]	8.4%	0.050
Merom D et al. 2007	207	82.7	87.0	107	55.0	96.3	Total PA	Time	Leisure		0.3 [0.1; 0.5]	8.4%	0.011
Eaglehouse YL et al. 2016	138	18.8	20.7	71	12.2	20.6	Total PA	EE	Leisure		0.3 [0.0; 0.6]	8.4%	0.032
Hurling R et al. 2007	47	4.1	2.6	30	-5.5	3.5	Total PA	EE	Leisure		3.2 [2.5; 3.9]	7.9%	< 0.001
Assuncao MC et al. 2010	97	88.4	35.2	95	-38.7	27.6	Total PA	Time	Leisure		4.0 [3.5; 4.5]	8.2%	< 0.001
**Random effects model**	**1794**			**1585**							**0.7 [-0.2; 1.6]**	**100.0%**	**0.100**
**Prediction interval**											**[-2.3; 3.7]**		

Heterogeneity: $I^2 = 96.5\%$, $\tau^2 = 1.7339$, $p < 0.0001$

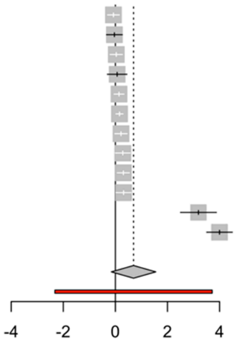

Supplementary Figure S8. Forest plot of subgroup meta-analysis for leisure-related physical activity (PA) metrics outcomes. The left side of the plot shows the study authors and publication year, the sample sizes of the intervention and control groups, the mean change, and the standard deviation of the mean change, with units and domain of the extracted data. The right side displays the standardized mean difference (SMD), the 95% confidence interval (95%CI) of the SMD, and the weight of each study. The lower side shows the overall analysis results for main meta-analysis from the random-effects model. The I^2 and τ^2 are indicators of heterogeneity, and p represents the p-value of the heterogeneity test. MVPA, moderate and vigorous physical activity. PA metrics outcomes were selected in the following order: MVPA > total PA > walking for each study. EE (energy expenditure), e.g., kcal/day, METs hour/day; Time, e.g., hours/day, min/day; Frequency, e.g., times/week, times/day.
