# Supplementary material for: Effectiveness and Components of Health Behavior Interventions on Increasing Physical Activity Among Healthy Young and Middle-Aged Adults: A Systematic Review with Meta-Analyses
Source: Behav Sci (Basel). 2024 Dec 19;14(12):1224. doi: 10.3390/bs14121224 (PMC11673272; doi:10.3390/bs14121224)
Supplement: Supplementary file 1 [file behavsci-14-01224-s001.zip › S5_Sup_MetaReg.pdf]

Supplementary Table S6. Result of meta-regressions and sensitivity analyses.

Outcome	BCT	Strategy 1										Strategy 2									
		Meta-regression					Sensitivity analysis of meta-regression					Meta-regression					Sensitivity analysis of meta-regression				
		k	β	SE	P	R2	k	β	SE	P	R2	k	β	SE	P	R2	k	β	SE	P	R2
Primary analysis																					
PA metric outcomes	1.1 Goal setting (behavior)	27	0.3	0.2	0.161	0.0%	53	0.3	0.2	0.246	0.0%	41	0.1	0.2	0.512	16.2%	39	0.1	0.2	0.684	18.3%
	1.2 Problem solving	6	-0.1	0.3	0.655		26	-0.2	0.3	0.600		23	-0.1	0.2	0.827		22	0.0	0.3	0.992	
	1.3 Goal setting (outcome)	15	-0.2	0.4	0.599		5	-0.3	0.4	0.469		4	0.1	0.4	0.732		3	0.1	0.5	0.819	
	1.4 Action planning	10	-0.3	0.3	0.314		13	-0.3	0.3	0.356		12	-0.2	0.3	0.426		10	-0.1	0.3	0.649	
	**1.5 Review behavior goal(s) ##**	26	1.0	0.4	**0.012*$&**		9	1.1	0.4	**0.012*$**		9	0.7	0.3	**0.048*&**		8	0.7	0.4	0.055	
	2.2 Feedback on behavior	52	-0.1	0.3	0.567		25	-0.1	0.3	0.661		20	0.0	0.2	0.854		19	0.0	0.2	0.850	
	2.3 Self-monitoring of behavior	3	0.2	0.2	0.339		49	0.2	0.2	0.313		39	0.3	0.2	0.119		36	0.3	0.2	0.170	
	2.4 Self-monitoring of outcome(s) of behavior	6	0.5	0.7	0.428		3	0.5	0.7	0.460		1	-	-	-		1	-	-	-	
	2.6 Biofeedback	3	-0.3	0.4	0.466		6	-0.3	0.4	0.473		1	-	-	-		1	-	-	-	
	2.7 Feedback on outcome(s) of behavior	52	-0.7	0.7	0.366		3	-0.7	0.8	0.398		0	-	-	-		0	-	-	-	
	3.1 Social support (unspecified)	14	-0.3	0.2	0.179		49	-0.3	0.2	0.230		25	-0.1	0.2	0.478		22	-0.1	0.2	0.700	
	**3.2 Social support (practical) #**	7	-0.5	0.3	0.146		13	-0.5	0.4	0.208		11	-0.9	0.4	**0.028*$**		10	-0.8	0.4	**0.042*$**	
	**3.3 Social support (emotional) #**	45	0.7	0.4	0.118		7	0.7	0.5	0.166		6	1.0	0.5	**0.041***		6	1.0	0.5	0.054	
	4.1 Instruction on how to perform a behavior	38	0.2	0.2	0.421		42	0.2	0.2	0.400		21	0.2	0.2	0.282		19	0.3	0.2	0.218	
	5.1 Information about health consequences	3	0.0	0.2	0.828		34	0.0	0.2	0.879		14	0.1	0.2	0.500		12	0.1	0.2	0.455	
	5.2 Salience of consequences	26	0.0	0.6	0.969		2	-	-	-		3	0.0	0.5	0.921		2	-	-	-	

Outcome	BCT	Strategy 1										Strategy 2									
		Meta-regression					Sensitivity analysis of meta-regression					Meta-regression					Sensitivity analysis of meta-regression				
		k	β	SE	P	R2	k	β	SE	P	R2	k	β	SE	P	R2	k	β	SE	P	R2
	6.1 Demonstration of the behavior	24	0.0	0.2	0.893		24	0.0	0.2	0.894		17	0.0	0.2	0.816		15	0.0	0.2	0.992	
	7.1 Prompts/cues	9	0.1	0.2	0.699		24	0.1	0.2	0.811		21	0.1	0.2	0.711		21	0.1	0.2	0.828	
	8.1 Behavioral practice/ rehearsal	16	-0.3	0.3	0.413		9	-0.4	0.4	0.305		7	-0.1	0.3	0.669		7	-0.2	0.3	0.460	
	8.3 Habit formation	3	0.5	0.3	0.127		14	0.6	0.3	0.089		13	0.5	0.3	0.098		11	0.6	0.3	**0.041***	
	8.7 Graded tasks	8	-0.6	0.6	0.332		3	-0.6	0.6	0.322		3	-0.4	0.5	0.463		3	-0.4	0.5	0.482	
	10.1 Material incentive (behavior)	6	0.0	0.4	0.939		7	-0.1	0.4	0.733		2	-	-	-		2	-	-	-	
	10.2 Material reward (behavior)	5	0.3	0.4	0.501		5	0.4	0.5	0.412		5	0.5	0.5	0.269		4	0.6	0.5	0.279	
	10.4 Social reward	10	-0.4	0.4	0.378		5	-0.4	0.4	0.338		5	-0.2	0.4	0.507		5	-0.3	0.4	0.491	
	12.1 Restructuring the physical environment	4	-0.2	0.4	0.493		10	-0.3	0.4	0.437		7	-0.4	0.3	0.158		7	-0.4	0.3	0.150	
	12.2 Restructuring the social environment	3	-0.3	0.5	0.497		4	-0.3	0.5	0.480		3	-0.7	0.5	0.134		3	-0.9	0.5	0.086	
	12.5 Adding objects to the environment	18	0.3	0.6	0.560		3	0.3	0.6	0.591		1	-	-	-		1	-	-	-	
Subgroup analyses																					
MVPA	1.1 Goal setting (behavior)	8	0.1	0.2	0.724	5.7%	17	0.2	0.2	0.375	60.2%	16	0.2	0.4	0.515	0.0%	15	0.3	0.3	0.336	14.7%
	**1.2 Problem solving #**	3	-0.7	0.3	**0.039*$**		8	-0.9	0.2	**0.005*$**		7	-0.4	0.3	0.216		7	-0.7	0.3	0.056	
	1.4 Action planning	4	-0.2	0.6	0.769		3	-0.2	0.4	0.592		2	-	-	-		2	-	-	-	
	1.5 Review behavior goal(s)	7	0.3	0.4	0.537		4	0.5	0.3	0.184		3	0.1	0.4	0.815		3	0.4	0.4	0.363	
	2.2 Feedback on behavior	18	0.3	0.3	0.437		7	0.2	0.3	0.508		6	0.0	0.3	0.924		6	-0.3	0.4	0.408	
	2.3 Self-monitoring of behavior	18	0.1	0.3	0.626		17	0.2	0.2	0.429		12	0.2	0.4	0.701		11	0.3	0.4	0.399	
	3.1 Social support (unspecified)	7	0.2	0.3	0.567		18	0.0	0.2	0.943		9	0.0	0.3	0.877		9	-0.1	0.2	0.718	

Outcome	BCT	Strategy 1										Strategy 2									
		Meta-regression					Sensitivity analysis of meta-regression					Meta-regression					Sensitivity analysis of meta-regression				
		k	β	SE	P	R2	k	β	SE	P	R2	k	β	SE	P	R2	k	β	SE	P	R2
	3.2 Social support (practical)	3	0.4	0.3	0.248		6	0.6	0.3	**0.040***		6	-0.1	0.3	0.744		5	0.5	0.5	0.272	
	3.3 Social support (emotional)	16	0.2	0.5	0.707		3	-0.1	0.4	0.822		3	0.1	0.7	0.887		3	-0.5	0.7	0.516	
	4.1 Instruction on how to perform a behavior	14	-0.1	0.2	0.669		16	-0.1	0.2	0.678		7	0.0	0.4	0.934		7	0.1	0.3	0.711	
	5.1 Information about health consequences	5	-0.4	0.2	0.103		13	-0.2	0.2	0.171		5	-0.1	0.3	0.661		5	0.0	0.3	0.911	
	6.1 Demonstration of the behavior	8	-0.2	0.3	0.555		5	-0.2	0.2	0.452		2	-	-	-		2	-	-	-	
	**7.1 Prompts/cues #**	3	-0.4	0.3	0.152		8	-0.5	0.2	**0.038*&**		8	-0.4	0.2	0.094		8	-0.5	0.2	**0.040*&**	
	10.1 Material incentive (behavior)	3	-0.5	0.3	0.127		3	-0.5	0.2	0.057		1	-	-	-		1	-	-	-	
	10.4 Social reward	6	0.3	0.4	0.524		3	0.3	0.3	0.396		3	-0.1	0.4	0.691		3	-0.3	0.3	0.383	
	12.1 Restructuring the physical environment	3	-0.3	0.3	0.421		6	-0.4	0.3	0.122		5	-0.3	0.3	0.463		5	-0.6	0.3	0.133	
	12.2 Restructuring the social environment	26	0.0	0.4	0.912		3	0.1	0.3	0.756		2	-	-	-		2	-	-	-	
Total PA	1.1 Goal setting (behavior)	16	0.2	0.2	0.317	15.0%	25	0.1	0.2	0.757	32.1%	18	0.2	0.2	0.426	12.3%	17	0.0	0.2	0.809	30.0%
	1.2 Problem solving	3	-0.3	0.3	0.267		15	-0.2	0.3	0.380		15	-0.1	0.2	0.682		14	0.1	0.3	0.710	
	1.3 Goal setting (outcome)	11	-0.1	0.4	0.812		2	-	-	-		2	-	-	-		1	-	-	-	
	1.4 Action planning	3	0.0	0.3	0.898		9	0.1	0.2	0.812		9	-0.1	0.3	0.741		7	0.1	0.2	0.599	
	1.5 Review behavior goal(s)	10	0.1	0.5	0.868		2	-	-	-		3	0.2	0.5	0.743		2	-	-	-	
	**2.2 Feedback on behavior #**	25	0.8	0.3	**0.014*$**		9	1.0	0.3	**0.002*$**		8	0.6	0.3	0.092		7	0.6	0.3	0.078	
	2.3 Self-monitoring of behavior	29	-0.1	0.3	0.796		23	-0.1	0.3	0.761		21	0.1	0.2	0.610		19	0.0	0.2	0.965	
	3.1 Social support (unspecified)	4	-0.2	0.2	0.334		26	-0.1	0.2	0.584		13	-0.1	0.2	0.713		10	0.1	0.2	0.686	
	3.2 Social support (practical)	24	-0.5	0.4	0.168		4	-0.5	0.3	0.124		4	-0.5	0.4	0.161		4	-0.5	0.3	0.130	

Outcome	BCT	Strategy 1										Strategy 2									
		Meta-regression					Sensitivity analysis of meta-regression					Meta-regression					Sensitivity analysis of meta-regression				
		k	β	SE	P	R2	k	β	SE	P	R2	k	β	SE	P	R2	k	β	SE	P	R2
	4.1 Instruction on how to perform a behavior	14	0.1	0.2	0.766		21	0.0	0.2	0.972		11	0.2	0.2	0.378		9	0.2	0.2	0.239	
	5.1 Information about health consequences	17	0.3	0.2	0.194		11	0.4	0.2	0.110		4	0.0	0.2	0.991		2	-	-	-	
	6.1 Demonstration of the behavior	12	0.1	0.3	0.689		15	0.1	0.3	0.808		14	0.1	0.2	0.462		12	0.2	0.2	0.421	
	7.1 Prompts/cues	8	0.2	0.3	0.512		12	0.1	0.2	0.737		10	-0.1	0.3	0.847		10	-0.1	0.3	0.701	
	8.1 Behavioral practice/ rehearsal	15	0.0	0.3	0.855		8	-0.1	0.3	0.710		6	-0.1	0.3	0.742		6	-0.2	0.2	0.466	
	**8.3 Habit formation #**	4	0.4	0.3	0.121		13	0.6	0.3	**0.021*&**		12	0.4	0.2	0.084		10	0.7	0.2	**0.012*&**	
	10.1 Material incentive (behavior)	3	-0.5	0.4	0.229		3	-0.9	0.4	0.054		1	-	-	-		1	-	-	-	
	12.2 Restructuring the social environment	27	-0.3	0.4	0.497		3	-0.4	0.4	0.284		3	-0.5	0.4	0.189		3	-0.8	0.4	0.052	
Walking	1.1 Goal setting (behavior)	11	0.2	0.4	0.553	8.1%	15	0.2	0.5	0.614	11.0%	20	0.2	0.3	0.513	51.9%	10	0.2	0.4	0.641	39.6%
	1.2 Problem solving	3	0.3	0.4	0.549		8	0.4	0.5	0.415		8	-0.1	0.4	0.861		6	0.3	0.5	0.574	
	1.3 Goal setting (outcome)	7	-0.1	0.7	0.944		2	-0.2	0.8	0.839		2	-	-	-		1	-	-	-	
	1.4 Action planning	7	-0.3	0.5	0.569		5	-0.3	0.5	0.604		6	0.1	0.5	0.903		4	-0.1	0.6	0.884	
	1.5 Review behavior goal(s)	15	0.8	0.5	0.108		3	0.5	0.6	0.397		6	0.2	0.4	0.614		2	0.2	0.5	0.735	
	2.2 Feedback on behavior	28	-0.8	0.4	0.066		12	-0.8	0.5	0.089		11	-0.2	0.3	0.450		9	-0.5	0.4	0.202	
	**2.3 Self-monitoring of behavior #**	20	0.5	0.4	0.235		16	0.5	0.5	0.284		22	0.6	0.3	**0.021*$**		11	0.8	0.4	**0.033*$**	
	3.1 Social support (unspecified)	7	-0.7	0.4	0.093		13	-0.9	0.5	0.070		12	-0.1	0.3	0.706		6	-0.4	0.4	0.277	
	3.2 Social support (practical)	4	-1.0	0.6	0.121		4	-1.1	0.7	0.123		4	-2.9	0.7	**<0.001***		2	-1.5	1.1	0.190	
	**3.3 Social support (emotional) #**	19	1.4	0.7	0.064		3	1.5	0.8	0.073		3	3.4	0.8	**<0.001*$**		2	2.6	0.8	**0.005*$**	

Outcome	BCT	Strategy 1										Strategy 2									
		Meta-regression					Sensitivity analysis of meta-regression					Meta-regression					Sensitivity analysis of meta-regression				
		k	β	SE	P	R2	k	β	SE	P	R2	k	β	SE	P	R2	k	β	SE	P	R2
	4.1 Instruction on how to perform a behavior	16	-0.3	0.4	0.460		14	-0.4	0.4	0.333		10	-0.2	0.3	0.507		7	-0.3	0.3	0.392	
	5.1 Information about health consequences	7	0.1	0.3	0.799		9	0.3	0.4	0.513		7	0.2	0.2	0.336		4	0.4	0.3	0.188	
	6.1 Demonstration of the behavior	12	-0.3	0.5	0.567		4	-0.5	0.6	0.449		4	-0.2	0.5	0.611		1	-1.1	0.9	0.223	
	7.1 Prompts/cues	4	0.1	0.4	0.885		9	0.1	0.4	0.868		11	0.4	0.3	0.257		8	0.3	0.4	0.424	
	8.3 Habit formation	4	0.1	0.6	0.859		3	-0.2	0.7	0.745		4	-0.2	0.6	0.719		3	-0.7	0.7	0.325	
	10.2 Material reward (behavior)	4	-0.2	0.7	0.781		2	-0.3	0.9	0.700		3	2.2	0.7	**0.003***		1	-	-	-	
	10.4 Social reward	4	-0.3	0.6	0.639		2	-0.2	0.6	0.813		4	-0.3	0.4	0.456		2	-0.2	0.5	0.735	
	12.1 Restructuring the physical environment	19	-0.3	0.6	0.597		1	-0.3	0.7	0.666		3	0.2	0.4	0.672		0	0.4	0.5	0.418	
Reducing sedentary behavior	1.1 Goal setting (behavior)	12	-0.4	0.5	0.448	0.0%	19	-0.3	1.0	0.796	0.0%	15	-0.6	0.5	0.198	24.8%	15	-0.3	0.6	0.600	0.0%
	1.2 Problem solving	9	0.6	0.9	0.543		11	0.0	1.3	0.992		11	-0.8	0.7	0.273		10	0.1	0.8	0.948	
	1.4 Action planning	6	0.7	1.1	0.536		8	0.2	1.3	0.884		8	0.2	0.6	0.792		7	0.3	0.5	0.612	
	1.5 Review behavior goal(s)	5	1.8	0.9	0.075		6	-	-	-		6	-0.1	0.7	0.906		6	-	-	-	
	2.2 Feedback on behavior	11	-0.5	0.9	0.573		5	-0.1	1.0	0.902		5	1.2	0.7	0.126		5	-0.2	0.7	0.714	
	2.3 Self-monitoring of behavior	3	-0.5	0.7	0.549		11	-0.3	1.3	0.867		9	0.4	0.4	0.346		9	0.1	0.4	0.858	
	2.6 Biofeedback	17	0.1	1.0	0.939		3	-	-	-		0	-	-	-		0	-	-	-	
	3.1 Social support (unspecified)	7	0.0	0.6	0.951		16	-0.1	1.2	0.960		7	0.9	0.4	**0.030***		6	-	-	-	
	3.2 Social support (practical)	4	-0.1	0.9	0.896		7	0.1	0.8	0.951		6	-1.2	0.6	0.053		6	0.1	0.5	0.795	
	3.3 Social support (emotional)	16	0.3	0.7	0.706		4	-	-	-		4	2.2	0.8	**0.017***		4	-	-	-	

Outcome	BCT	Strategy 1										Strategy 2									
		Meta-regression					Sensitivity analysis of meta-regression					Meta-regression					Sensitivity analysis of meta-regression				
		k	β	SE	P	R2	k	β	SE	P	R2	k	β	SE	P	R2	k	β	SE	P	R2
	4.1 Instruction on how to perform a behavior	14	0.5	0.6	0.434		15	0.3	1.0	0.796		7	-0.4	0.3	0.310		6	-	-	-	
	5.1 Information about health consequences	8	-0.9	0.6	0.209		13	-0.2	1.0	0.845		8	0.7	0.4	0.127		7	0.1	0.5	0.773	
	6.1 Demonstration of the behavior	10	-0.9	0.9	0.311		7	-0.7	1.2	0.616		5	1.0	0.7	0.169		4	-	-	-	
	7.1 Prompts/cues	4	-0.2	0.8	0.798		10	-0.1	1.1	0.913		8	1.0	0.6	0.112		8	0.3	0.4	0.568	
	8.1 Behavioral practice/ rehearsal	6	-1.8	0.9	0.062		4	-0.3	1.1	0.800		3	-0.3	0.9	0.712		3	-0.1	0.7	0.883	
	8.3 Habit formation	9	0.1	0.9	0.935		5	0.0	0.9	0.993		5	-0.9	0.7	0.245		4	-0.1	0.5	0.822	
	12.1 Restructuring the physical environment	3	-0.1	0.5	0.851		9	0.0	0.6	0.945		5	0.0	0.3	0.894		5	-	-	-	
	12.2 Restructuring the social environment	5	-0.1	1.0	0.902		3	0.4	1.3	0.773		2	-	-	-		2	-	-	-	
Leisure-related PA metric outcomes	1.1 Goal setting (behavior)	5	-2.1	1.6	0.324	0.0%	4	-5.6	3.6	0.365	0.0%	4	-1.7	1.5	0.313	0.0%	3	-3.4	2.8	0.307	0.0%
	1.2 Problem solving	4	0.3	2.0	0.910		5	4.0	4.0	0.498		4	0.8	2.2	0.721		4	2.6	3.4	0.502	
	2.2 Feedback on behavior	3	-4.3	3.2	0.310		4	-2.0	3.7	0.691		3	0.3	1.5	0.873		3	0.3	1.8	0.862	
	2.3 Self-monitoring of behavior	4	5.0	2.7	0.203		3	6.8	3.1	0.270		3	1.5	1.9	0.473		3	3.0	3.0	0.393	
	3.1 Social support (unspecified)	5	1.5	1.2	0.338		4	2.4	1.4	0.342		1	-	-	-		1	-	-	-	
	4.1 Instruction on how to perform a behavior	3	-2.3	1.6	0.289		5	-1.1	1.9	0.657		2	-	-	-		3	-0.5	1.8	0.820	
	6.1 Demonstration of the behavior	3	1.2	1.7	0.545		3	-0.2	2.1	0.925		1	-	-	-		1	-	-	-	

Outcome	BCT	Strategy 1										Strategy 2									
		Meta-regression					Sensitivity analysis of meta-regression					Meta-regression					Sensitivity analysis of meta-regression				
		k	β	SE	P	R2	k	β	SE	P	R2	k	β	SE	P	R2	k	β	SE	P	R2
	7.1 Prompts/cues	3	1.0	3.3	0.785		3	-5.4	6.7	0.572		3	-1.2	3.0	0.701		3	-3.2	4.4	0.527	

Note: *****, $p < 0.05$; **$**, significance was confirmed in both meta-analysis and sensitivity analysis in same meta-regression strategy; **&**, significance was confirmed in both meta-regression and sensitivity analysis in same analysis; #, was considered as potentially related behavior change technique; ##, was considered as related behavior change technique; **PA**, physical activity; **MVPA**, moderate and vigorous PA; **Strategy 1**, an aggressive method that ignored the BCTs in the control groups and used only those in the intervention groups; **Strategy 2**, a conservative method that subtracted the control group BCTs from the intervention group BCTs before conducting the meta-regression. PA metric outcomes were selected in the following order: MVPA > total PA > walking for each study. Studies with low risk and high risk were excluded from the analyses for reducing sedentary behavior, and studies with high risk were excluded from the analyses for other PA outcomes. The restricted maximum likelihood method was used in meta-regressions and sensitivity analyses of meta-regressions.
