# Supplementary material for: Effectiveness and Components of Health Behavior Interventions on Increasing Physical Activity Among Healthy Young and Middle-Aged Adults: A Systematic Review with Meta-Analyses
Source: Behav Sci (Basel). 2024 Dec 19;14(12):1224. doi: 10.3390/bs14121224 (PMC11673272; doi:10.3390/bs14121224)
Supplement: Supplementary file 1 [file behavsci-14-01224-s001.zip › S6_Sup_Bias.pdf]

Supplementary Table S7. Heterogeneity in meta-analyses, meta-regressions with subsequent sensitivity

Outcome	Meta-analyses								Meta-regressions							
	Meta-analysis				Sensitivity analysis of meta-analysis				Meta-regression				Sensitivity analysis of meta-regression			
	k	I^2	τ^2	P	k	I^2	τ^2	P	k	I^2	τ^2	P	k	I^2	τ^2	P
Primary analysis																
PA metric outcomes	102	92.3%	0.550	<0.001*	95	45.5%	0.022	<0.001*	90	89.6%	0.500	<0.001*	84	90.1%	0.534	<0.001*
Subgroup analyses																
MVPA	36	75.0%	0.113	<0.001*	35	75.7%	0.120	<0.001*	29	77.2%	0.128	<0.001*	28	78.0%	0.137	<0.001*
Total PA[a]	48	93.6%	0.449	<0.001*	42	87.9%	0.323	<0.001*	44	86.5%	0.264	<0.001*	39	87.6%	0.290	<0.001*
Walking[b]	47	91.4%	0.718	<0.001*	43	95.0%	1.506	<0.001*	43	92.0%	0.782	<0.001*	39	92.7%	0.871	<0.001*
Reducing sedentary behavior	30	93.3%	0.561	<0.001*	29	86.9%	0.118	<0.001*	30	93.3%	0.561	<0.001*	29	86.9%	0.118	<0.001*
Exercise behavior	7	94.6%	0.612	<0.001*	3	97.6%	0.409	<0.001*	4	-	-	-	1	-	-	-
Work-related PA metric outcomes	7	70.6%	0.111	0.002*	7	70.6%	0.111	0.002*	7	-	-	-	7	-	-	-
Leisure-related PA metric outcomes[a]	12	96.5%	1.734	<0.001*	11	23.5%	0.004	0.235	11	96.8%	1.904	<0.001*	10	97.1%	2.087	<0.001*

Note: *, $p < 0.05$; **k**, numbers of studies; **I^2** and **τ^2**, indicators of heterogeneity; **a**, removed outliers in sensitivity analysis; **b**, trimmed and filled in sensitivity analysis; **PA**, physical activity; **MVPA**, moderate and vigorous PA. PA metric outcomes were selected in the following order: MVPA > total PA > walking for each study. Studies with low risk and high risk were excluded in analyses for reducing sedentary behavior, and studies with high risk were excluded in analyses for other PA outcomes.

Supplementary Table S8. Impact of study quality to analyses

Outcome	Risk	Meta-analysis				Meta-regression			
		k	β	SE	P	k	β	SE	P
PA metric outcomes	(intercept)		0.2	0.1	**0.016***		0.2	0.1	**0.030***
	Low risk	31	0.2	0.2	0.222	26	0.3	0.2	0.105
	High risk	7	0.5	0.3	0.121	6	0.2	0.3	0.607
MVPA	(intercept)		0.1	0.1	0.112		0.1	0.1	0.195
	Low risk	11	-0.1	0.1	0.329	8	-0.1	0.2	0.428
	High risk	1	-0.1	0.4	0.785	1	-0.1	0.4	0.817
Total PA[a]	(intercept)		0.3	0.1	**0.032***		0.3	0.1	**0.026***
	Low risk	12	0.1	0.2	0.689	11	0.2	0.2	0.380
	High risk	6	0.6	0.3	0.088	5	0.2	0.3	0.493
Walking[b]	(intercept)		0.4	0.2	**0.041***		0.4	0.2	**0.044***
	Low risk	17	0.2	0.3	0.422	14	0.3	0.3	0.404
	High risk	4	0.2	0.5	0.713	4	0.2	0.5	0.749
Reducing sedentary behavior	(intercept)		0.1	0.2	0.419		0.1	0.2	0.419
	Low risk	11	0.6	0.3	**0.044***	11	0.6	0.3	**0.044***
	High risk	1	0.3	0.8	0.719	1	0.3	0.8	0.719
Exercise behavior	(intercept)		0.7	0.5	0.223		0.0	1.2	0.994
	High risk	4	0.4	0.7	0.572	3	1.2	1.4	0.504
Work-related PA metric outcomes	(intercept)		0.0	0.2	0.853		0.0	0.2	0.853
	Low risk	2	0.5	0.3	0.194	2	0.5	0.3	0.194
Leisure-related PA metric outcomes[a]	(intercept)		0.1	0.5	0.797		0.1	0.5	0.796
	Low risk	5	1.4	0.8	0.113	4	1.7	0.8	0.077
	High risk	1	0.0	1.4	0.998	1	0.0	1.4	0.998

Note: *, $p < 0.05$; **k**, numbers of studies; **β**, coefficient**;** **SE**, standard error; **a**, removed outliers in sensitivity analysis; **b**, trimmed and filled in sensitivity analysis; **PA**, physical activity; **MVPA**, moderate and vigorous PA. PA metric outcomes were selected in the following order: MVPA > total PA > walking for each study. Studies with low risk and high risk were excluded in analyses for reducing sedentary behavior, and studies with high risk were excluded in analyses for other PA outcomes.

Supplementary Table S9. Publication bias in each analysis

Outcome	Meta-analyses				Meta-regressions			
	Meta-analysis		Sensitivity analysis of meta-analysis		Meta-regression		Sensitivity analysis of meta-regression	
	k	P	k	P	k	P	k	P
Primary analysis								
PA metric outcomes	102	**0.017***	95	0.068	90	**0.012***	84	0.259
Subgroup analyses								
MVPA	36	0.410	35	0.592	0	0.592	28	0.610
Total PA[a]	48	0.284	42	0.065	44	0.132	39	0.275
Walking[b]	47	**0.012***	43	0.898	43	**0.012***	39	**0.011***
Reducing sedentary behavior	30	0.592	29	0.330	30	0.592	29	0.587
Exercise behavior	7	(0.684)	3	(0.179)	4	-	1	-
Work-related PA metric outcomes	7	(0.602)	7	(0.602)	7	-	7	-
Leisure-related PA metric outcomes[a]	12	**0.026***	11	0.920	11	**0.035***	10	**0.048***

Note: *****, p < 0.05; **a**, removed outliers in sensitivity analysis; **b**, trimmed and filled in sensitivity analysis; **PA**, physical activity; **MVPA**, moderate and vigorous PA. PA metric outcomes were selected in the following order: MVPA > total PA > walking for each study. Studies with low risk and high risk were excluded in analyses for reducing sedentary behavior, and studies with high risk were excluded in analyses for other PA outcomes. Egger's test was used for test of publication bias.

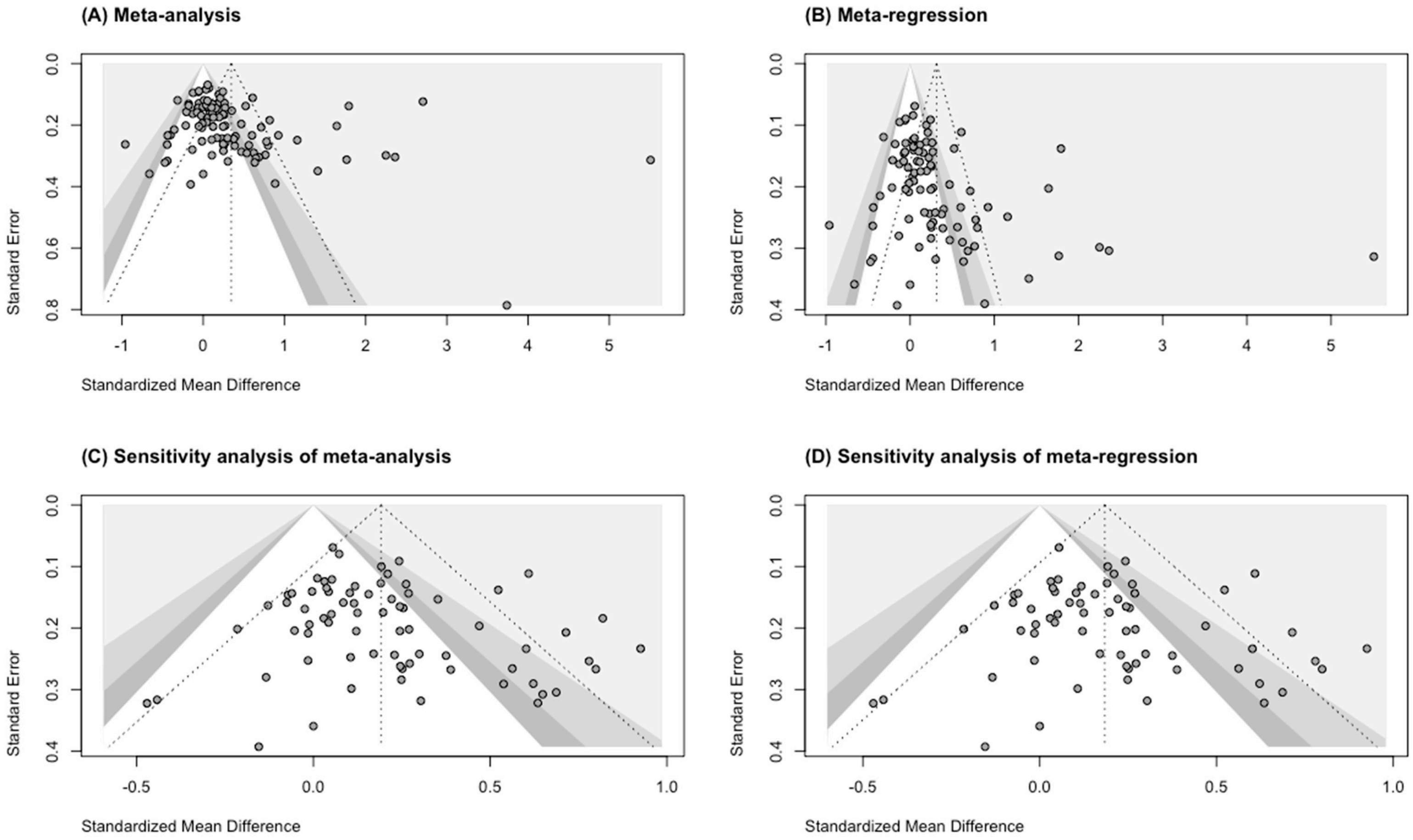

Supplementary Figure S9. Funnel plot of primary meta-analysis for physical activity (PA) metrics outcomes. PA metrics outcomes were selected in the following order: moderate and vigorous PA > total PA > walking for each study. Each point corresponds to an included study. The x-axis indicates the standardized mean difference, whereas the y-axis indicates the standard error of standardized mean difference (SMD) for each study. The white, dark gray, medium gray, and light gray areas represent confidence intervals within 99%, 95%–99%, 90%–95%, and beyond 90% when SMD = 0, respectively. The dashed triangular vertical line and edges represent the SMD mean and 95% confidence interval in meta-analysis and meta-regression. When the points are symmetrically distributed on both sides of the vertical line in the triangle, publication bias is minimal.

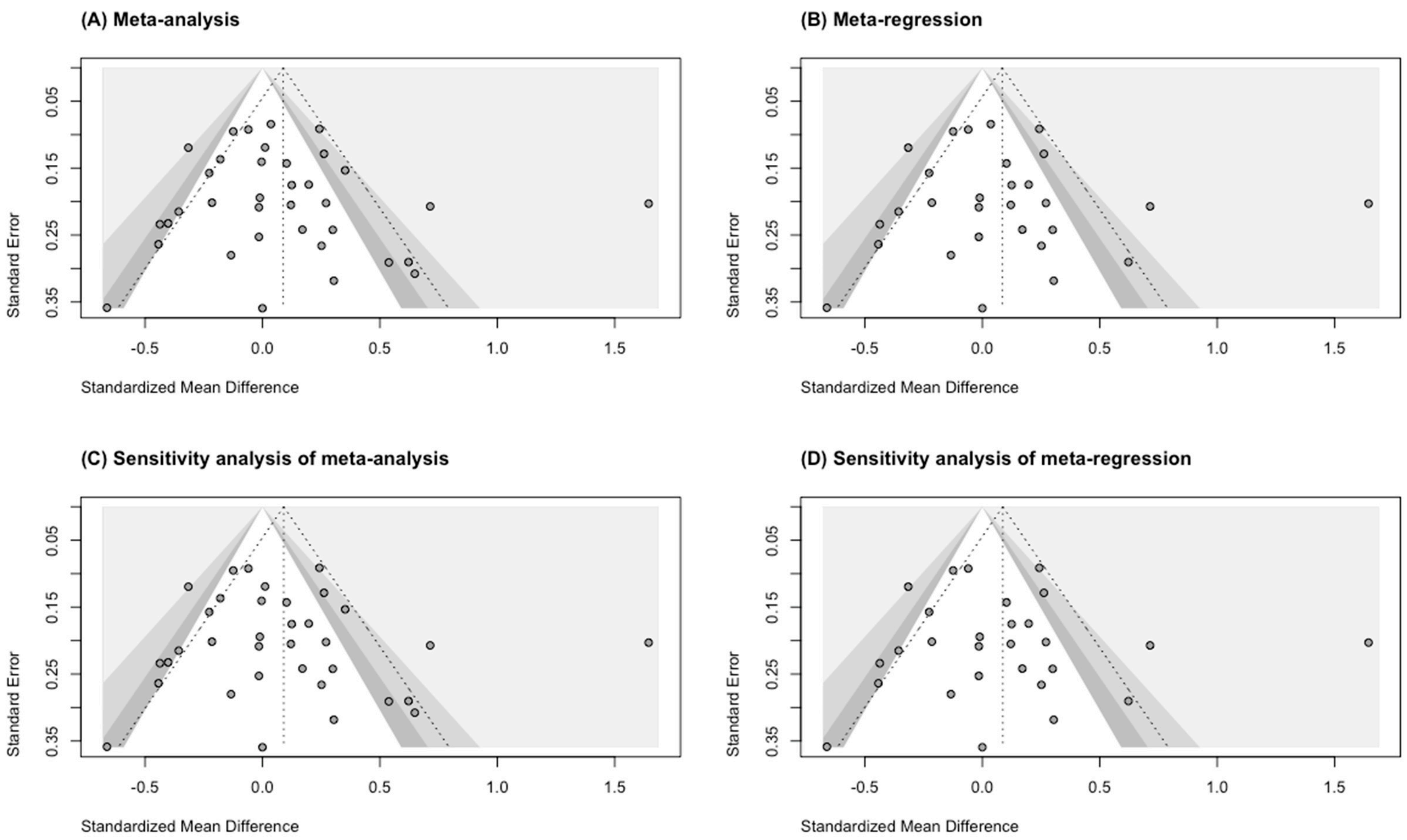

Supplementary Figure S10. Funnel plot of primary meta-analysis for moderate and vigorous physical activity metrics outcomes. Each point corresponds to an included study. The x-axis indicates the standardized mean difference, whereas the y-axis indicates the standard error of standardized mean difference (SMD) for each study. The white, dark gray, medium gray, and light gray areas represent confidence intervals within 99%, 95%–99%, 90%–95%, and beyond 90% when SMD = 0, respectively. The dashed triangular vertical line and edges represent the SMD mean and 95% confidence interval in meta-analysis and meta-regression. When the points are symmetrically distributed on both sides of the vertical line in the triangle, publication bias is minimal.

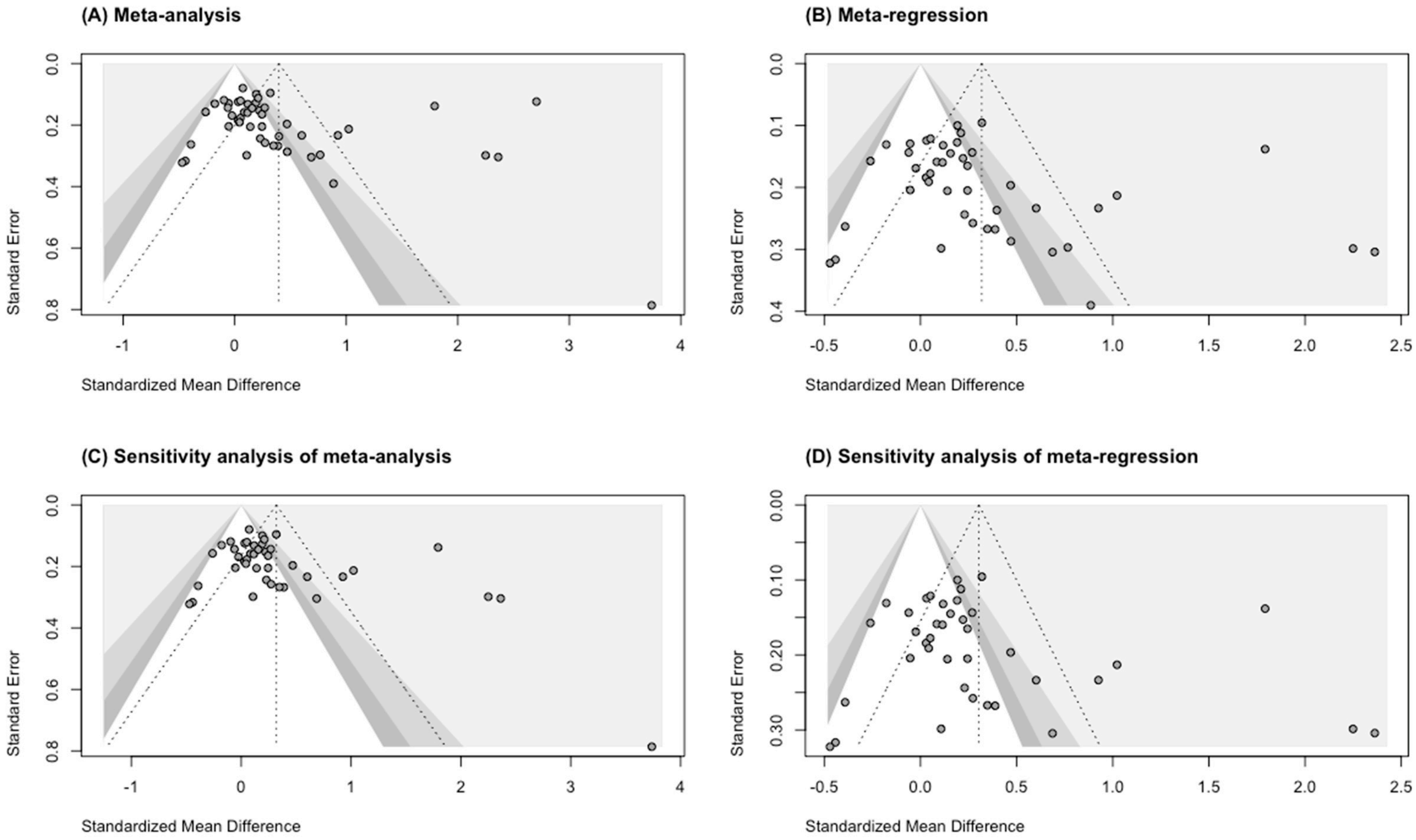

Supplementary Figure S11. Funnel plot of primary meta-analysis for total physical activity metrics outcomes. Each point corresponds to an included study. The x-axis indicates the standardized mean difference, whereas the y-axis indicates the standard error of standardized mean difference (SMD) for each study. The white, dark gray, medium gray, and light gray areas represent confidence intervals within 99%, 95%–99%, 90%–95%, and beyond 90% when SMD = 0, respectively. The dashed triangular vertical line and edges represent the SMD mean and 95% confidence interval in meta-analysis and meta-regression. When the points are symmetrically distributed on both sides of the vertical line in the triangle, publication bias is minimal.

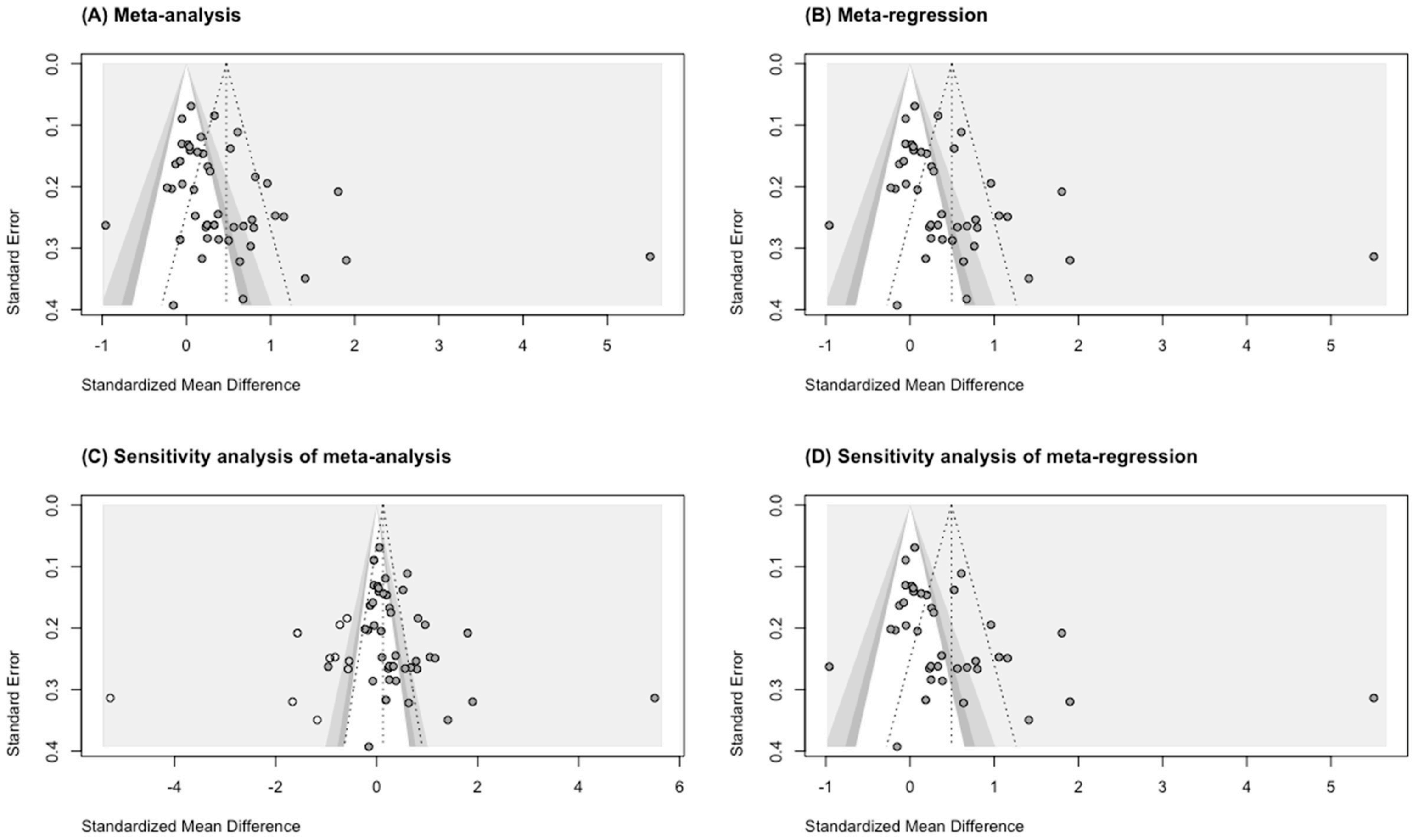

Supplementary Figure S12. Funnel plot of primary meta-analysis for walking. Each gray point corresponds to an included study, and each white point corresponds to a filled study. The x-axis indicates the standardized mean difference, whereas the y-axis indicates the standard error of standardized mean difference (SMD) for each study. The white, dark gray, medium gray, and light gray areas represent confidence intervals within 99%, 95%–99%, 90%–95%, and beyond 90% when SMD = 0, respectively. The dashed triangular vertical line and edges represent the SMD mean and 95% confidence interval in meta-analysis and meta-regression. When the points are symmetrically distributed on both sides of the vertical line in the triangle, publication bias is minimal.

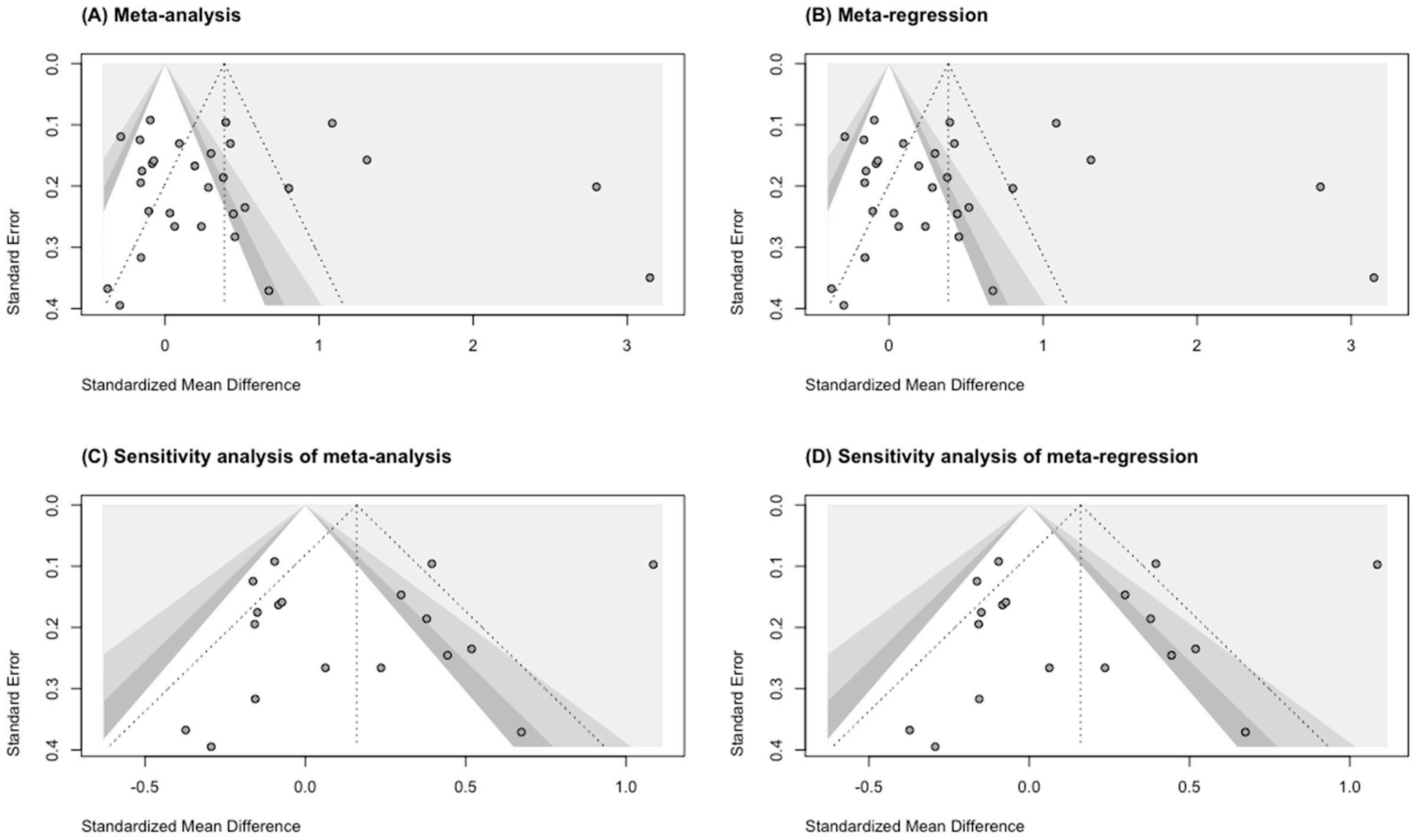

Supplementary Figure S13. Funnel plot of primary meta-analysis for reducing sedentary behavior. Each point corresponds to an included study. The x-axis indicates the standardized mean difference, whereas the y-axis indicates the standard error of standardized mean difference (SMD) for each study. The white, dark gray, medium gray, and light gray areas represent confidence intervals within 99%, 95%–99%, 90%–95%, and beyond 90% when SMD = 0, respectively. The dashed triangular vertical line and edges represent the SMD mean and 95% confidence interval in meta-analysis and meta-regression. When the points are symmetrically distributed on both sides of the vertical line in the triangle, publication bias is minimal.

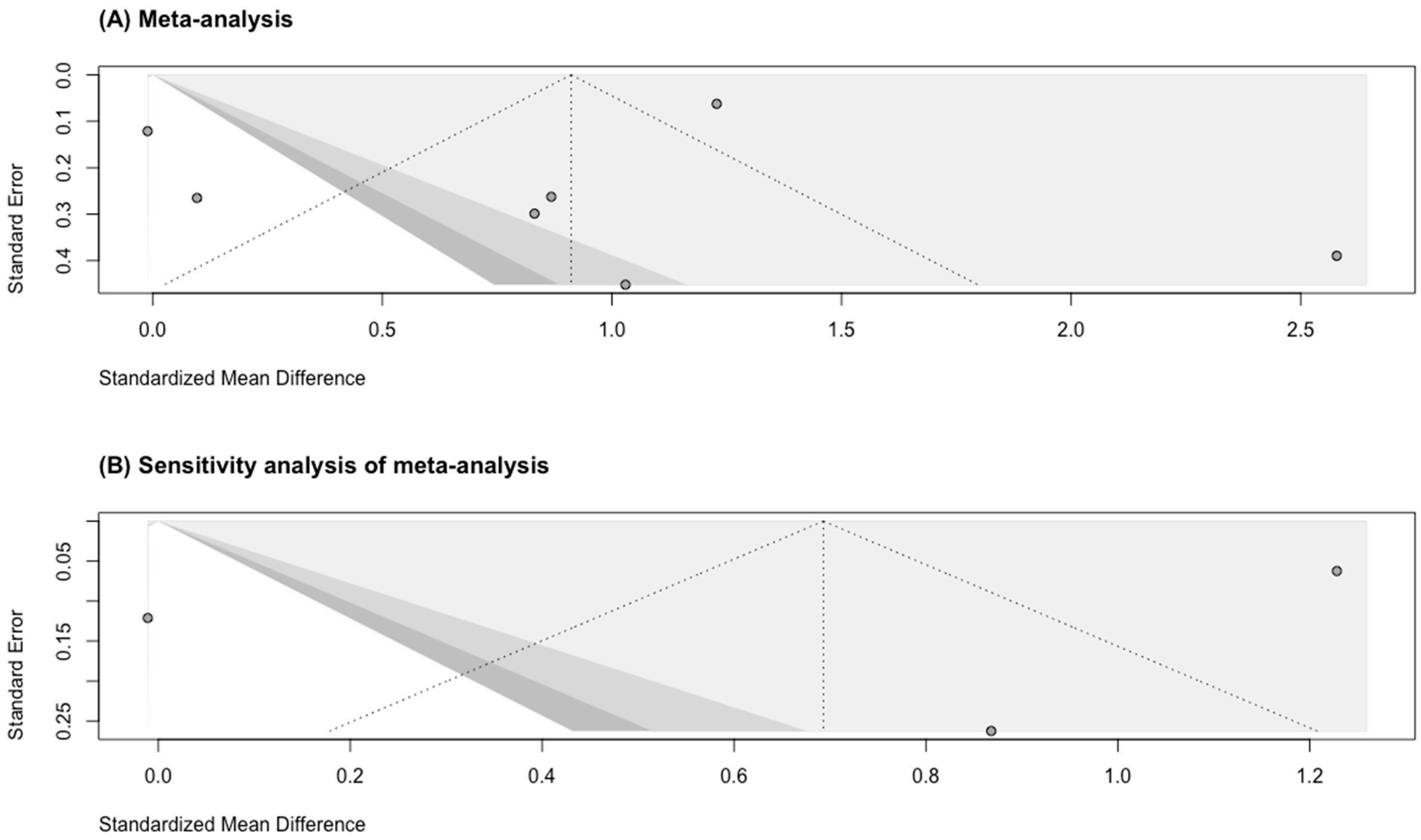

Supplementary Figure S14. Funnel plot of primary meta-analysis for exercise behavior. Each point corresponds to an included study. The x-axis indicates the standardized mean difference, whereas the y-axis indicates the standard error of standardized mean difference (SMD) for each study. The white, dark gray, medium gray, and light gray areas represent confidence intervals within 99%, 95%–99%, 90%–95%, and beyond 90% when SMD = 0, respectively. The dashed triangular vertical line and edges represent the SMD mean and 95% confidence interval in meta-analysis. When the points are symmetrically distributed on both sides of the vertical line in the triangle, publication bias is minimal.

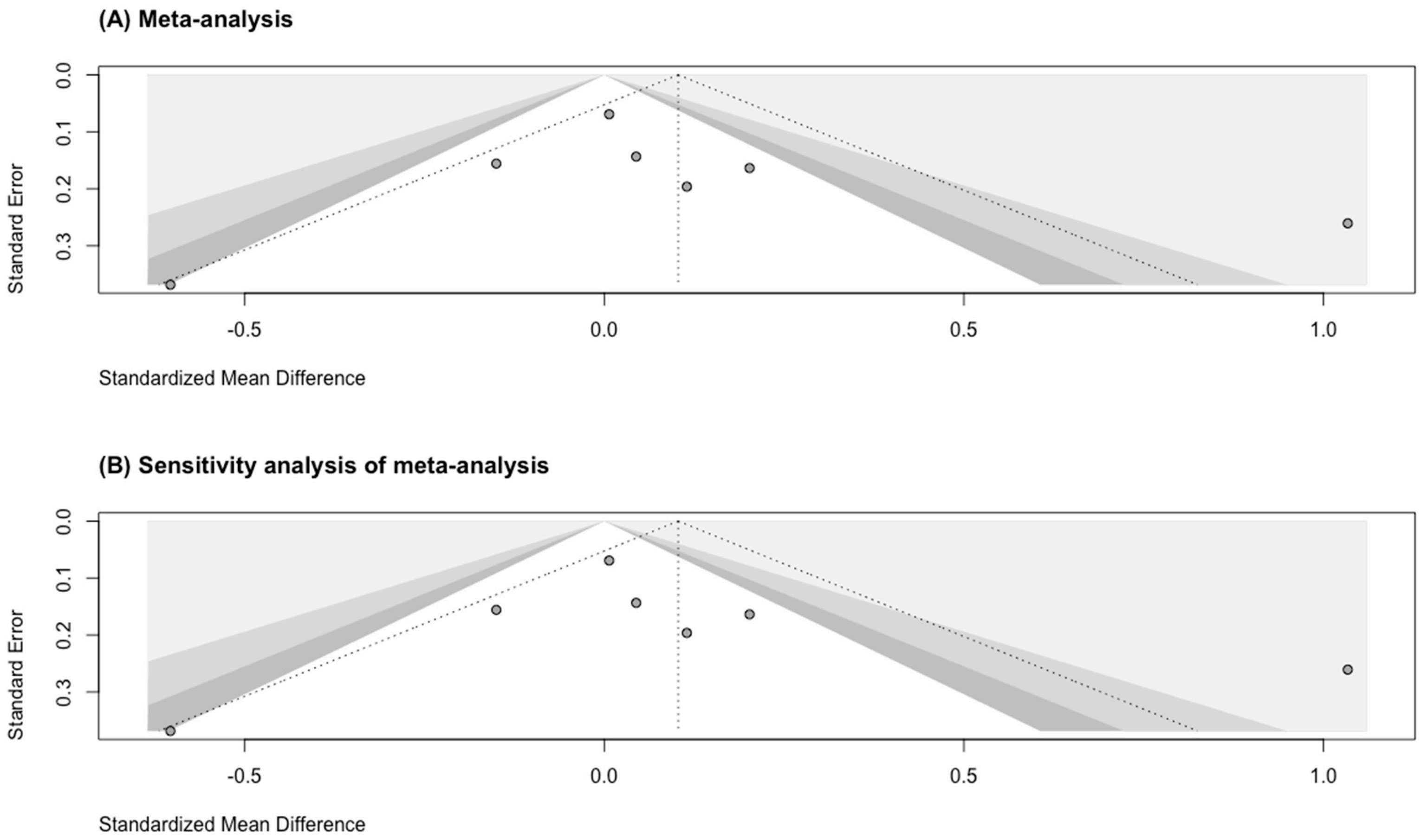

Supplementary Figure S15. Funnel plot of primary meta-analysis for work-related physical activity metric outcomes. Each point corresponds to an included study. The x-axis indicates the standardized mean difference, whereas the y-axis indicates the standard error of standardized mean difference (SMD) for each study. The white, dark gray, medium gray, and light gray areas represent confidence intervals within 99%, 95%–99%, 90%–95%, and beyond 90% when SMD = 0, respectively. The dashed triangular vertical line and edges represent the SMD mean and 95% confidence interval in meta-analysis. When the points are symmetrically distributed on both sides of the vertical line in the triangle, publication bias is minimal.

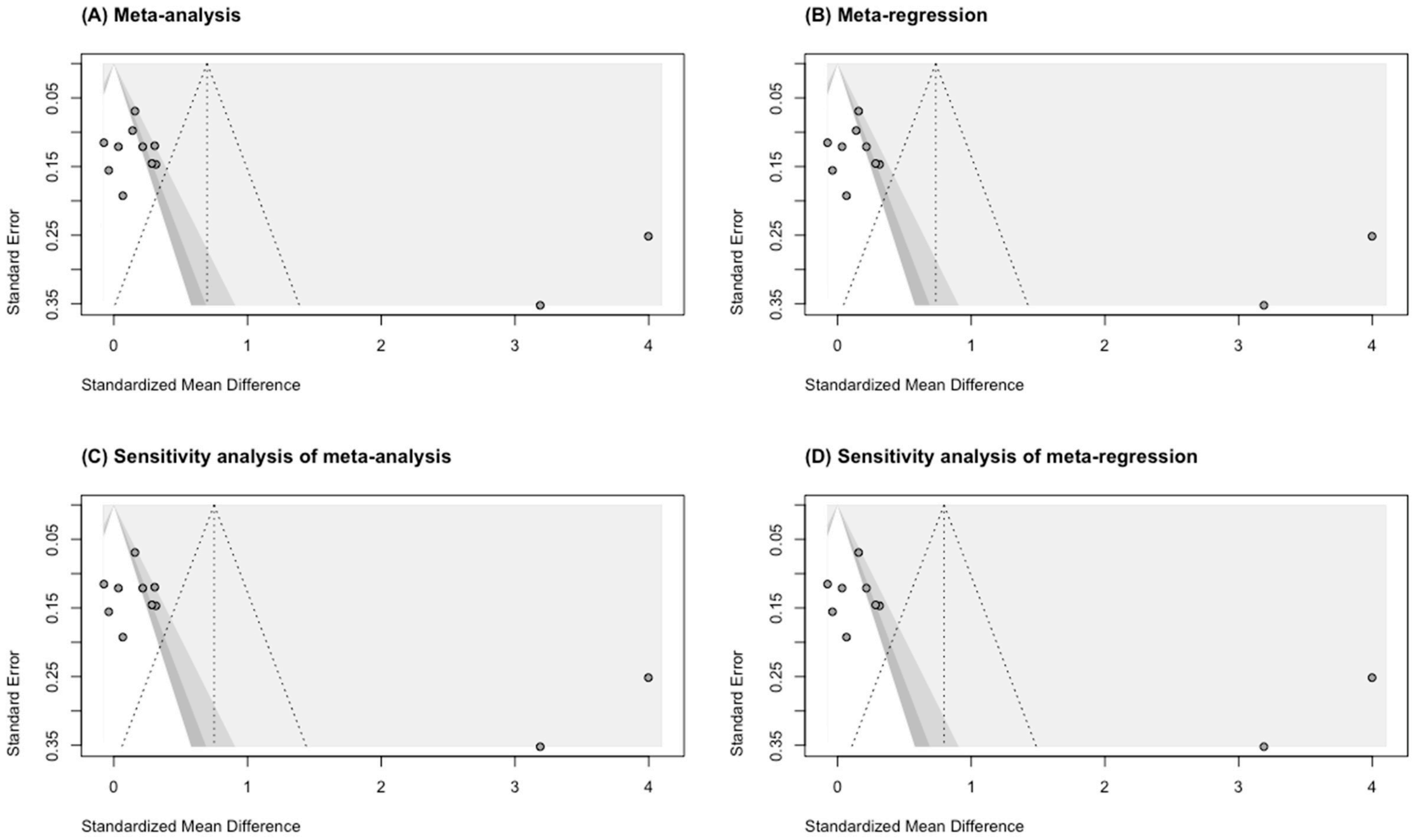

Supplementary Figure S16. Funnel plot of primary meta-analysis for work-related physical activity metric outcomes. Each point corresponds to an included study. The x-axis indicates the standardized mean difference, whereas the y-axis indicates the standard error of standardized mean difference (SMD) for each study. The white, dark gray, medium gray, and light gray areas represent confidence intervals within 99%, 95%–99%, 90%–95%, and beyond 90% when SMD = 0, respectively. The dashed triangular vertical line and edges represent the SMD mean and 95% confidence interval in meta-analysis and meta-regression. When the points are symmetrically distributed on both sides of the vertical line in the triangle, publication bias is minimal.
